# Riboflavin Supplementation Promotes Butyrate Production in the Absence of Gross Compositional Changes in the Gut Microbiota

**DOI:** 10.1089/ars.2022.0033

**Published:** 2023-02-14

**Authors:** Lei Liu, Mehdi Sadaghian Sadabad, Giorgio Gabarrini, Paola Lisotto, Julius Z. H. von Martels, Hannah R. Wardill, Gerard Dijkstra, Robert E. Steinert, Hermie J. M. Harmsen

**Affiliations:** ^1^ Department of Medical Microbiology and Infection Prevention and University of Groningen, University Medical Center Groningen, Groningen, The Netherlands.; ^2^ Department of Gastroenterology and Hepatology, University of Groningen, University Medical Center Groningen, Groningen, The Netherlands.; ^3^ School of Biomedicine, The University of Adelaide, and Precision Medicine (Cancer), The South Australian Health and Medical Research Institute Adelaide, Adelaide, Australia.; ^4^ Department of Pediatrics, University of Groningen, University Medical Center Groningen, Groningen, The Netherlands.; ^5^ DSM Nutritional Products AG, Kaiseraugst, Switzerland.; ^6^ Department of Surgery, Division of Visceral and Transplantation Surgery, University Hospital Zurich, Zurich, Switzerland.

**Keywords:** riboflavin, *Faecalibacterium prausnitzii*, microbiota, butyrate (short-chain fatty acids), insulin, bacterial networks

## Abstract

**Aims:**

We performed a randomized, placebo-controlled trial, RIBOGUT, to study the effect of 2 weeks supplementation with either 50 or 100 mg/d of riboflavin on (i) *Faecalibacterium prausnitzii* abundance, (ii) gut microbiota composition, (iii) short-chain fatty acid (SCFA) profiles, and (iv) the satiety and gut hormones.

**Results:**

Neither dose of riboflavin, analyzed separately, impacted the abundance of *F. prausnitzii*, and only minor differences in SCFA concentrations were observed. However, combining the results of the 50 and 100 mg/d groups showed a significant increase in butyrate production. While the gut bacterial diversity was not affected by riboflavin supplementation, the complexity and stability of the bacterial network were enhanced. Oral glucose tolerance tests showed a trend of increased plasma insulin concentration and GLP-1 after 100 mg/d supplementation.

**Innovation:**

Dietary supplements, such as vitamins, promote health by either directly targeting host physiology or indirectly via gut microbiota modulation. Here, we show for the first time that riboflavin intervention changes the activity of the microbiota. The butyrate production increased after intervention and although the composition did not change significantly, the network of microbial interactions was enforced.

**Conclusion:**

This RIBOGUT study suggests that oral riboflavin supplementation promotes butyrate production in the absence of major shifts in gut microbiota composition. ClinicalTrials.gov Identifier: NCT02929459.


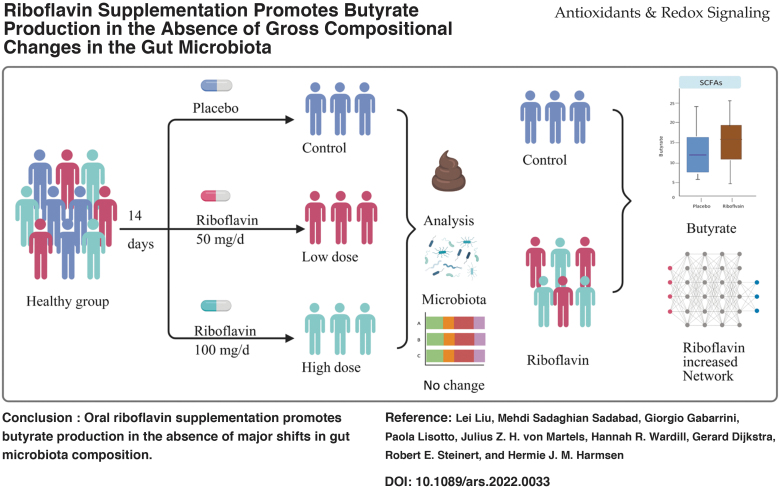


## Introduction

The human gut microbiota is increasingly associated with health (Zheng et al, 2020). Specifically, a composition with a high diversity and with a high abundance of strict anaerobic short-chain fatty acid (SCFA)–producing bacteria appears to be beneficial (Deleu et al, 2021). One of the most abundant bacteria in the human adult gut is *Faecalibacterium prausnitzii* (Barcenilla et al, 2000; Duncan et al, 2002), a butyrate-producing bacterium whose numbers are reduced in gut-related disorders such as inflammatory bowel diseases (IBDs) and obesity (Sokol et al, 2008). This reduction leads to a lower production of butyrate in the gut, a SCFA that is an energy source for enterocytes and has anti-inflammatory properties (Correa-Oliveira et al, 2016; Donohoe et al, 2011).

A possible therapy for these patients could be augmentation of these SCFA-producing bacteria (Parada Venegas et al, 2019), in particular *F. prausnitzii*, by nutrition, probiotics, or prebiotics. Vitamins that have antioxidant and redox properties offer a simple and highly accessible method of modulating the gut microbiota and its associated metabolome, and as such, have been suggested to promote health through microbial manipulation (Steinert et al., 2020; Steinert et al., 2016).

**Innovation**

The butyrate-producing *Faecalibacterium prausnitzii* is a biomarker for gut health, and riboflavin was found to be used by these bacteria as a redox mediator to shuttle electrons to oxygen, thus improving its growth. Therefore, we hypothesized that dietary riboflavin supplementation would increase its abundance to give health benefits. This clinical trial proved for the first time that riboflavin increased butyrate production, although the abundance of *F. prausnitzii* and the microbiota composition did not change. Furthermore, we observed a trend of increased secreted gut hormones that may have been triggered by enhanced short-chain fatty acids metabolic activities. Thus, vitamins that influence microbial activity and gut hormones of appetite and satiety may provide new ways to manage obesity and metabolic diseases. In addition, the bacterial network reflected more interactions after riboflavin supplementation, indicating further improvement of the microbiota. The evidence found by this study suggests that riboflavin supplementation could be a therapeutic treatment for the metabolic dysfunction in the human gut, and warrant investigation in metabolically dysfunctional cohorts.

Riboflavin, also known as vitamin B2, is required for a wide variety of cellular processes, and has an important role in maintaining health in humans (Powers, 2003). This water-soluble vitamin plays a key role in the energy and redox metabolism of both microbes and the human host (Suwannasom et al, 2020). Furthermore, studies in animal models have shown that riboflavin exerts anti-inflammatory and antioxidant effects, which can ameliorate inflammatory diseases in the gut (Biagi et al, 2020; Wu et al, 2021). Riboflavin exerts its beneficial effects on the host both directly, through absorption and systemic uptake, and indirectly via modulating the gut microbiota (Pham et al, 2021a). The latter is achieved by the redox properties of riboflavin, which creates a luminal environment with low redox potential that is highly supportive of strict anaerobic bacteria. Specifically, riboflavin serves as an electron mediator for *F. prausnitzii,* allowing it to utilize a form of anaerobic respiration by use of external electron acceptors such as cystine and even oxygen. This promotes the growth of *F. prausnitzii in vitro* at low oxygen levels (Khan et al, 2012a), and thus mimics the *in vivo* situation at the intestinal mucosa, where there is a steep oxygen gradient from the anoxic lumen across to the oxygen-rich mucosal interface (Khan et al, 2012b; Wang et al, 2012). The extracellular electron transfer using riboflavin seems to be specific to certain bacterial species, since it has been shown for *F. prausnitzii* but not other anaerobic butyrate producers such as *Roseburia* (Khan et al, 2012b). As such, the ability of *F. prausnitzii* to deploy riboflavin to shuttle electrons and thereby stimulate its growth at the mucosal interface reveals opportunities for interventions targeting this pathway to increase its abundance in the gut.

Considering the anti-inflammatory properties of *F. prausnitzii* (Flint et al, 2012) and its role in producing SCFAs, increased abundance of this bacterium may be beneficial to maintain a healthy gut and metabolism. Likewise in obesity, increased levels of SCFA acts as ligands for the free fatty acid receptors FFAR2 and FFAR3 (Kimura et al, 2020), which upon activation trigger the secretion of appetite and body weight regulating hormones such as leptin, glucagon-like peptide-1 (GLP-1), glucagon-like peptide-2 (GLP-2), and peptide YY (PYY), and cholecystokinin (CCK). As such, increasing *F. prausnitzii* abundance may be an alternative method to control obesity and associated morbidity (Brubaker, 2018; Cani et al, 2009; Everard and Cani, 2014; Steinert et al, 2017).

In a pilot intervention with healthy volunteers, we found that supplementation with 100 mg/day riboflavin for 2 weeks increased the number of *F. prausnitzii* in feces of 8 out of 11 volunteers as well as the production of butyrate (Steinert et al, 2016). In addition, riboflavin supplementation decreased the numbers of *Enterobacteriaceae,* mainly *Escherichia coli,* in the same group of volunteers, leading us to hypothesize that that riboflavin has beneficial modulatory capacities affecting the gut microbiota (Steinert et al, 2016).

Usually, dietary riboflavin is taken up in the small intestine. However, the high concentration of riboflavin used in this study should overshoot the estimated maximum small-intestinal uptake of 27 mg/d (Zempleni et al, 1996) and therefore the riboflavin will reach the colon. Since then, we performed a study with 70 IBD patients that were supplemented with 100 mg/day riboflavin for 3 weeks (von Martels et al, 2020). This resulted in a reduction in systemic oxidative stress, mixed anti-inflammatory effects, and a reduction in clinical symptoms. Fluorescence *in situ* hybridization (FISH) analysis showed decreased *Enterobacteriaceae* in patients with Crohn's Disease (CD) with low fecal calprotectin levels, though this was not observed in metagenomic sequencing analysis. Furthermore, this concept has been tried in experiments to improve the health and resistance to infections in livestock, where 50 or 100 mg/kg riboflavin supplementation in broilers over 42 days significantly modulated caca microbiota. The highest dosage was more effective, and the abundance of health-promoting bacterial groups, including *Bifidobacterium* and *Faecalibacterium*, were particularly stimulated resulting in an increased production of butyrate (Biagi et al, 2020). In addition, riboflavin supplementation of Holstein bulls improved their performance and gut microbiota (Wu et al, 2021).

In this study, we built on our previous data to investigate the effect of riboflavin supplementation in healthy individuals using a randomized, placebo-controlled trial design and two intervention dosages. In the RIBOGUT trial, a group of 105 individuals was randomized in three groups, with the primary objective to determine the effect of 2 weeks oral riboflavin supplementation (50 and 100 mg/d) on the number of *F. prausnitzii* in feces in comparison with placebo. The secondary objectives concerned the effects of the riboflavin interventions in comparison with placebo on (i) the microbiota composition and the abundance of *Enterobacteriaceae* (including *E. coli)*; (ii) the production of SCFA in feces; (iii) gastrointestinal (GI) comfort (bloating, flatulence) using validated visual analog scale (VAS) questionnaires; (iv) dry weight of the feces; (v) riboflavin concentration in feces and plasma. Furthermore, in a subgroup of participants, blood glucose, plasma insulin, GLP-1, GLP-2, and, ghrelin as well as appetite feelings using VAS during an oral glucose tolerance test (oGTT) were determined and compared with placebo.

## Results

**FIG. 1.**
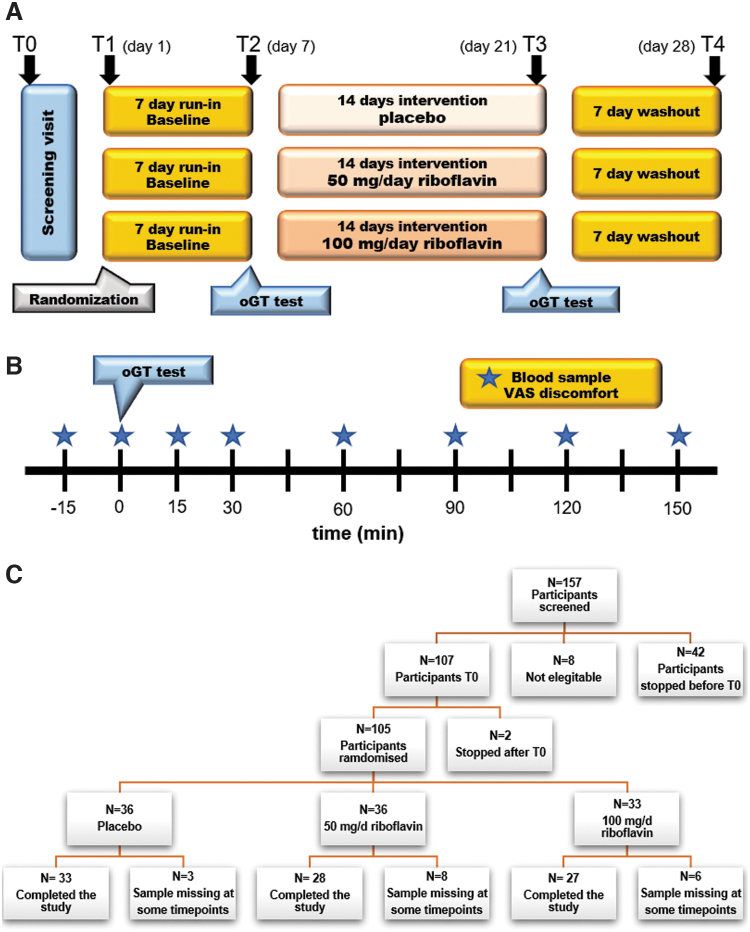
Study design for the riboflavin intervention for *N* = 105 participants **(A)**, the oGTT substudy in 36 participants **(B)**, and flowchart for the participant inclusion **(C)**. oGTT, oral glucose tolerance test.

Of the 157 volunteers who gave informed consent, 52 were excluded because of either health-related issues, body mass index (BMI) above the defined cutoff, or study dropout. The remaining 105 participants were randomized into three groups (Fig. 1). The baseline cohort characteristics are presented in Table 1. There were no differences between groups except for age with the placebo group being younger than the intervention group.

**Table 1.** Baseline Characteristics of the Study Population

Characteristics	Total	Placebo	Ribo50	Ribo100	p value
*N*	105	36	36	33	
Age (years)	31.1 [11.8]	27.2 [9.4]	33.3[13.0]	33.0 [11.8]	0.04^*^
Sex (male)	26 (24.8%)	9 (25.0%)	9 (25.0%)	8 (24.2%)	0.99
Height (cm)	170.73 [10.26]	168.81 [12.37]	171.49 [9.68]	172.00 [8.12]	0.38
Weight (kg)	63.80 [9.55]	64.23 [8.55]	63.60 [10.41]	63.56 [9.89]	0.95
BMI (kg/m^2^)	21.82 [2.03]	22.57 [2.17]	21.61 [1.84]	21.37 [1.93]	0.07

^*^ Shows significance.

Data presented as numbers [mean [SD], proportions *n* (%)]. Differences between groups were tested with independent samples ANOVA test or the Kruskal–Wallis test for non-normally distributed continuous variables, as appropriate. Two-sided *p* values <0.05 were considered as statistically significant.

### Effects of riboflavin supplementation on the abundance of *F. prausnitzii* and other gut anaerobes

**FIG. 2.**
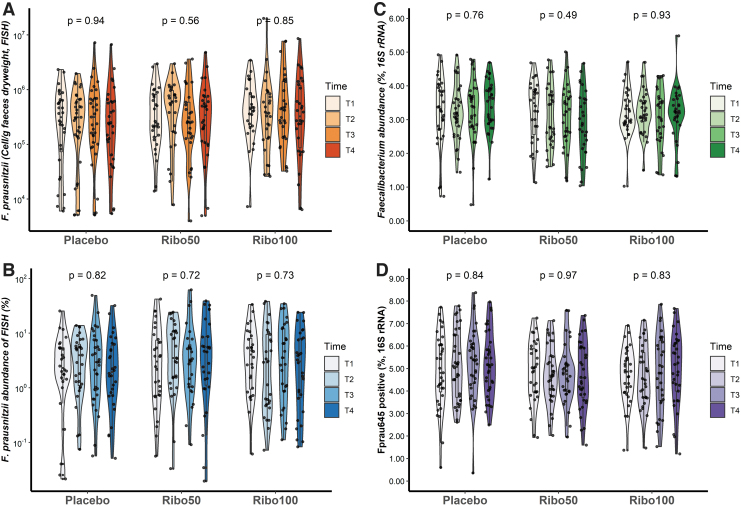
The results of *Faecalibacterium* quantification based on FISH with probe Fprau645 and 16S rRNA gene sequencing. **(A–C)** The abundances of *F. prausnitzii* from FISH **(A)**, the relative abundances **(B)**, and 16S rRNA gene sequencing **(C)** were not changed over all time points among three groups (*p* > 0.05 for all groups, Kruskal–Wallis test). **(D)** The relative abundances of ASVs from 16S rRNA gene sequencing, which corresponded to the probe Fprau645, were not changed. ASV, Amplicon Sequence Variant; FISH, fluorescence *in situ* hybridization.

Riboflavin supplementation did not increase the relative abundance of *Faecalibacterium*, as determined by FISH (Fig. 2A, B). More specifically, the quantity and percentage of *F. prausnitzii* were not affected by riboflavin. The relative amount of *Faecalibacterium* in all samples is depicted in Figure 2C, and shows that there was no significant change in the number of *Faecalibacterium* at genus level in all groups among all time points. Despite the rigorous randomization, differences were observed in baseline abundances of *Faecalibacterium,* therefore no results of comparisons between groups we interpreted as significant. In addition, the relative abundance of ASVs from 16S rRNA gene sequencing, which contains the target site for the FISH probe Fprau645, was calculated (Fig. 2D), confirming the FISH results, which indicated no effect of riboflavin on *F. prausnitzii* abundance when compared with placebo.

In addition, we did not observe any effect of riboflavin supplementation at both doses on relative abundance of *Clostridium* group XIVa (now referred to as the family *Lachnospiraceae*) and *Roseburia* when compared with placebo (Supplementary Fig. S1A–D). In addition, no significant change in dry weight of the feces was observed, nor did the number of the aforementioned bacteria per gram dry weight change with the intervention.

The relative abundance of potentially pathogenic *Enterobacteriaceae* (Supplementary Fig. S1E, F), which includes *E. coli*, also did not show any change after supplementation as well as the ratio (Supplementary Fig. S1G, H) between *Enterobacteriaceae* and *Faecalibacterium* in all groups at all time points. Participant and sample characteristics, and FISH data are presented in Supplementary Table S1.

### Effects of riboflavin supplementation on gut microbiome diversity

Based on the 16S rRNA gene sequencing of samples collected at each study visit, the microbial community structure was characterized and quantified by means of microbial diversity. The alpha diversity (shannon index and phylogenetic diversity (PD_whole_tree)) was calculated and presented in Supplementary Table S2. Riboflavin intervention did not induce significant changes in α diversity of the gut microbiota.

**FIG. 3.**
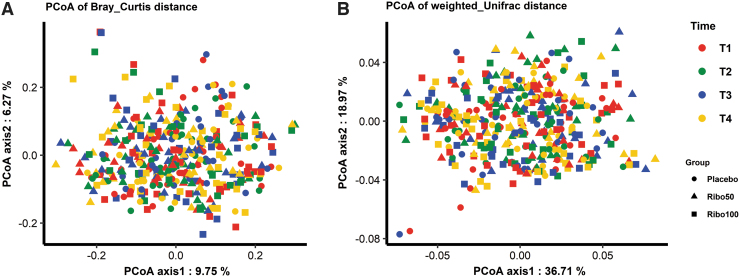
PCoA of the distances between taxonomical composition based on the Bray-Curtis **(A)** and weighted-Unifrac **(B)** over all time points of all three groups. Each *dot* represents a sample separated by color code and shape as indicated in the legend. PCoA, principal coordinates analysis.

**FIG. 4.**
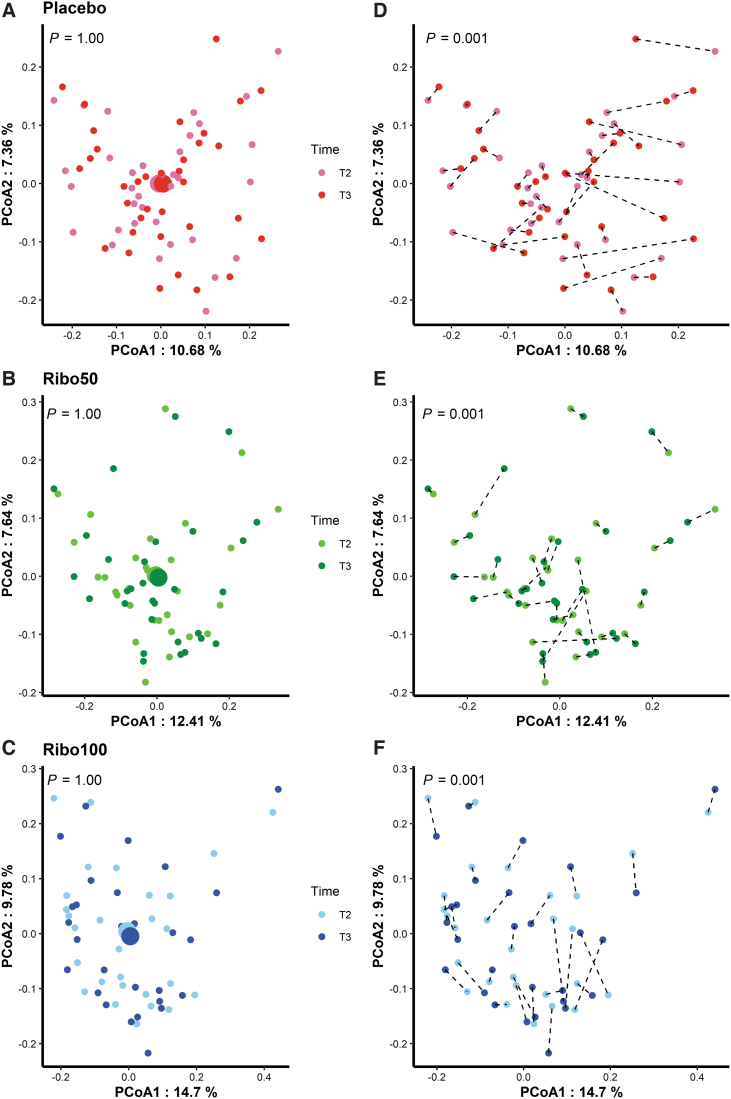
PCoA of individual's taxonomical composition based on the Bray-Curtis distance between T2 and T3. **(A–C)**, samples' taxonomical composition in each group compared between T2 and T3; **(D–F)**, paired samples' taxonomical composition in each group compared between T2 and T3. Each *dot* represents a sample, and the *dashed lines* indicate the fecal samples from the same individual.

Principal coordinates analysis (PCoA) was calculated over all datasets based on the Bray-Curtis and weighted-Unifrac distances (Fig. 3) with no clear separations. No significant differences of taxonomical composition between participants before and after riboflavin supplementation in each group were found (*p* = 1.00, for all groups; Fig. 4A–C). In addition, taxonomical composition of paired samples in each group was evaluated, showing that the differences of individual samples contributed to the variances of taxonomical composition significantly (*p* = 0.001, for all groups; Fig. 4D–F), independent of the intervention.

A separation into two clusters was observed from the PCoA of unweighted-Unifrac distance at T1 (Supplementary Fig. S2A) and after 2 weeks of riboflavin supplementation, samples per volunteer remained in their own cluster (Supplementary Fig. S2B). The two clusters found were correlated with the PD_whole_tree index and the relative abundance of *Methanobrevibacter*. This division into two clusters was based on the presence and amount of *Methanobrevibacter*; one group in low and the other in high numbers of this archaea (Supplementary Fig. S2C). The PD_whole_tree index and abundance of *Methanobrevibacter* of cluster 2 were significantly higher than cluster 1 (both *p* < 0.001; ANOVA test; Supplementary Fig. S2D). Based on the two clusters, samples significantly separated, *p* < 0.001, based on the Bray-Curtis and weighted-Unifrac distances, respectively (Supplementary Fig. S3).

### Riboflavin supplementation increased fecal SCFAs concentration

**FIG. 5.**
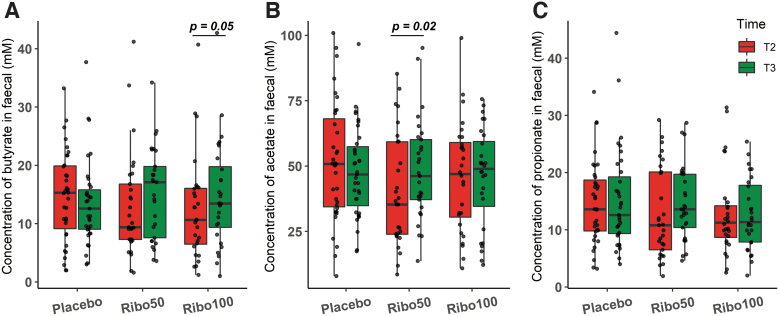
Boxplots of SCFAs concentrations of paired fecal samples significantly increased after 2 weeks of riboflavin supplementation in pairwise comparison. **(A)** Butyrate concentration increased significantly in Ribo100 group (*p* = 0.05, Wilcoxon signed-rank test); **(B)** acetate concentration increased significantly in Ribo50 group (*p* = 0.02, Wilcoxon signed-rank test); **(C)** no significant change was observed with propionate concentration. Each *dot* represents a sample, and horizontal bar indicates significant differences. SCFA, short-chain fatty acids.

Riboflavin supplementation changed fecal concentration of the SCFAs butyrate, propionate, and acetate. Comparing T2 and T3, we found a significant increase in the concentration of butyrate in group Ribo100 and the concentration of acetate in group Ribo50 (*p* = 0.05, *p* = 0.02, respectively; Wilcoxon signed-rank test; Fig. 5). In the Ribo50 group, a trend was observed for increased butyrate and propionate concentration; however, this was not significant (*p* = 0.11, *p* = 0.10, respectively; Wilcoxon signed-rank test).

**FIG. 6.**
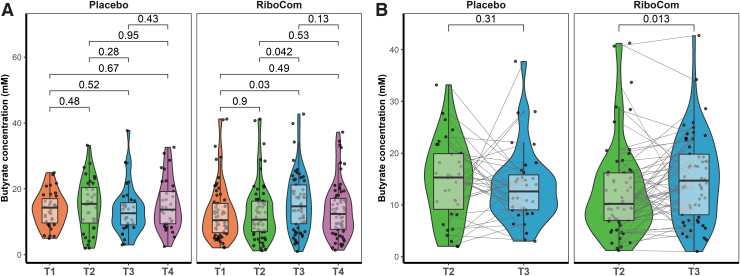
Fecal butyrate concentration was increased in combining data of two doses riboflavin intervention groups. **(A)** Comparison within placebo and RiboCom groups (Mann–Whitney *U* test). **(B)** Pairwise comparison of samples T2 and T3 within placebo and RiboCom groups (Wilcoxon signed-rank test). Horizontal bar indicates adjusted *p*-values.

Besides the original analysis in three groups, which only resulted in minor significant effects, an *ad hoc* analysis was performed on the SCFA data of the RiboCom group: a combination of both riboflavin intervention groups. This combined group enabled the comparison of a higher number of samples before and after intervention, essentially increasing power. This comparison showed a significant increase of butyrate concentration after riboflavin intervention, both as group, in the Mann–Whitney *U* test (T3 *vs*. T2, *p* = 0.042; T3 *vs*. T1, *p* = 0.03; Fig. 6A), as well as pairwise comparison using the Wilcoxon signed-rank test (*p* = 0.013; Fig. 6B).

In addition, when the SCFA levels of all samples were correlated with the bacterial composition, we observed a significant correlation between butyrate and the relative abundance of *Faecalibacterium* (*R* = 0.26, *p* < 0.001; Supplementary Fig. S4A). The Pearson correlation between butyrate and top 30 abundant bacterial genera showed a positive correlation of the butyrate concentrations with various *Firmicutes*, some known to produce butyrate, others known to produce other SCFA (*e.g., Streptococcus*), suggesting a cross-feeding correlation.

Furthermore, butyrate correlated negatively with *Bacteroides*, *Alistipes*, *Parabacteroides*, and, remarkably, *Clostridium_IV*, *Flavonifractor,* and *Oscillibacter* of the *Oscillospiraceae* family to which *Faecalibacterium* belongs as well (Supplementary Fig. S4B) (Tindall, 2019). Of note, butyrate-producing bacteria such as *Faecalibacterium* also correlated with other SCFA levels and total SCFA levels. However, no differences in the relative abundance of *Faecalibacterium* over all time points in each group were found as mentioned above, nor in pairwise analysis of samples from the same individual between T2 and T3 (Supplementary Fig. S4C). Notably, analyzing all genera that correlated most significantly with butyrate levels showed no response to the interventions with riboflavin, neither with intervention groups individually nor with the combined RiboCom group (Supplementary Fig. S4D).

### Riboflavin intervention enhanced bacterial networks

**FIG. 7.**
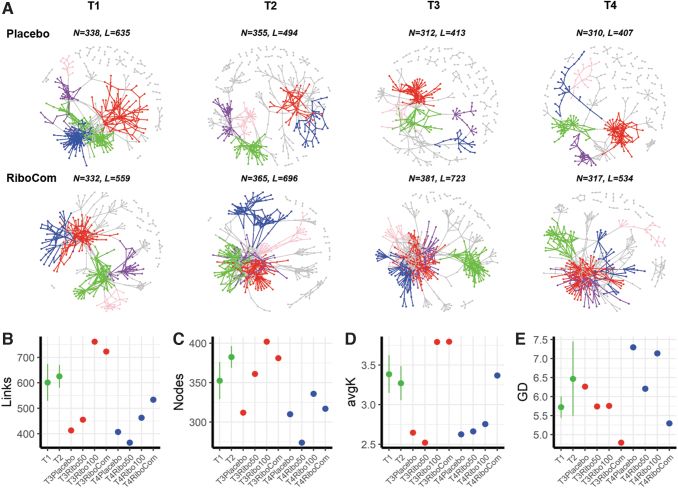
Gut bacterial networks over all time points. **(A)** Visualization of constructed MENs of placebo (*top*) and RiboCom groups (*bottom*) from T1 to T4. Top 5 large modules are shown in different colors, and smaller modules are shown in *gray*. Each network is shown based on the Pearson correlations (RMT-threshold 0.67, FDR adjusted *p* < 0.05) between the abundances of bacterial ASVs of 36 placebo samples and 69 RiboCom samples. N, nodes; L, links. **(B–E)**, links **(B)**, nodes **(C)**, average degree (avgK) **(D),** and average path distance (GD) **(E)** are topological features of gut microbial networks. The baselines of T1 and T2 were calculated as means ± SE, and the values of T3 and T4 networks shown as *dots*. MEN, molecular ecological network; RMT, random matrix theory.

To find the explanation for the increased butyrate concentration in the RiboCom group, we analyzed the bacterial network for the potential interactions between microbial taxa. This ecological network tool enables us to decipher the structure of the microbiota over time (Shi et al, 2022). Key topological features of gut bacterial network correlation were calculated from the individual groups as well as the RiboCom group as shown in Figure 7 and Supplementary Figure S5. The network complexity, as indicated by nodes, links, and density, was increased in the Ribo100 and the RiboCom group at T3 with riboflavin intervention, and different from that of the placebo and the Ribo50 group.

The average clustering coefficient (avgCC) of all empirical networks was higher than the corresponding random networks, indicating specific interactions in the empirical networks. Modularity values were >0.4, indicating that the constructed networks had modular structures consistent with the avgCC. In addition, the stability of network was improved with increased average degree (avgK) and decreased average path distance (GD) after riboflavin supplementation.

The clusters were analyzed for network seed nodes and connections, to determine their correlations between each other. The first cluster of RiboCom group consisted of a butyrate-producing genus *Anaerostipes* as seed, which connected to other genera before the intervention. However, after intervention the first cluster consisted of butyrate-producing *Roseburia* as seed connected to other genera including acetate producers. Information of the top five network clusters before and after intervention is available in Supplementary Table S3.

### Concentration of riboflavin was increased in feces and plasma

**FIG. 8.**
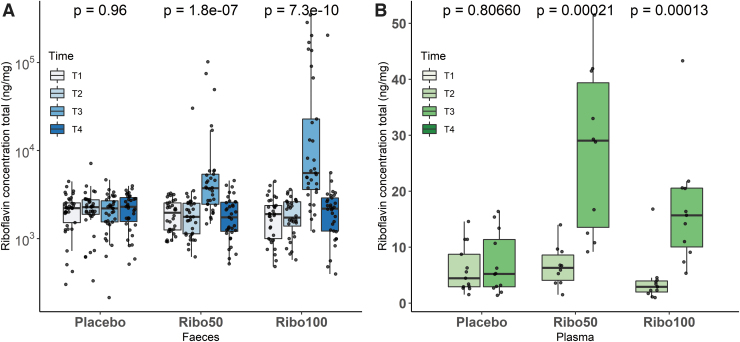
Boxplots of riboflavin concentrations in feces and plasma samples. **(A)** Riboflavin concentration in fecal samples was significantly increased after intervention at T3 (*p* < 0.05, Kruskal–Wallis test). **(B)** Riboflavin plasma levels of the oGTT groups significantly increased at T3 in both riboflavin supplementation groups (*p* < 0.05, Mann–Whitney *U* test). No differences in the placebo group were found in either fecal or plasma samples.

The riboflavin concentration was measured in both feces and plasma. Increased fecal riboflavin was observed in both group Ribo50 and Ribo100 after 2 weeks of riboflavin supplementation as well as in plasma of the oGTT group (Fig. 8). The riboflavin concentration of placebo group did not change over time in both feces and plasma.

### Effects of riboflavin on glucose homeostasis and appetite perceptions during an oGTT

**FIG. 9.**
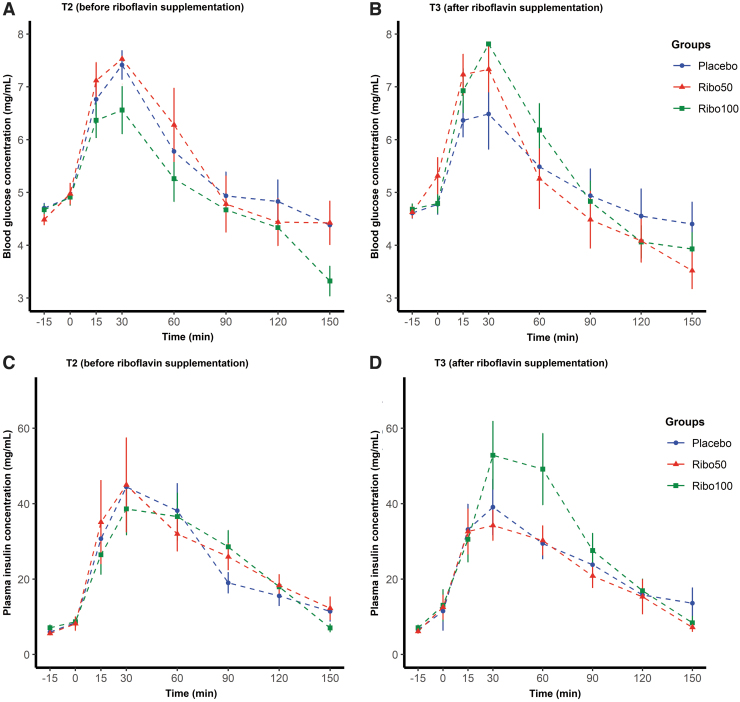
Oral glucose tolerance test results of plasma glucose **(A, B)** and insulin concentration **(C, D)** at T2 before riboflavin supplementation **(A, C)** and T3 after riboflavin supplementation **(B, D)**.

Interestingly, riboflavin appeared to impact postprandial plasma insulin responses in a subgroup of participants receiving 100 mg/d (Ribo100 group). More specifically, there was a trend for elevated plasma insulin concentration between 15 and 90 min when compared with placebo and the Ribo50 group (Fig. 9). However, this trend did not reach statistical significance. Furthermore, the area under the curve was calculated, but no significant difference between the treatments was observed. No effect was seen for plasma glucose concentrations, remaining unchanged in response to the interventions. There was also a trend for an increase in plasma GLP-1 between 15 and 120 min, but not GLP-2 or ghrelin (Supplementary Fig. S6), when compared with placebo and Ribo50 groups. There was no effect of riboflavin on appetite perception during the oGTT (data not shown).

## Discussion

*F. prausnitzii* is consistently associated with health-warranting investigation of methods to increase its abundance or functionality (Gacesa et al, 2022). Previous data from our group and others show that riboflavin is well positioned to support *F. prausnitzii* growth (Khan et al, 2012b; Steinert et al, 2016; von Martels et al, 2020). As such, we sought to investigate this in a placebo-controlled randomized trial of 2-week supplementation with 50 and 100 mg/d riboflavin on gut microbiota. The primary objective of the study, an increase in the amount of *F. prausnitzii*, was not reached. The bacterial load of this butyrate producer did not significantly increase in the riboflavin intervention groups as measured by FISH analysis and 16S rRNA gene sequencing. In addition, differences in microbiota diversity were not observed during the 2-week intervention among all study groups. However, after *ad hoc* analysis we found an increased butyrate level in the riboflavin combination group and an enhanced bacterial network after riboflavin intervention. Furthermore, a trend of increased insulin and GLP-1 concentration was observed in the 100 mg/d group. The health status of volunteers was accessed by means of measuring clinical blood parameters (Supplementary Table S4), and no adverse events were reported, including gastrointestinal health.

In our previous *in vitro* study, riboflavin stimulated the growth of *F. prausnitzii* under oxygenated condition via extracellular electron transfer (Khan et al, 2012b). This, in combination with the results of the pilot study, where an increase of *F. prausnitzii* was found in 8 of 11 volunteers, prompted us to perform a randomized clinical trial to comprehensively assess the effect of high-dose riboflavin supplementation in healthy subjects (Steinert et al, 2016).

In addition, we initiated a study investigating the effect of riboflavin intervention in patients with IBD (RISE-UP), which identified a number of benefits for riboflavin intervention (von Martels et al, 2020). The need to expand this work in a healthy population of people lies in the relative stability of their gut microbiota, which is less likely to be influenced by (low grade) inflammation and/or diarrhea compared with people with IBD (Gacesa et al, 2022; Sipponen and Kolho, 2015). Furthermore, while the rationale to study the effect of vitamins in people with certain diseases is clear, there are potential benefits that can be gained from stabilizing or improving the microbiota of healthy individuals. For instance, increasing microbial stability may increase host resistance to gut-related diseases and infections, for instance by viruses (Sarkar et al, 2021).

The primary source of riboflavin is diet, especially from dairy consumed in the Western World. However, it appears that gut bacteria themselves can also provide additional riboflavin (Soto-Martin et al, 2020), which is of particular importance for the nonriboflavin-producing *F. prausnitzii* strain A2-165, which can benefit via cross-feeding from other members of the microbial community. Although the diversity of riboflavin-rich foods available is enormous, the poor riboflavin status in the Western countries is still a concern (Powers, 2003).

Here, we gave a high-dose riboflavin, which was higher than the absorption capacity (Zempleni et al, 1996), in an attempt to saturate the system and avoid under dosing. However, it is possible that *Faecalibacterium* in healthy people has already sufficient riboflavin, and further saturation in the gut did not change their relative abundances. Furthermore, this 2-week intervention (*vs*. 3 weeks in the RISE-UP) may not have been long enough to alter the composition of the gut microbiota. While no change in composition was observed, the microbiota appeared more functionally active with changes in the SCFA production observed, in particular butyrate. A previous study by Pham et al (2021b) showed vitamin treatments induced changes in the metabolic activity of the gut microbiome *in vitro*, and found that 75 mg/d riboflavin increased butyrate concentrations when compared with the control sample.

In addition, we observed that the intervention enhanced the complexity and stability of the microbial networks, indicating a change in metabolism and more cross-feeding interactions, which may give a basis for the increased butyrate production in the absence of gross compositional shifts (Flint et al, 2008). Of note, increased interactions between acetate producers (*e.g., Blautia*) and butyrate-producing bacteria were observed, since most butyrate producers are utilizers of acetate, which is normally regarded as an end product of anaerobic fermentation (Duncan et al, 2004).

While previous data suggest that a short intervention time is sufficient for microbial change (Walker et al, 2011), intervention in animal studies suggests that a longer intervention time may be required. For example, in broiler chicks fed with 100 mg/kg riboflavin for 6 weeks, a significant change in the cecal microbiota was reported with increased bifidobacteria and elevated butyrate concentrations (Biagi et al, 2020). In a study with Holstein bulls, dietary supplementation for 4 periods of 24 days with 900 mg/d per bull of riboflavin had positive impacts on digestion of nutrients, rumen total volatile fatty acid production, microbial growth, and enzymatic activity, although no further effects on performance were observed (Wu et a., 2021). While this may reflect more intensive intervention, the differences in outcome between these human and animal experiments may also be caused by the fact that the latter are more controlled.

When considering our findings, it is also important to appreciate riboflavin uptake and biodistribution. A large part of riboflavin will be taken up in the small intestine, our high dosages, however, will ensure that it reaches the colon (Zempleni et al, 1996). Here, riboflavin is able to theoretically exert more profound effects on the gut microbiota, being more abundant in the colon. Analysis of fecal samples from our study cohort confirmed that riboflavin was indeed higher in our intervention groups compared with placebo. Although riboflavin reaches the colon, a more targeted colon delivery by enterically protected capsules may give a better response at lower concentration by avoiding the interfering effects of small intestinal uptake by the host (Broesder et al, 2020).

Notably, microbiota PCoA of this study data did show remarkable division of participants independent of riboflavin intervention based on unweighted-Unifrac distance. This division was based on the participant's microbiota, showing a signature composition indicating the presence of methane-producing bacteria in the host. Our results confirm earlier reports on the methane production being very personalized, with some individuals producing methane while in others methane presence is not detectable; a trait largely related to health (Ghoshal et al, 2016; Knobbe et al, 2020).

Here, we show that the presence of methanogens is stable over time and is not influenced by riboflavin. Furthermore, the PCoA of paired samples showed that there was variance in the taxonomical composition between T2 and T3 on an individual level. However, the compositions of the studied individuals changed randomly, independently from intervention, which was most likely caused by individual daily diet variations.

Although significant changes were only detected on an individual level and seem randomly spread over all three groups, network analyses showed that in comparison with T1, minor changes in bacterial networks were found at T2. It may be speculated that this is an adaptation of the participants to the trial conditions and an increased awareness of being monitored. This random fluctuation is shown as variation in networks during time points T1 to T2 in Figure 7 and Supplementary Figure S5.

The effect of high-dose riboflavin intervention at T3 clearly exceeded this variation suggesting an enforced complexity and stability of the network, which in turn gives an indication for enhanced ecological interactions (Chen et al, 2022; Shi et al, 2022). Bacteria that cross-feed other communities also benefit from the supplied riboflavin and make stronger interactions with others. The bacterial networks will reflect interactions among microorganisms (Shi et al, 2022), which provides new insights into how riboflavin affects the microbiota. Further research should therefore aim at understanding the functional implications of improved networks.

It has been shown that increases in the production of SCFAs, which act as ligands for the free fatty acid receptors FFAR2 and FFAR3, can trigger the secretion of appetite and body weight regulating hormones such as GLP-1 and PYY (Everard and Cani, 2014; Steinert et al, 2017). A paper by Mahalle et al (2014) has found that intake of vitamins including riboflavin and minerals was negatively correlated with HOMA (Homeostatic model assessment), insulin resistance, and inflammatory markers. However, our analyses showed no significant effect of riboflavin on gut intestinal hormones, and did not impact oral glucose tolerance and appetite perception, although a trend in increased insulin production and GLP-1 was noted after 100 mg/d supplementation. Expansion of the study in size and duration could have given more statistical power to show this effect.

In interpreting our data, a number of limitations require consideration. As mentioned above, the 2-week intervention may have been too short, and the participants may have already had an optimal microbiota composition that was resilient to change. This is also reflected in the low number of *Enterobacteriaceae* detected, with both FISH and 16S rRNA gene sequencing. Therefore, with the ratio between *Enterobacteriaceae* and *Faecalibacterium* already very low it is unlikely that it could not be improved (ceiling effect). This is in stark contrast to the RISE-UP study in IBD patients, where riboflavin resulted in an improvement in patients. In addition, the baseline gut microbiota composition showed substantial variation, which limited the outcome of this study. The number of included individuals was based on our pilot study (Steinert et al, 2016), but a larger scale study might be more effective in equalizing the groups.

Furthermore, we failed to conduct all intended analyses of the oGTT volunteers due to technical and clinical issues, which made this group underpowered to draw firm conclusions. Moreover, we used a standard oGTT to stimulate GLP-1 secretion. Although the oGTT has been used in various populations under various study conditions to investigate GLP-1 secretion (Steinert et al, 2017), a standardized mixed meal test may have been better to investigate effects on appetite in response to the investigation.

This randomized placebo-controlled study, designed to show the effect of riboflavin in healthy volunteers on the abundance of *F. prausnitzii*, clearly indicated that there is no increase after a 2-week intervention with either of the two concentrations. The dataset generated here did show the stability of the microbiota over 4 weeks with riboflavin intervention, and more importantly, the gut microbiota seemed to be activated producing more SCFAs. The results of this clinical trial confirmed that an oral riboflavin supplementation up to 100 mg/d is safe for use during a 2-week period with no reported serious adverse effects.

In conclusion, the RIBOGUT study showed that a 2-week intervention with a high-dose oral riboflavin had no effect on the gut microbiota composition, but increased its activity as shown by higher butyrate concentration in the fecal samples.

## Materials and Methods

### Study population

A group of 105 males and females in healthy weight range (BMI 18–25 kg/m^2^), aged 18–60 years were included and randomly assigned into three groups. A medical prestudy examination was used to verify that participants were qualified for study inclusion. Main exclusion criteria were as follows: gastrointestinal disorders; abnormal clinical chemistry and hematology; psychological illness; pregnancy or lactation; use of antibiotics, probiotics, or multiple vitamin supplements and micronutrients during the study; being on specific hypocaloric or hypercaloric diets; history of cancer or substance abuse; allergy or sensitivity to any ingredients of the study product.

### Data collection and study design

This intervention study (ClinicalTrials.gov Identifier: NCT02929459, Registered October 11, 2016, https://clinicaltrials.gov/ct2/show/NCT02929459.) was approved by the institutional review board of the University Medical Center Groningen under number METc-UMCG2015.510. It was a randomized, placebo-controlled, double-blind, parallel-group trial, and was preceded by a run-in period of 7 days. Thereafter, the first group took a placebo for 2 weeks (defined as “placebo”), the second group of volunteers ingested a dose of 50 mg riboflavin once a day at breakfast for a period of 2 weeks (defined as “Ribo50”), and the third group of volunteers ingested a dose of 100 mg riboflavin once a day at breakfast for a period of 2 weeks (defined as “Ribo100”).

We power calculation, and the dose was based on our pilot study using 100 mg/d for 14 days (Steinert et al, 2016). All doses were oral capsules and were indistinguishable from each other. The intervention period was followed by a washout period of 7 days. The total study duration was 28 days.

An electronic Case Report Form (eCRF) data capture system was generated (KOEHLER eClinical GmbH, Germany), and all data per participant were recorded in an eCRF except for the microbiota sequencing data. We used the exported eCRF data for statistical analysis as reported here.

Participants received stool kits for the collection of fecal samples. Fecal samples were collected by participants and stored in their home freezers immediately after defecation. Samples of baseline (day 1, T1) and after 7 days run-in (day 7, T2) were collected before the intervention. After 2 weeks of supplementation with riboflavin/placebo (day 21, T3) and 7 days washout (day 28, T4), additional fecal samples were collected, as shown in detail in Figure 1. All frozen fecal samples were transported to the University Medical Center Groningen (UMCG; Groningen, the Netherlands) in ice packs, put on dry ice for internal transport, and subsequently stored at −80°C without cryopreservation. All samples were subjected to storage for 1 year ±4 months at −80°C before sequence analysis. Gastrointestinal comfort was assessed using a Rome III criteria questionnaire, and blood samples were collected and stored at −80°C before (T0) and after (T3) riboflavin/placebo supplementation for clinical chemistry and hematology measurements.

A subgroup of subjects (the intention was to include *n* = 15 from each group) participated in an oGTT, which was performed according to standard procedures (Choi et al, 2013; Ma et al, 2005) at T2 and T3. These participants arrived at the study site at 9 a.m. after an overnight fast (≥10 h), and after having consumed a standardized dinner between 6 p.m. and 7 p.m. the night before. Participants were allowed to consume water *ad libitum*, but not later than 12 p.m. the day before the study day. No alcohol was to be consumed 24 h before arrival at the study site. Participants were instructed to avoid vigorous physical activity (*e.g.,* prolonged or demanding runs, *etc.*) for 24 h before arrival at the study site. On the study day, after the usual check for vital signs and blood collection for assessment of biomarkers of gut health, participants received an oral glucose load of 75 g dissolved in 300 mL of water.

Venous blood samples (5 mL) were drawn 15 min before and directly after ingestion of the glucose load, and 15, 30, 60, 90,120, and 150 min postingestion. Immediately, samples were centrifuged (800 *g*, 10 min, 4°C) and stored frozen at −80°C. Therefore, fasting and postprandial blood samples (8 × 5 mL) were taken at regular time intervals in response to an oGTT as outlined in Figure 1 for measurement of plasma GLP-1, GLP-2, and ghrelin, riboflavin (fasting only) and insulin and blood glucose. In addition, appetite perception (hunger, satiety, fullness, *etc.*) was assessed by using 100-mm VAS, with words anchored at each end, expressing the most positive and most negative ratings.

### Investigational product

The riboflavin/placebo supplements were produced by DSM Nutritional Products, stored in the storage of the UMCG microbiology department until delivery to each participant at day 7 of the trial. Participants received daily riboflavin/placebo supplementation of the normal diet for a period of 2 weeks. Additional information on the capsule is included in the Supplementary Data.

### SCFAs analysis

Concentrations of SCFAs in the fecal samples were analyzed with GC-MS (Gas Chromatography-Mass Spectrometry). Method details were described previously (von Martels et al, 2020).

### FISH analysis

FISH was performed using an automated device (Biotrack analyzer; Biotrack B.V.; Leeuwarden, Netherlands). Fluorescent probes for *F. prausnitzii* (Fprau645), *Clostridium* group XIVa (Erec482), *Roseburia* (Rint623), *Enterobacteriaceae* (EC1535), and total bacteria (EUB338) were used to measure the effect of the riboflavin intervention (Benus et al, 2010; Manichanh et al, 2006). The probe sequences are available in the Supplementary Data.

### Blood analysis

Blood was collected by qualified research assistants using a standardized protocol (Supplementary Data). Blood for chemistry and hematology measurements was analyzed by the UMCG. Plasma glucose concentration was measured on an YSI 2300 Stat glucose/lactate analyzer (YSI, Inc., Yellow Springs, OH). Serum insulin concentration was measured using radioimmunoassay (Diagnostic Laboratories, Los Angeles, CA) (Cree et al, 2008). Total GLP-1, GLP-2, and ghrelin were analyzed at DSM Nutritional Products, Switzerland, by using commercially available ELISA kits (Millipore Corp).

### DNA extraction, PCR, and 16S rRNA gene sequencing

DNA was extracted from 0.25 g fecal sample using QIAamp DNA Stool Mini Kit (Qiagen, Hilden, Germany). Modified barcoded 341F and 806R primers (Supplementary Data) were used to amplify the V3–V4 region of the 16S rRNA gene. Details on PCR, barcoded primers, and sequencing library preparation were described previously (Heida et al, 2016). The amplicons were sequenced with a MiSeq Illumina sequencing platform.

The paired-end reads, demultiplexed based on barcode, were retrieved from the Illumina platform and joined by the software QIIME (version 1.9.1) (Caporaso et al, 2010). Reads with a quality score <20 were discarded by QIIME, and primer sequences were cut by Cutadapt (version 3.3) (Martin, 2011). Denoising (removing chimeric sequences, removing singletons, and dereplication) was done with USEARCH (version 11.0.667) (Edgar et al, 2011) and VSEARCH (version 2.15.0) (Rognes et al, 2016). Samples with read counts <3000 were discarded. The Amplicon Sequence Variants (ASVs) were assigned based on Ribosomal Database Project (RDP) set 16 with RDP classifier (Cole et al, 2014).

### Statistical analysis

Characteristics of the study population were shown with percentages, means [SD (standard deviation)], or median values with interquartile ranges (IQRs). Data of oGTT, reported as means ± SE (standard error), were analyzed by two-way repeated-measures ANOVA with time and group as factors. Differences between groups were tested with independent samples using the ANOVA test or the Kruskal–Wallis test for non-normally distributed continuous variables (or the Mann–Whitney *U* test in case of unfulfilled test assumptions). Within-group analyses were performed using the Kruskal–Wallis test or Mann–Whitney *U* test (as appropriate) due to different sample size, or Wilcoxon signed-rank test for the pairwise comparison. As an *ad hoc* analysis the Ribo50 and Ribo100 groups were also analyzed as combination, defined as the “RiboCom” group.

The 16S rRNA gene sequencing data were normalized using cumulative sum scaling for multivariate analysis. The calculation of alpha diversity was performed using QIIME. The beta diversity was calculated *via* different distances, represented in principal coordinate analysis (PCoA) and performed using R package “phyloseq” (version 1.34.0) and the ADONIS function in the “vegan” package to test significant between groups with 999 permutations. Linear regression and Pearson's or Spearman's correlation analysis was performed using R package “base,” “psych,” (version 2.1.3) and “corrplot” (version 0.87).

Molecular Ecological Networks (MENs) were constructed on the basis of Pearson's correlations of log-transformed ASV abundances, followed by a random matrix theory-based approach that calculated the correlation cutoff threshold in an automatic way (Carr et al, 2019; Goberna et al, 2019; Zhou et al, 2010). The methods and statistical tools were applied using the settings indicated by the Molecular Ecological Network Analysis Pipeline, which is open–accessible now (http://ieg2.ou.edu/MENA) (Deng et al, 2012). Molecular Complex Detection (MCODE) algorithm (Bader and Hogue, 2003) in Cytoscape (version 3.9.0) was used for network clustering by default settings.

The visualization and statistical analysis were performed using the software R (version 4.0.2) for Windows 10. *p* Values <0.05 were considered as statistically significant. *p*-values were adjusted for multiple testing by using the Benjamini–Hochberg method.

## Supplementary Material

Supplemental data

Supplemental data

Supplemental data

Supplemental data

Supplemental data

Supplemental data

Supplemental data

Supplemental data

Supplemental data

Supplemental data

Supplemental data

## Data Availability

The raw sequencing data have been submitted to the National Center for Biotechnology Information (NCBI) Sequence Read Archive (SRA) under accession number BioProject PRJNA791950. All data generated or analyzed during this study are included in this article (and its supplementary files) or through the SRA, and are thus freely accessible.

## Abbreviations Used

ASV: Amplicon Sequence Variant
BMI: body mass index
CCK: cholecystokinin
CD: Crohn's disease
eCRF: electronic case report form
FISH: fluorescence *in situ* hybridization
GC-MS: gas chromatography-mass spectrometry
GLP-1: glucagon-like peptide-1
GLP-2: glucagon-like peptide-2
IBD: inflammatory bowel diseases
IQR: interquartile ranges
MCODE: molecular complex detection
MENs: molecular ecological networks
NCBI: National Center for Biotechnology Information
oGTT: oral glucose tolerance test
PCoA: principal coordinate analysis
PYY: peptide YY
RDP: ribosomal database project
RMT: random matrix theory
SCFA: short-chain fatty acids
SD: standard deviation
SE: standard error
SRA: sequence read archive
UMCG: University Medical Center Groningen
VAS: visual analog scale
