# Supplementary material for: Riboflavin Supplementation Promotes Butyrate Production in the Absence of Gross Compositional Changes in the Gut Microbiota
Source: Antioxid Redox Signal. 2023 Feb 14;38(4):282–97. doi: 10.1089/ars.2022.0033 (PMC9986023; doi:10.1089/ars.2022.0033)
Supplement: Supplemental data [file Suppl_FigS1.docx]

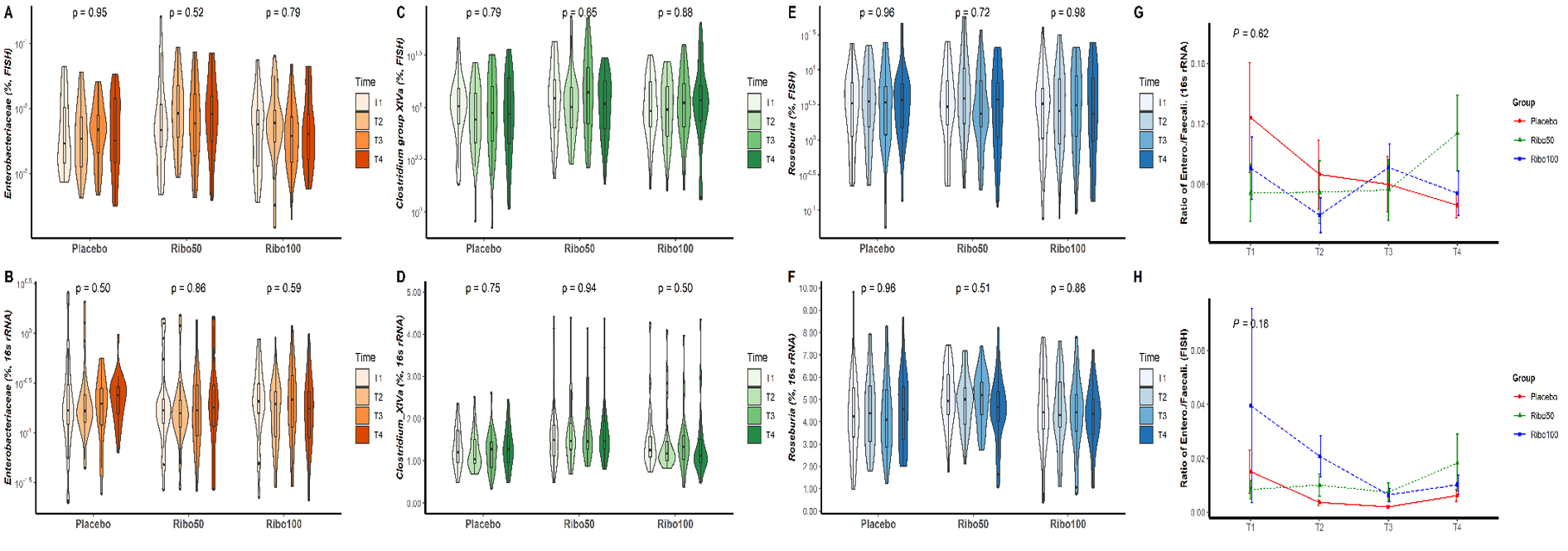
 **Supplementary Figure 1.** The content of *Enterobacteriaceae,* *Clostridium* group XlVa and *Roseburia* and the ratio between *Enterobacteriaceae* and *Faecalibacterium* from 16S rRNA gene sequencing (A, C, E, H) and FISH (B, D, F, G). **A-F**, the content of *Enterobacteriaceae* (A, B), *Clostridium* group XlVa (C, D) and *Roseburia* (E, F) were not changed over all time points among three groups (*p* > 0.05, Kruskal-Wallis test). **G-H**, the ratio of *Enterobacteriaceae /Faecalibacterium* didn’t change significantly over all time points among three groups (*p* = 0.62, *p* = 0.18, respectively; Kruskal-Wallis test).
