# Supplementary material for: Riboflavin Supplementation Promotes Butyrate Production in the Absence of Gross Compositional Changes in the Gut Microbiota
Source: Antioxid Redox Signal. 2023 Feb 14;38(4):282–97. doi: 10.1089/ars.2022.0033 (PMC9986023; doi:10.1089/ars.2022.0033)
Supplement: Supplemental data [file Suppl_TableS1.docx]

**Supplementary Table 1**. Participant and sample characteristics, and FISH data.

| **SampleID** | **Subject** | | **Time** | **Reads count** | **Group** | **Group2** | **Gender** | **height (cm)** | **weight (kg)** | **BMI** | **Riboflavin concertration of faeces (ng/mg)** | **DW (dry weight of faeces, %)** | **Butyrate (mM)** | **Propionate (mM)** | **Acetate (mM)** | ***F. prausnitzii* (Cell/g faeces wetweight)** | **per g faeces (Counts/g faeces wetweight)** | | | |
| --- | --- | --- | --- | --- | --- | --- | --- | --- | --- | --- | --- | --- | --- | --- | --- | --- | --- | --- | --- | --- |
|  |  |  |  |  |  |  |  |  |  |  |  |  |  |  |  |  | **all bacteria** | ***Clostridium* group** | ***Roseburia* species** | ***Enterobacteriaceae*** |
| r1.24 | S002 | | T1 | 87597 | Ribo100 | RiboCom | 2 | 186 | 83 | 24 | 755 | 19.74 | 5.1 | 6.2 | 25.5 | 914395363 | 1.509E+11 | 9.918E+09 | 1.7E+09 | 1E+06 |
| r1.25 | S002 | | T2 | 62960 | Ribo100 | RiboCom | 2 | 186 | 83 | 24 | 1050 | 30.97 | 7 | 11.5 | 44 | 1150368360 | 1.836E+11 | 1.313E+10 | 1.08E+09 | 1E+06 |
| r1.26 | S002 | | T3 | 88761 | Ribo100 | RiboCom | 2 | 186 | 83 | 24 | 5570 | 31.25 | 9.8 | 10.8 | 61.8 | 2.3701E+10 | 1.88E+11 | 7.042E+09 | 9E+08 | 368708 |
| r1.27 | S002 | | T4 | 73549 | Ribo100 | RiboCom | 2 | 186 | 83 | 24 | 2740 | 32.09 | 5.7 | 9.3 | 35.1 | 235972997 | 1.329E+11 | 6.895E+09 | 2.06E+08 | 2E+06 |
| r1.28 | S004 | | T1 | 69576 | Ribo100 | RiboCom | 2 | 174 | 76 | 25 | 1830 | 20.4 | 16.6 | 30.1 | 71.9 | 4394997067 | 4.926E+10 | 5.531E+09 | 1.76E+09 | 737416 |
| r1.29 | S004 | | T2 | 93810 | Ribo100 | RiboCom | 2 | 174 | 76 | 25 | 2160 | 30.14 | 28.4 | 30.7 | 74.7 | 1.0737E+10 | 1.131E+11 | 1.058E+10 | 1.02E+10 | 368708 |
| r1.30 | S004 | | T3 | 80808 | Ribo100 | RiboCom | 2 | 174 | 76 | 25 | 4980 | 27.96 | 23.7 | 17.6 | 51.2 | 6695733786 | 5.545E+10 | 3.613E+09 | 9.1E+09 | 737416 |
| r1.31 | S004 | | T4 | 59223 | Ribo100 | RiboCom | 2 | 174 | 76 | 25 | 2190 | 21.9 | 21 | 23.7 | 75.1 | 6194291168 | 3.702E+10 | 1.243E+10 | 2.32E+09 | 2E+06 |
| r1.32 | S016 | | T1 | 96363 | Ribo100 | RiboCom | 1 | 178 | 73 | 23 | 1000 | 14.91 | 19.4 | 14 | 65.4 | 2330233344 | 3.023E+10 | 5.125E+09 | 3.55E+09 | 737416 |
| r1.33 | S016 | | T2 | 65604 | Ribo100 | RiboCom | 1 | 178 | 73 | 23 | 1550 | 15.98 | 15.2 | 9.2 | 46.3 | 1563321104 | 5.088E+10 | 5.309E+09 | 4.63E+09 | 737416 |
| r1.34 | S016 | | T3 | 72973 | Ribo100 | RiboCom | 1 | 178 | 73 | 23 | 8200 | 21.42 | 21.2 | 12.1 | 46.1 | 1342096420 | 5.147E+10 | 1.486E+10 | 4.14E+09 | 737416 |
| r1.35 | S016 | | T4 | 104845 | Ribo100 | RiboCom | 1 | 178 | 73 | 23 | 2130 | 23.05 | 16.7 | 14.1 | 51.9 | 1401089669 | 9.631E+10 | 2.555E+10 | 3.82E+09 | 7E+06 |
| r1.36 | S017 | | T1 | 77575 | Ribo50 | RiboCom | 1 | 183 | 61 | 18 | 1390 | 29.24 | 12.7 | 13.8 | 41.3 | 353959495 | 5.044E+10 | 1.549E+09 | 2.36E+08 | 369000 |
| r1.37 | S017 | | T2 | 89792 | Ribo50 | RiboCom | 1 | 183 | 61 | 18 | 1770 | 26.73 | 14.3 | 20.1 | 51.1 | 2123756972 | 6.165E+10 | 1.77E+09 | 1.53E+10 | 2E+06 |
| r1.38 | S017 | | T3 | 87085 | Ribo50 | RiboCom | 1 | 183 | 61 | 18 | 2140 | 29.32 | 3.8 | 8.1 | 27 | 73741562 | 8.112E+10 | 3.392E+09 | 1.21E+10 | 368708 |
| r1.39 | S017 | | T4 | 83617 | Ribo50 | RiboCom | 1 | 183 | 61 | 18 | 1310 | 35.63 | 2.6 | 6.2 | 19.4 | 973388612 | 5.206E+10 | 2.212E+09 | 3.98E+08 | 3E+06 |
| r1.40 | S020 | | T1 | 62909 | Ribo50 | RiboCom | 1 | 166 | 59 | 21 | 960 | 26.57 | 12.8 | 9.3 | 49 | 132734811 | 1.534E+10 | 2.323E+09 | 1.58E+09 | 1E+07 |
| r1.41 | S020 | | T2 | 68021 | Ribo50 | RiboCom | 1 | 166 | 59 | 21 | 862 | 26.54 | 19.9 | 9 | 35.3 | 1401089669 | 1.268E+10 | 1.88E+09 | 4.57E+08 | 3E+06 |
| r1.42 | S020 | | T3 | 65636 | Ribo50 | RiboCom | 1 | 166 | 59 | 21 | 1470 | 28.89 | 22.8 | 14 | 57.3 | 884898738 | 2.846E+10 | 2.065E+09 | 7.23E+08 | 2E+06 |
| r1.43 | S020 | | T4 | 86322 | Ribo50 | RiboCom | 1 | 166 | 59 | 21 | 2610 | 28.9 | 9.5 | 7.7 | 32.3 | 1253606546 | 2.168E+10 | 1.106E+09 | 9.14E+08 | 368708 |
| r1.45 | S024 | | T2 | 54993 | Placebo | Placebo | 2 | 164 | 53 | 20 | 2290 | 22.92 | 20 | 10.8 | 57.2 | 1106123423 | 3.805E+10 | 8.407E+09 | 4.01E+09 | 368708 |
| r1.46 | S024 | | T3 | 47103 | Placebo | Placebo | 2 | 164 | 53 | 20 | 1130 | 28.63 | 18.1 | 16.4 | 47.7 | 309714558 | 6.386E+10 | 1.302E+10 | 6.53E+09 | 737416 |
| r1.48 | S024 | | T4 | 57375 | Placebo | Placebo | 2 | 164 | 53 | 20 | 2360 | 30.1 | 22.2 | 15.2 | 49.8 | 707918991 | 8.657E+10 | 1.973E+10 | 5.71E+09 | 1E+06 |
| r2.1 | S001 | | T1 | 106638 | Ribo50 | RiboCom | 2 | 168 | 60 | 21 | 1210 | 27.89 | 7.9 | 6.3 | 33 | 575184180 | 8.407E+09 | 516190931 | 3.69E+08 | 369000 |
| r2.10 | S007 | | T2 | 82003 | Ribo100 | RiboCom | 2 | 161 | 49 | 19 | 1470 | 29.77 | 10.7 | 11.9 | 32.1 | 1860000000 | 2.63E+10 | 1.36E+09 | 8.23E+09 | 2E+06 |
| r2.11 | S007 | | T3 | 101518 | Ribo100 | RiboCom | 2 | 161 | 49 | 19 | 5340 | 32.37 | 17.4 | 15.4 | 41.5 | 796000000 | 2.96E+10 | 1.92E+09 | 1.62E+08 | 2E+06 |
| r2.12 | S007 | | T4 | 47561 | Ribo100 | RiboCom | 2 | 161 | 49 | 19 | 965 | 34.69 | 14.4 | 14 | 26.1 | 944000000 | 1.21E+10 | 1.4E+09 | 2.94E+09 | 3E+06 |
| r2.13 | S008 | | T1 | 102352 | Ribo50 | RiboCom | 2 | 172 | 67 | 23 | 1270 | 31.26 | 11.5 | 12.9 | 59 | 206000000 | 1.06E+11 | 1.29E+10 | 9E+08 | 8E+06 |
| r2.14 | S008 | | T2 | 93711 | Ribo50 | RiboCom | 2 | 172 | 67 | 23 | 966 | 37.51 | 7.3 | 7.4 | 24.2 | 29500000 | 8.88E+10 | 1.06E+10 | 5.6E+08 | 3E+06 |
| r2.15 | S008 | | T3 | 81814 | Ribo50 | RiboCom | 2 | 172 | 67 | 23 | 2710 | 38.09 | 25.9 | 13 | 39.9 | 2760000000 | 5.31E+10 | 2.54E+09 | 3.39E+08 | 9E+06 |
| r2.16 | S008 | | T4 | 77572 | Ribo50 | RiboCom | 2 | 172 | 67 | 23 | 518 | 32.29 | 3.2 | 4.1 | 16.1 | 8480000000 | 2.18E+10 | 2.03E+09 | 3.54E+08 | 3E+06 |
| r2.17 | S009 | | T1 | 89112 | Ribo50 | RiboCom | 1 | 185 | 74 | 22 | 1680 | 25.04 | 9.6 | 14.3 | 48.3 | 1330000000 | 3.51E+10 | 2.51E+09 | 3.39E+08 | 3E+06 |
| r2.18 | S009 | | T2 | 60181 | Ribo50 | RiboCom | 1 | 185 | 74 | 22 | 1150 | 35.08 | 7.3 | 10.8 | 26.6 | 73700000 | 7.15E+10 | 4.54E+09 | 1.47E+08 | 3E+06 |
| r2.19 | S009 | | T3 | 93541 | Ribo50 | RiboCom | 1 | 185 | 74 | 22 | 5980 | 28.42 | 7 | 12.3 | 32 | 4010000000 | 5.01E+10 | 6.97E+09 | 9.88E+08 | 2E+06 |
| r2.2 | S001 | | T2 | 105309 | Ribo50 | RiboCom | 2 | 168 | 60 | 21 | 748 | 25.39 | 9.1 | 7.6 | 41.7 | 88489873.8 | 8.849E+09 | 294966246 | 29496625 | 369000 |
| r2.20 | S009 | | T4 | 56729 | Ribo50 | RiboCom | 1 | 185 | 74 | 22 | 592 | 27.17 | 8.3 | 13.9 | 28.2 | 4420000000 | 2.43E+10 | 4.83E+09 | 2.65E+08 | 2E+07 |
| r2.21 | S025 | | T1 | 57863 | Placebo | Placebo | 2 | 167 | 61 | 22 | 2190 | 30.07 | 24.9 | 23.4 | 56.5 | 2507213092 | 2.139E+10 | 2.175E+09 | 1.5E+09 | 5E+06 |
| r2.22 | S025 | | T2 | 70712 | Placebo | Placebo | 2 | 167 | 61 | 22 | 2770 | 29.58 | 10.8 | 10.6 | 30.5 | 1253606546 | 1.608E+10 | 1.844E+09 | 2.64E+09 | 5E+06 |
| r2.23 | S025 | | T3 | 94928 | Placebo | Placebo | 2 | 167 | 61 | 22 | 2300 | 29.58 | 27.8 | 20.1 | 67 | 1991022161 | 1.814E+10 | 1.475E+09 | 1.77E+09 | 3E+06 |
| r2.24 | S025 | | T4 | 80778 | Placebo | Placebo | 2 | 167 | 61 | 22 | 2160 | 29.92 | 28.7 | 15.8 | 54.8 | 1696055915 | 2.714E+10 | 1.254E+09 | 2.06E+09 | 2E+06 |
| r2.25 | S026 | | T1 | 53071 | Placebo | Placebo | 2 | 166 | 67 | 24 | 1400 | 19.14 | 5.8 | 9.8 | 39.9 | 4440000000 | 2.526E+11 | 1.088E+10 | 6.02E+09 | 2E+06 |
| r2.26 | S026 | | T2 | 67287 | Placebo | Placebo | 2 | 166 | 67 | 24 | 7150 | 20.92 | 2 | 3.2 | 15.6 | 1386341357 | 8.303E+10 | 5.162E+09 | 1.82E+10 | 2E+06 |
| r2.27 | S026 | | T3 | 65024 | Placebo | Placebo | 2 | 166 | 67 | 24 | 3590 | 34.33 | 6.3 | 7.2 | 31.9 | 8391789701 | 6.106E+10 | 1.434E+10 | 1.48E+10 | 2E+06 |
| r2.28 | S026 | | T4 | 62936 | Placebo | Placebo | 2 | 166 | 67 | 24 | 1470 | 33.62 | 12.6 | 16.5 | 58.4 | 1297851483 | 3.451E+10 | 1.231E+10 | 9.91E+09 | 3E+06 |
| r2.29 | S028 | | T1 | 76851 | Placebo | Placebo | 1 | 189 | 77 | 22 | 3900 | 25.1 | 8.6 | 7.5 | 36.8 | 486694306 | 4.867E+10 | 5.309E+09 | 2.05E+09 | 737416 |
| r2.3 | S001 | | T3 | 96999 | Ribo50 | RiboCom | 2 | 168 | 60 | 21 | 4340 | 30.02 | 14.2 | 11.2 | 46.2 | 471945994 | 6.047E+09 | 258095466 | 14700000 | 369000 |
| r2.30 | S028 | | T2 | 88088 | Placebo | Placebo | 1 | 189 | 77 | 22 | 2630 | 33.66 | 2.9 | 3.4 | 7.9 | 471945994 | 3.613E+10 | 1.659E+09 | 6.64E+08 | 3E+06 |
| r2.31 | S028 | | T3 | 62285 | Placebo | Placebo | 1 | 189 | 77 | 22 | 2330 | 19.4 | 15 | 24.7 | 72.7 | 929143675 | 1.858E+10 | 848027958 | 2.95E+08 | 5E+06 |
| r2.32 | S028 | | T4 | 87416 | Placebo | Placebo | 1 | 189 | 77 | 22 | 3280 | 37.81 | 9.4 | 9.6 | 33.9 | 2.5264E+10 | 7.905E+10 | 1.497E+10 | 1.55E+09 | 369000 |
| r2.33 | S029 | | T1 | 69639 | Ribo50 | RiboCom | 2 | 168 | 66 | 24 | 1590 | 30.18 | 20.2 | 13.9 | 52.4 | 1533824480 | 6.268E+10 | 1.604E+10 | 2.48E+09 | 2E+06 |
| r2.34 | S029 | | T2 | 61974 | Ribo50 | RiboCom | 2 | 168 | 66 | 24 | 1760 | 31.37 | 9.4 | 11.7 | 37.8 | 2241743470 | 7.684E+10 | 9.07E+09 | 2.88E+09 | 2E+06 |
| r2.35 | S029 | | T3 | 64098 | Ribo50 | RiboCom | 2 | 168 | 66 | 24 | 4600 | 33.07 | 19.2 | 14.2 | 39.5 | 1946777224 | 5.737E+10 | 1.368E+10 | 3.76E+09 | 1E+06 |
| r2.36 | S029 | | T4 | 65323 | Ribo50 | RiboCom | 2 | 168 | 66 | 24 | 1240 | 32.14 | 34.5 | 14.6 | 86.5 | 5294644117 | 9.586E+10 | 1.81E+10 | 7.39E+09 | 368708 |
| r2.37 | S031 | | T1 | 86651 | Placebo | Placebo | 2 | 159 | 58 | 23 | 725 | 23.08 | 14.6 | 9.7 | 65.7 | 516190931 | 3.599E+10 | 3.724E+09 | 1.28E+09 | 4E+06 |
| r2.38 | S031 | | T2 | 95107 | Placebo | Placebo | 2 | 159 | 58 | 23 | 1060 | 32.81 | 24.5 | 17.5 | 77.9 | 427701057 | 9.778E+10 | 2.876E+09 | 1.55E+09 | 1E+06 |
| r2.39 | S031 | | T3 | 64109 | Placebo | Placebo | 2 | 159 | 58 | 23 | 947 | 27.08 | 4.5 | 6.7 | 30.7 | 973388612 | 5.501E+10 | 1.807E+09 | 5.16E+08 | 3E+06 |
| r2.4 | S001 | | T4 | 94051 | Ribo50 | RiboCom | 2 | 168 | 60 | 21 | 752 | 31.26 | 7.7 | 7.3 | 35.5 | 117986499 | 6.047E+09 | 110612343 | 14700000 | 369000 |
| r2.40 | S031 | | T4 | 83890 | Placebo | Placebo | 2 | 159 | 58 | 23 | 512 | 30.6 | 2.5 | 3.9 | 22.5 | 988136924 | 4.749E+10 | 1.327E+09 | 6.78E+08 | 8E+06 |
| r2.41 | S033 | | T1 | 81069 | Ribo100 | RiboCom | 1 | 178 | 69 | 22 | 2730 | 27.87 | 5.1 | 6.5 | 19.5 | 2875920899 | 1.726E+10 | 3.281E+09 | 7.37E+08 | 737416 |
| r2.42 | S033 | | T2 | 75377 | Ribo100 | RiboCom | 1 | 178 | 69 | 22 | 1570 | 33.14 | 10.6 | 10.2 | 32 | 2787431026 | 3.687E+10 | 7.559E+09 | 1.76E+09 | 737416 |
| r2.43 | S033 | | T3 | 73392 | Ribo100 | RiboCom | 1 | 178 | 69 | 22 | 1860 | 25.48 | 13.5 | 9.9 | 47.8 | 4763704874 | 3.023E+10 | 7.853E+09 | 9.14E+08 | 2E+06 |
| r2.44 | S033 | | T4 | 183026 | Ribo100 | RiboCom | 1 | 178 | 69 | 22 | 2290 | 21.83 | 16.5 | 12.9 | 66.7 | 2698941152 | 3.289E+10 | 1.11E+10 | 7.52E+08 | 737416 |
| r2.45 | S036 | | T1 | 99426 | Placebo | Placebo | 1 | 179 | 73 | 23 | 1200 | 27.39 | 9.1 | 10.1 | 45.8 | 737415615 | 5.693E+10 | 3.318E+09 | 5.75E+08 | 369000 |
| r2.46 | S036 | | T2 | 122519 | Placebo | Placebo | 1 | 179 | 73 | 23 | 1630 | 33.64 | 8.2 | 9.9 | 32.4 | 914395363 | 8.805E+10 | 2.839E+09 | 5.46E+08 | 368708 |
| r2.47 | S036 | | T3 | 138511 | Placebo | Placebo | 1 | 179 | 73 | 23 | 1440 | 36.16 | 3 | 5 | 17.4 | 2020518786 | 6.843E+10 | 2.47E+09 | 5.16E+08 | 737416 |
| r2.48 | S036 | | T4 | 89149 | Placebo | Placebo | 1 | 179 | 73 | 23 | 1250 | 29.16 | 7.4 | 8.1 | 29.5 | 353959495 | 5.014E+10 | 4.461E+09 | 1.93E+09 | 737416 |
| r2.49 | S039 | | T1 | 98798 | Ribo50 | RiboCom | 2 | 180 | 79 | 24 | 2200 | 28.46 | 18.4 | 17.5 | 49.6 | 1799294101 | 8.259E+10 | 1.563E+10 | 2.02E+09 | 2E+06 |
| r2.5 | S006 | | T1 | 96417 | Placebo | Placebo | 2 | 152 | 53 | 23 | 506 | 28.55 | 5 | 4.9 | 20.2 | 147483123 | 2.802E+09 | 73741562 | 88489874 | 369000 |
| r2.51 | S039 | | T3 | 84872 | Ribo50 | RiboCom | 2 | 180 | 79 | 24 | 4860 | 21.5 | 10.9 | 13.8 | 54 | 280217934 | 6.371E+10 | 2.279E+10 | 1.01E+10 | 2E+06 |
| r2.52 | S039 | | T4 | 90656 | Ribo50 | RiboCom | 2 | 180 | 79 | 24 | 1180 | 24.36 | 14.1 | 14.6 | 46.9 | 1179864984 | 1.24E+11 | 1.331E+10 | 8.05E+09 | 4E+06 |
| r2.53 | S043 | | T1 | 91089 | Ribo50 | RiboCom | 1 | 183 | 82 | 25 | 1280 | 27.84 | 3.9 | 4.6 | 13 | 2477716467 | 1.947E+10 | 4.572E+09 | 5.46E+08 | 3E+06 |
| r2.54 | S043 | | T2 | 66128 | Ribo50 | RiboCom | 1 | 183 | 82 | 25 | 1590 | 15.96 | 7.1 | 6.5 | 18.8 | 3244628707 | 1.755E+10 | 2.95E+09 | 6.78E+08 | 2E+06 |
| r2.55 | S043 | | T3 | 67435 | Ribo50 | RiboCom | 1 | 183 | 82 | 25 | 3840 | 38.3 | 14.9 | 13.6 | 31.5 | 5250399180 | 1.637E+10 | 6.747E+09 | 1.46E+09 | 1E+06 |
| r2.56 | S043 | | T4 | 120516 | Ribo50 | RiboCom | 1 | 183 | 82 | 25 | 1250 | 31.08 | 4.7 | 5.4 | 17.1 | 2433471530 | 8.849E+09 | 1.364E+09 | 5.75E+08 | 1E+06 |
| r2.57 | S044 | | T1 | 88958 | Ribo100 | RiboCom | 2 | 168 | 60 | 21 | 757 | 27.9 | 10.5 | 10.8 | 35.3 | 9586402998 | 3.849E+10 | 3.208E+09 | 8.26E+08 | 369000 |
| r2.58 | S044 | | T2 | 90994 | Ribo100 | RiboCom | 2 | 168 | 60 | 21 | 1000 | 12.95 | 14.7 | 14.8 | 50.2 | 427701057 | 9.985E+10 | 3.023E+09 | 3.39E+08 | 7E+06 |
| r2.59 | S044 | | T3 | 87478 | Ribo100 | RiboCom | 2 | 168 | 60 | 21 | 2430 | 29.85 | 13.4 | 9.9 | 25.8 | 4424493691 | 6.843E+10 | 3.318E+09 | 3.02E+09 | 2E+06 |
| r2.6 | S006 | | T2 | 101670 | Placebo | Placebo | 2 | 152 | 53 | 23 | 622 | 31.16 | 4.1 | 4.7 | 19.1 | 147483123 | 2.802E+09 | 184353904 | 29496625 | 369000 |
| r2.60 | S044 | | T4 | 108774 | Ribo100 | RiboCom | 2 | 168 | 60 | 21 | 828 | 21.6 | 15.4 | 16.1 | 44.1 | 5619106988 | 5.59E+10 | 9.771E+09 | 8.85E+08 | 2E+06 |
| r2.61 | S049 | | T1 | 95485 | Ribo100 | RiboCom | 1 | 178 | 72 | 23 | 562 | 20.86 | 4.6 | 5.9 | 19 | 1533824480 | 5.826E+10 | 958640300 | 1.17E+09 | 737416 |
| r2.62 | S049 | | T2 | 82573 | Ribo100 | RiboCom | 1 | 178 | 72 | 23 | 707 | 16.45 | 3.5 | 5.8 | 19.5 | 973388612 | 3.318E+10 | 6.858E+09 | 7.23E+08 | 5E+06 |
| r2.63 | S049 | | T3 | 93756 | Ribo100 | RiboCom | 1 | 178 | 72 | 23 | 203000 | 25.47 | 13.1 | 11.7 | 47.8 | 339211183 | 2.876E+10 | 3.171E+09 | 3.24E+08 | 1E+06 |
| r2.64 | S049 | | T4 | 80353 | Ribo100 | RiboCom | 1 | 178 | 72 | 23 | 479 | 21.89 | 4.9 | 6 | 20.1 | 589932492 | 1.342E+10 | 921769519 | 1.34E+09 | 6E+06 |
| r2.65 | S068 | | T1 | 84726 | Placebo | Placebo | 1 | 195 | 81 | 21 | 2220 | 26.24 | 10.7 | 11.7 | 51.8 | 1725552540 | 6.784E+09 | 1.217E+09 | 1.59E+09 | 3E+06 |
| r2.66 | S068 | | T2 | 86523 | Placebo | Placebo | 1 | 195 | 81 | 21 | 2000 | 34.18 | 16.1 | 16.2 | 41.1 | 1017633549 | 1.003E+10 | 1.364E+09 | 4.28E+08 | 737416 |
| r2.67 | S068 | | T3 | 96817 | Placebo | Placebo | 1 | 195 | 81 | 21 | 997 | 30.1 | 8.3 | 9 | 30.6 | 442449369 | 1.106E+10 | 848027958 | 8.11E+08 | 368708 |
| r2.68 | S068 | | T4 | 88522 | Placebo | Placebo | 1 | 195 | 81 | 21 | 1080 | 26.46 | 9.5 | 10.6 | 36.3 | 1076626798 | 1.652E+10 | 516190931 | 7.08E+08 | 3E+06 |
| r2.69 | S081 | | T1 | 86945 | Ribo50 | RiboCom | 2 | 163 | 61 | 23 | 922 | 22.02 | 20.9 | 11.8 | 30.5 | 2241743470 | 4.174E+10 | 2.028E+09 | 9.73E+08 | 1E+06 |
| r2.7 | S006 | | T3 | 69166 | Placebo | Placebo | 2 | 152 | 53 | 23 | 628 | 29.29 | 3.2 | 4 | 17.9 | 294966246 | 3.982E+09 | 36900000 | 14700000 | 369000 |
| r2.70 | S081 | | T2 | 91309 | Ribo50 | RiboCom | 2 | 163 | 61 | 23 | 620 | 22.87 | 9.2 | 5.5 | 26.4 | 1828790726 | 1.681E+10 | 1.659E+09 | 8.97E+09 | 1E+06 |
| r2.71 | S081 | | T3 | 80988 | Ribo50 | RiboCom | 2 | 163 | 61 | 23 | 3660 | 22.87 | 18.4 | 8.5 | 38.4 | 6990700032 | 1.844E+10 | 2.95E+09 | 4.28E+08 | 737416 |
| r2.72 | S081 | | T4 | 104117 | Ribo50 | RiboCom | 2 | 163 | 61 | 23 | 995 | 24.47 | 18.1 | 12.1 | 26.7 | 1843539038 | 3.938E+10 | 4.314E+09 | 44244937 | 1E+06 |
| r2.73 | S087 | | T1 | 101112 | Ribo50 | RiboCom | 2 | 175 | 71 | 23 | 1130 | 31.62 | 9.9 | 8.2 | 32.1 | 44244937 | 3.377E+10 | 1.217E+09 | 73741562 | 2E+06 |
| r2.74 | S087 | | T2 | 90606 | Ribo50 | RiboCom | 2 | 175 | 71 | 23 | 1100 | 13.99 | 9.3 | 9.9 | 35.3 | 530939243 | 1.755E+10 | 1.954E+09 | 2.02E+09 | 1E+06 |
| r2.75 | S087 | | T3 | 99382 | Ribo50 | RiboCom | 2 | 175 | 71 | 23 | 1740 | 34.16 | 18.4 | 20.7 | 72.6 | 103238186 | 1.165E+10 | 1.069E+09 | 1.18E+08 | 2E+06 |
| r2.76 | S087 | | T4 | 86220 | Ribo50 | RiboCom | 2 | 175 | 71 | 23 | 1150 | 22.78 | 8.8 | 8.2 | 28.8 | 1135620048 | 2.05E+10 | 1.549E+09 | 3.76E+09 | 1E+06 |
| r2.77 | S091 | | T1 | 93207 | Ribo100 | RiboCom | 2 | 170 | 57 | 20 | 972 | 20.72 | 3.4 | 4.7 | 26.9 | 486694306 | 4.941E+10 | 1.512E+09 | 2.51E+08 | 737416 |
| r2.78 | S091 | | T2 | 83988 | Ribo100 | RiboCom | 2 | 170 | 57 | 20 | 768 | 18.25 | 1.2 | 2.5 | 14.5 | 412952744 | 6.755E+10 | 1.069E+09 | 1.84E+09 | 2E+06 |
| r2.79 | S091 | | T3 | 90647 | Ribo100 | RiboCom | 2 | 170 | 57 | 20 | 2040 | 24.12 | 1 | 2 | 12.2 | 88489873.8 | 5E+10 | 811157177 | 44244937 | 4E+06 |
| r2.8 | S006 | | T4 | 106681 | Placebo | Placebo | 2 | 152 | 53 | 23 | 587 | 37.63 | 5.4 | 5.3 | 16.9 | 162231435 | 9.881E+09 | 479320150 | 3.54E+08 | 369000 |
| r2.80 | S091 | | T4 | 102705 | Ribo100 | RiboCom | 2 | 170 | 57 | 20 | 1200 | 25.25 | 1.4 | 2.5 | 12.9 | 3613336515 | 3.112E+10 | 4.904E+09 | 6.24E+09 | 369000 |
| r2.81 | S093 | | T1 | 97988 | Placebo | Placebo | 2 | 163 | 53 | 20 | 2400 | 21.41 | 16.2 | 20.4 | 76.9 | 988136924 | 4.97E+10 | 6.747E+09 | 4.26E+09 | 3E+06 |
| r2.82 | S093 | | T2 | 92608 | Placebo | Placebo | 2 | 163 | 53 | 20 | 696 | 18.77 | 33.2 | 9.7 | 100.9 | 1253606546 | 2.419E+10 | 4.24E+09 | 2.57E+09 | 368708 |
| r2.83 | S093 | | T3 | 80226 | Placebo | Placebo | 2 | 163 | 53 | 20 | 1980 | 19.91 | 15.3 | 12.6 | 51.2 | 324462871 | 1.094E+11 | 1.571E+10 | 1.3E+09 | 1E+06 |
| r2.84 | S093 | | T4 | 43976 | Placebo | Placebo | 2 | 163 | 53 | 20 | 2720 | 26.54 | 26 | 26.1 | 99.6 | 4144275758 | 1.168E+11 | 1.851E+10 | 2.3E+09 | 368708 |
| r2.85 | S095 | | T1 | 72039 | Ribo50 | RiboCom | 2 | 156 | 50 | 21 | 927 | 42.85 | 12.7 | 12.8 | 42 | 1.2698E+10 | 1.352E+11 | 7.559E+09 | 6.49E+08 | 1E+06 |
| r2.86 | S095 | | T2 | 125484 | Ribo50 | RiboCom | 2 | 156 | 50 | 21 | 1060 | 36.67 | 10.1 | 12 | 23.9 | 4114779133 | 3.776E+10 | 2.065E+09 | 6.78E+08 | 737416 |
| r2.87 | S095 | | T3 | 85957 | Ribo50 | RiboCom | 2 | 156 | 50 | 21 | 2100 | 36.81 | 9.9 | 16.4 | 52.7 | 14700000 | 9.07E+10 | 1.342E+10 | 1.77E+08 | 8E+06 |
| r2.88 | S095 | | T4 | 87248 | Ribo50 | RiboCom | 2 | 156 | 50 | 21 | 664 | 29.69 | 15.2 | 18.9 | 66.7 | 14748312 | 4.262E+10 | 7.669E+09 | 1.77E+08 | 3E+06 |
| r2.89 | S096 | | T1 | 47939 | Ribo100 | RiboCom | 2 | 170 | 59 | 20 | 1620 | 20.24 | 4.9 | 9.5 | 23.4 | 14748312.3 | 2.404E+10 | 2.065E+09 | 44244937 | 1E+07 |
| r2.9 | S007 | | T1 | 82880 | Ribo100 | RiboCom | 2 | 161 | 49 | 19 | 904 | 29.48 | 8.8 | 11.9 | 21.2 | 796000000 | 1.47E+10 | 1.33E+09 | 5.86E+09 | 2E+06 |
| r2.90 | S096 | | T2 | 73856 | Ribo100 | RiboCom | 2 | 170 | 59 | 20 | 1160 | 20.86 | 16.6 | 23.8 | 63.9 | 58993249.3 | 3.259E+10 | 5.125E+09 | 2.51E+08 | 7E+06 |
| r2.91 | S096 | | T3 | 76569 | Ribo100 | RiboCom | 2 | 170 | 59 | 20 | 347000 | 15.87 | 28.6 | 25.4 | 51.1 | 1.2212E+10 | 3.525E+10 | 1.014E+10 | 4.68E+09 | 1E+06 |
| r2.92 | S096 | | T4 | 47273 | Ribo100 | RiboCom | 2 | 170 | 59 | 20 | 994 | 27.66 | 9.4 | 17 | 40.7 | 176979748 | 2.758E+10 | 1.172E+10 | 5.4E+09 | 3E+06 |
| r2.93 | S097 | | T1 | 42793 | Placebo | Placebo | 2 | 157 | 60 | 24 | 2820 | 24.42 | 15.1 | 18.9 | 33 | 14748312 | 6.873E+10 | 1.254E+09 | 1.77E+08 | 737416 |
| r2.94 | S097 | | T2 | 102653 | Placebo | Placebo | 2 | 157 | 60 | 24 | 3960 | 22.38 | 15.8 | 15.5 | 33.9 | 1091375111 | 1.102E+11 | 9.844E+09 | 1.65E+09 | 6E+06 |
| r2.95 | S097 | | T3 | 73090 | Placebo | Placebo | 2 | 157 | 60 | 24 | 4650 | 16.22 | 13.9 | 23.7 | 65.5 | 2770000000 | 1.534E+11 | 3.355E+09 | 1.33E+09 | 3E+06 |
| r2.96 | S097 | | T4 | 67704 | Placebo | Placebo | 2 | 157 | 60 | 24 | 3970 | 25.73 | 14.4 | 18.9 | 48.5 | 1563321104 | 9.439E+10 | 6.416E+09 | 1.28E+09 | 368708 |
| r3.1 | S011 | | T1 | 78954 | Placebo | Placebo | 1 | 171 | 62 | 21 | 423 | 28.81 | 17.8 | 12.9 | 46.5 | 2650000000 | 2.12E+10 | 1.844E+09 | 2.43E+09 | 4E+06 |
| r3.10 | S014 | | T2 | 71101 | Placebo | Placebo | 2 | 156 | 52 | 22 | 733 | 29.55 | 10.9 | 13.5 | 47.4 | 442000000 | 3.78E+10 | 2.06E+09 | 88489874 | 5E+06 |
| r3.11 | S014 | | T3 | 80066 | Placebo | Placebo | 2 | 156 | 52 | 22 | 838 | 25.6 | 14.8 | 13.9 | 56.3 | 1780000000 | 2.36E+10 | 1.36E+09 | 1.33E+08 | 4E+06 |
| r3.12 | S014 | | T4 | 90361 | Placebo | Placebo | 2 | 156 | 52 | 22 | 846 | 31.92 | 11 | 11.4 | 42.6 | 516000000 | 2.32E+10 | 1.62E+09 | 3.39E+08 | 737000 |
| r3.13 | S018 | | T1 | 71632 | Ribo50 | RiboCom | 2 | 153 | 50 | 21 | 1270 | 40.06 | 13.6 | 12 | 28 | 4340000000 | 1.71E+10 | 1.7E+09 | 7.08E+08 | 1E+07 |
| r3.14 | S018 | | T2 | 75309 | Ribo50 | RiboCom | 2 | 153 | 50 | 21 | 1300 | 45.6 | 16.8 | 8.5 | 23.1 | 3200000000 | 1.42E+10 | 2.51E+09 | 9.59E+08 | 1E+07 |
| r3.15 | S018 | | T3 | 67326 | Ribo50 | RiboCom | 2 | 153 | 50 | 21 | 1670 | 43.77 | 11.3 | 5.7 | 23.2 | 472000000 | 1.73E+10 | 2.65E+09 | 58993249 | 8E+06 |
| r3.16 | S018 | | T4 | 70495 | Ribo50 | RiboCom | 2 | 153 | 50 | 21 | 1010 | 45.22 | 9.1 | 10.3 | 16.8 | 2850000000 | 1.05E+10 | 2.8E+09 | 3.98E+08 | 2E+06 |
| r3.17 | S019 | | T1 | 70904 | Ribo100 | RiboCom | 2 | 180 | 62 | 19 | 1920 | 21.56 | 11.7 | 18.9 | 52.7 | 221000000 | 3.2E+10 | 3.43E+09 | 1.92E+08 | 4E+06 |
| r3.18 | S019 | | T2 | 76517 | Ribo100 | RiboCom | 2 | 180 | 62 | 19 | 1660 | 14.12 | 16.9 | 24.2 | 67.7 | 2950000000 | 1.86E+10 | 1.51E+09 | 4E+09 | 1E+06 |
| r3.2 | S011 | | T2 | 82583 | Placebo | Placebo | 1 | 171 | 62 | 21 | 616 | 23.51 | 22.3 | 21.2 | 66 | 1330000000 | 1.47E+10 | 589932492 | 2.01E+09 | 3E+06 |
| r3.20 | S019 | | T4 | 59173 | Ribo100 | RiboCom | 2 | 180 | 62 | 19 | 1460 | 25.68 | 7.7 | 12.9 | 39.5 | 1270000000 | 2.98E+10 | 2.69E+09 | 58993249 | 7E+06 |
| r3.21 | S035 | | T1 | 84979 | Ribo50 | RiboCom | 2 | 164 | 62 | 23 | 3160 | 23.51 | 7.8 | 7.1 | 32 | 619429117 | 3.982E+09 | 1.696E+09 | 2.36E+08 | 368708 |
| r3.22 | S035 | | T2 | 85605 | Ribo50 | RiboCom | 2 | 164 | 62 | 23 | 2570 | 28.99 | 5.1 | 5.4 | 22.5 | 1622314354 | 9.586E+09 | 958640300 | 1E+09 | 3E+06 |
| r3.23 | S035 | | T3 | 86118 | Ribo50 | RiboCom | 2 | 164 | 62 | 23 | 3480 | 27.26 | 5.5 | 5.4 | 23.5 | 442449369 | 9.734E+09 | 516190931 | 6.19E+08 | 1E+06 |
| r3.24 | S035 | | T4 | 74776 | Ribo50 | RiboCom | 2 | 164 | 62 | 23 | 2300 | 28.04 | 5.4 | 4.8 | 21.9 | 575184180 | 6.637E+09 | 442449369 | 1.31E+09 | 2E+06 |
| r3.3 | S011 | | T3 | 81887 | Placebo | Placebo | 1 | 171 | 62 | 21 | 214 | 31.25 | 10.8 | 9.5 | 37.4 | 4350000000 | 1.83E+10 | 884898738 | 9.73E+08 | 3E+06 |
| r3.4 | S011 | | T4 | 84437 | Placebo | Placebo | 1 | 171 | 62 | 21 | 835 | 36.65 | 6.8 | 8.1 | 28 | 236000000 | 1.83E+10 | 516190931 | 9E+08 | 3E+06 |
| r3.5 | S012 | | T1 | 64429 | Placebo | Placebo | 2 | 163 | 61 | 23 | 3350 | 35.7 | 13.5 | 17.1 | 62 | 825905489 | 2.301E+10 | 2.065E+09 | 2.02E+09 | 368708 |
| r3.6 | S012 | | T2 | 75396 | Placebo | Placebo | 2 | 163 | 61 | 23 | 2020 | 28.63 | 12.7 | 13.6 | 52.5 | 589932492 | 1.49E+10 | 1.143E+09 | 6.64E+08 | 368708 |
| r3.7 | S012 | | T3 | 70126 | Placebo | Placebo | 2 | 163 | 61 | 23 | 2340 | 35.51 | 28 | 21.5 | 70.9 | 1873035663 | 3.333E+10 | 2.028E+09 | 4.23E+09 | 1E+06 |
| r3.8 | S012 | | T4 | 77520 | Placebo | Placebo | 2 | 163 | 61 | 23 | 2310 | 29.02 | 30.8 | 20.3 | 87.2 | 707918991 | 3.864E+10 | 2.323E+09 | 2.14E+09 | 1E+06 |
| r3.9 | S014 | | T1 | 77278 | Placebo | Placebo | 2 | 156 | 52 | 22 | 1280 | 24.22 | 11.5 | 16.1 | 60 | 3110000000 | 5.75E+10 | 8.96E+09 | 2.21E+08 | 1E+06 |
| r4.1 | S061 | | T1 | 86895 | Ribo50 | RiboCom | 2 | 167 | 52 | 19 | 2700 | 22.42 | 15.2 | 11.6 | 64.6 | 487000000 | 6.34E+09 | 848000000 | 1.77E+08 | 2E+06 |
| r4.10 | S064 | | T2 | 59329 | Ribo50 | RiboCom | 1 | 170 | 70 | 24 | 2160 | 16.66 | 20.5 | 22.3 | 66.7 | 1710000000 | 7.37E+09 | 1.18E+09 | 3.83E+08 | 369000 |
| r4.11 | S064 | | T3 | 61108 | Ribo50 | RiboCom | 1 | 170 | 70 | 24 | 17000 | 28.28 | 22.5 | 19.8 | 54.9 | 397000000 | 7.67E+09 | 1.62E+09 | 1.18E+08 | 1E+06 |
| r4.12 | S064 | | T4 | 60605 | Ribo50 | RiboCom | 1 | 170 | 70 | 24 | 2980 | 26.41 | 17.2 | 22.3 | 69.8 | 1590000000 | 4.87E+09 | 1.44E+09 | 4.42E+08 | 3E+06 |
| r4.13 | S066 | | T1 | 56223 | Placebo | Placebo | 2 | 169 | 58 | 20 | 2210 | 29.83 | 12 | 14.9 | 50.7 | 767000000 | 1.93E+10 | 2.18E+09 | 73741562 | 7E+06 |
| r4.14 | S066 | | T2 | 70804 | Placebo | Placebo | 2 | 169 | 58 | 20 | 2800 | 35.71 | 15.9 | 18 | 51.1 | 1710000000 | 2.23E+10 | 3.98E+09 | 4.28E+08 | 1E+06 |
| r4.15 | S066 | | T3 | 80667 | Placebo | Placebo | 2 | 169 | 58 | 20 | 2200 | 31.13 | 9.1 | 10.5 | 32.3 | 708000000 | 2.39E+10 | 7.63E+09 | 2.8E+08 | 2E+06 |
| r4.16 | S066 | | T4 | 61491 | Placebo | Placebo | 2 | 169 | 58 | 20 | 1670 | 30.22 | 8.3 | 9.1 | 33.6 | 162000000 | 1.71E+10 | 4.17E+09 | 14700000 | 3E+06 |
| r4.17 | S069 | | T1 | 85546 | Placebo | Placebo | 2 | 171 | 61 | 21 | 4460 | 27.96 | 5.7 | 11.5 | 31.8 | 1680000000 | 2.64E+10 | 4.17E+09 | 58993249 | 369000 |
| r4.18 | S069 | | T2 | 56669 | Placebo | Placebo | 2 | 171 | 61 | 21 | 2050 | 29.3 | 2 | 6.9 | 22.2 | 1090000000 | 2.21E+10 | 959000000 | 1.47E+08 | 1E+06 |
| r4.19 | S069 | | T3 | 60199 | Placebo | Placebo | 2 | 171 | 61 | 21 | 2210 | 19.01 | 11.3 | 12.1 | 54.7 | 2170000000 | 2.42E+10 | 3.13E+09 | 29496625 | 2E+06 |
| r4.2 | S061 | | T2 | 71667 | Ribo50 | RiboCom | 2 | 167 | 52 | 19 | 1790 | 27.16 | 7.3 | 5.7 | 23.5 | 442000000 | 5.6E+09 | 1.66E+09 | 8.55E+08 | 737000 |
| r4.20 | S069 | | T4 | 81535 | Placebo | Placebo | 2 | 171 | 61 | 21 | 3210 | 30.17 | NA | NA | NA | NA | NA | NA | NA | NA |
| r4.21 | S070 | | T1 | 66414 | Ribo100 | RiboCom | 2 | 161 | 48 | 19 | 4480 | 42.88 | 9.9 | 6.8 | 18.5 | 1780000000 | 5.31E+09 | 922000000 | 2.21E+08 | 369000 |
| r4.22 | S070 | | T2 | 70850 | Ribo100 | RiboCom | 2 | 161 | 48 | 19 | 2780 | 23.16 | 4.5 | 8 | 20.3 | 546000000 | 1.19E+10 | 1.81E+09 | 1.03E+08 | 737000 |
| r4.23 | S070 | | T3 | 55112 | Ribo100 | RiboCom | 2 | 161 | 48 | 19 | 22800 | 25.58 | 6 | 9.3 | 20.4 | 1180000000 | 1.47E+10 | 3.32E+09 | 4.57E+08 | 369000 |
| r4.24 | S070 | | T4 | 73817 | Ribo100 | RiboCom | 2 | 161 | 48 | 19 | 3210 | 23 | 6.5 | 13.6 | 33.3 | 14700000 | 1.43E+10 | 2.91E+09 | 1.18E+08 | 369000 |
| r4.25 | S038 | | T1 | 65590 | Ribo100 | RiboCom | 2 | 164 | 49 | 18 | 1790 | 19.05 | 12.3 | 16.1 | 50.6 | 412952745 | 7.522E+09 | 553061711 | 3.83E+08 | 369000 |
| r4.26 | S038 | | T2 | 72836 | Ribo100 | RiboCom | 2 | 164 | 49 | 18 | 1670 | 15.93 | 6.1 | 8.9 | 27.6 | 619429117 | 1.622E+09 | 442449369 | 1.47E+08 | 369000 |
| r4.27 | S038 | | T3 | 69651 | Ribo100 | RiboCom | 2 | 164 | 49 | 18 | 169000 | 11.52 | 11.9 | 19.2 | 68.1 | 104680804 | 2.95E+09 | 331837027 | 2.21E+08 | 368708 |
| r4.28 | S038 | | T4 | 48140 | Ribo100 | RiboCom | 2 | 164 | 49 | 18 | 1560 | 16.09 | 11.4 | 21 | 66.9 | 309714558 | 4.13E+09 | 258095465 | 2.36E+08 | 369000 |
| r4.29 | S056 | | T1 | 72479 | Ribo50 | RiboCom | 2 | 184 | 68 | 20 | 962 | 27.89 | 14.8 | 11.6 | 53 | 2300000000 | 3.41E+10 | 1.47E+09 | 1.03E+08 | 1E+06 |
| r4.3 | S061 | | T3 | 67490 | Ribo50 | RiboCom | 2 | 167 | 52 | 19 | 1980 | 19.54 | 13.8 | 20 | 60.1 | 501000000 | 8.85E+09 | 1.73E+09 | 2.36E+08 | 4E+06 |
| r4.30 | S056 | | T2 | 121603 | Ribo50 | RiboCom | 2 | 184 | 68 | 20 | 960 | 32.32 | 33.7 | 25.3 | 72.9 | 3600000000 | 2.49E+10 | 1.84E+10 | 4.28E+08 | 4E+06 |
| r4.31 | S056 | | T3 | 59098 | Ribo50 | RiboCom | 2 | 184 | 68 | 20 | 2470 | 32.03 | 7 | 13.6 | 37.2 | 1.16E+10 | 1.87E+10 | 8.11E+09 | 4.28E+08 | 1E+06 |
| r4.32 | S056 | | T4 | 77666 | Ribo50 | RiboCom | 2 | 184 | 68 | 20 | 796 | 23.77 | 20.5 | 22.5 | 66.2 | 2330000000 | 1.59E+10 | 2.47E+09 | 1.33E+08 | 2E+06 |
| r4.33 | S057 | | T1 | 68646 | Ribo100 | RiboCom | 2 | 168 | 58 | 21 | 482 | 35.22 | 6.3 | 7.2 | 23.8 | 708000000 | 1.96E+10 | 701000000 | 14748312 | 2E+06 |
| r4.34 | S057 | | T2 | 77197 | Ribo100 | RiboCom | 2 | 168 | 58 | 21 | 668 | 34.51 | 2.7 | 3.1 | 10.9 | 3190000000 | 9.14E+09 | 737000000 | 73741562 | 6E+06 |
| r4.35 | S057 | | T3 | 89526 | Ribo100 | RiboCom | 2 | 168 | 58 | 21 | 1660 | 25.26 | 4.4 | 4.4 | 13.8 | 1060000000 | 1.4E+10 | 1.18E+09 | 29496625 | 3E+06 |
| r4.36 | S057 | | T4 | 84250 | Ribo100 | RiboCom | 2 | 168 | 58 | 21 | 396 | 8.32 | 2.1 | 3.1 | 10.2 | 1170000000 | 1.03E+10 | 1.4E+09 | 2.21E+08 | 3E+06 |
| r4.37 | S058 | | T1 | 63983 | Ribo100 | RiboCom | 2 | 161 | 52 | 20 | 656 | 20.88 | 14.4 | 13.6 | 38.9 | 752000000 | 7.08E+09 | 1.51E+09 | 58993249 | 3E+06 |
| r4.38 | S058 | | T2 | 83299 | Ribo100 | RiboCom | 2 | 161 | 52 | 20 | 905 | 22.68 | 8 | 9.9 | 31.4 | 58900000 | 2.32E+10 | 2.65E+09 | 1.18E+08 | 7E+06 |
| r4.39 | S058 | | T3 | 82712 | Ribo100 | RiboCom | 2 | 161 | 52 | 20 | 3610 | 29.58 | 23.4 | 18.3 | 51.3 | 1420000000 | 3.05E+10 | 4.42E+09 | 2.06E+08 | 1E+06 |
| r4.4 | S061 | | T4 | 71228 | Ribo50 | RiboCom | 2 | 167 | 52 | 19 | 2340 | 25.86 | 12.7 | 19.1 | 55.3 | 575000000 | 1.09E+10 | 1.62E+09 | 73741562 | 5E+06 |
| r4.40 | S058 | | T4 | 90529 | Ribo100 | RiboCom | 2 | 161 | 52 | 20 | 994 | 32.11 | 16.2 | 13.8 | 37.4 | 1250000000 | 3.23E+10 | 1.25E+09 | 1.03E+08 | 2E+06 |
| r4.41 | S059 | | T1 | 80806 | Ribo100 | RiboCom | 1 | 193 | 88 | 24 | 959 | 20.82 | 6.7 | 27.3 | 50.7 | 1550000000 | 1.03E+10 | 2.47E+09 | 1.14E+09 | 369000 |
| r4.42 | S059 | | T2 | 73581 | Ribo100 | RiboCom | 1 | 193 | 88 | 24 | 574 | 25.3 | 10.2 | 23.7 | 66.5 | 1430000000 | 9.29E+09 | 2.36E+09 | 2.36E+08 | 369000 |
| r4.43 | S059 | | T3 | 71015 | Ribo100 | RiboCom | 1 | 193 | 88 | 24 | 20800 | 18.51 | 13.4 | 23.2 | 75.6 | 826000000 | 1.27E+10 | 2.62E+09 | 5.75E+08 | 3E+06 |
| r4.44 | S059 | | T4 | 87448 | Ribo100 | RiboCom | 1 | 193 | 88 | 24 | 1250 | 20.98 | 13.8 | 28 | 79.5 | 1170000000 | 3.61E+10 | 4.09E+09 | 5.16E+08 | 2E+06 |
| r4.45 | S060 | | T1 | 145402 | Ribo50 | RiboCom | 2 | 171 | 60 | 20 | 1200 | 34.19 | 5.5 | 4.8 | 15.1 | 737000000 | 1.47E+10 | 1.81E+09 | 5.46E+08 | 3E+06 |
| r4.46 | S060 | | T2 | 82839 | Ribo50 | RiboCom | 2 | 171 | 60 | 20 | 970 | 35.8 | 4.7 | 5 | 16.6 | 103000000 | 6.78E+09 | 3.32E+09 | 3.89E+09 | 5E+06 |
| r4.47 | S060 | | T3 | 77418 | Ribo50 | RiboCom | 2 | 171 | 60 | 20 | 2460 | 27.95 | 3.6 | 4.6 | 13.7 | 693000000 | 6.49E+09 | 2.14E+09 | 5.6E+08 | 2E+06 |
| r4.48 | S060 | | T4 | 83002 | Ribo50 | RiboCom | 2 | 171 | 60 | 20 | 2010 | 32.94 | 4.1 | 4.4 | 13.1 | 354000000 | 7.82E+09 | 1.81E+09 | 1.31E+09 | 4E+06 |
| r4.49 | S073 | | T1 | 61356 | Ribo100 | RiboCom | 2 | 170 | 54 | 19 | 1910 | 24.2 | 14.6 | 15 | 59.9 | 1135620047 | 2.463E+10 | 1.438E+09 | 1.62E+09 | 2E+06 |
| r4.5 | S063 | | T1 | 69323 | Placebo | Placebo | 2 | 178 | 63 | 20 | 2010 | 21.83 | 20.8 | 12.9 | 55.1 | 826000000 | 1.62E+10 | 442000000 | 3.39E+08 | 369000 |
| r4.50 | S073 | | T2 | 87252 | Ribo100 | RiboCom | 2 | 170 | 54 | 19 | 2480 | 25.42 | 6.6 | 7.4 | 47.6 | 1224109921 | 1.077E+10 | 1.327E+09 | 1.64E+09 | 737416 |
| r4.51 | S073 | | T3 | 80551 | Ribo100 | RiboCom | 2 | 170 | 54 | 19 | 12875 | 15.96 | 15.4 | 13.7 | 58.6 | 870150426 | 3.097E+09 | 294966246 | 5.9E+08 | 1E+06 |
| r4.52 | S073 | | T4 | 57946 | Ribo100 | RiboCom | 2 | 170 | 54 | 19 | 1220 | 17.74 | 9.6 | 11.9 | 53.4 | 1887783975 | 7.817E+09 | 921769519 | 9.14E+08 | 368708 |
| r4.53 | S085 | | T1 | 61565 | Ribo100 | RiboCom | 2 | 172 | 62 | 21 | 3460 | 21.95 | 5.9 | 6.7 | 26.8 | 3451105079 | 3.112E+10 | 9.771E+09 | 1.21E+09 | 368708 |
| r4.54 | S085 | | T2 | 54901 | Ribo100 | RiboCom | 2 | 172 | 62 | 21 | 2310 | 33.85 | 12.8 | 9.8 | 57 | 2492464779 | 2.419E+10 | 5.899E+09 | 1.28E+09 | 737416 |
| r4.55 | S085 | | T3 | 61541 | Ribo100 | RiboCom | 2 | 172 | 62 | 21 | 3760 | 29.52 | 10 | 7.8 | 37.5 | 4159024070 | 2.846E+10 | 2.655E+09 | 2.11E+09 | 1E+06 |
| r4.56 | S085 | | T4 | 70651 | Ribo100 | RiboCom | 2 | 172 | 62 | 21 | 2520 | 27.99 | 6.2 | 5 | 23.5 | 6857965222 | 3.997E+10 | 3.908E+09 | 2.76E+09 | 369000 |
| r4.57 | S098 | | T1 | 62449 | Ribo50 | RiboCom | 2 | 171 | 59 | 20 | 2530 | 29.46 | NA | NA | NA | 7654374086 | 3.023E+10 | 4.609E+09 | 4.14E+09 | 2E+06 |
| r4.58 | S098 | | T2 | 59079 | Ribo50 | RiboCom | 2 | 171 | 59 | 20 | 2660 | 22.42 | NA | NA | NA | 1814042413 | 5.147E+10 | 2.175E+09 | 1.11E+09 | 369000 |
| r4.59 | S098 | | T3 | 59528 | Ribo50 | RiboCom | 2 | 171 | 59 | 20 | 5260 | 25.85 | NA | NA | NA | 1356844732 | 1.311E+11 | 3.613E+09 | 8.7E+08 | 737416 |
| r4.6 | S063 | | T2 | 65739 | Placebo | Placebo | 2 | 178 | 63 | 20 | 2350 | 15.11 | 18.2 | 11.6 | 50.2 | 471000000 | 8.41E+09 | 922000000 | 2.06E+08 | 369000 |
| r4.60 | S098 | | T4 | 62501 | Ribo50 | RiboCom | 2 | 171 | 59 | 20 | 2560 | 35.32 | NA | NA | NA | 884898738 | 6.18E+10 | 2.802E+09 | 8.11E+08 | 369000 |
| r4.61 | S100 | | T1 | 56163 | Placebo | Placebo | 2 | 149 | 62 | 28 | 3260 | 20 | 16.1 | 11.9 | 36 | 14700000 | 737415615 | 110612342 | 14700000 | 369000 |
| r4.62 | S100 | | T2 | 65041 | Placebo | Placebo | 2 | 149 | 62 | 28 | 3470 | 24.07 | 5.1 | 8.9 | 41.4 | 14700000 | 1.032E+09 | 73741562 | 14700000 | 368708 |
| r4.63 | S100 | | T3 | 63146 | Placebo | Placebo | 2 | 149 | 62 | 28 | 3020 | 28.7 | 8 | 7.3 | 39.7 | 14700000 | 2.61E+10 | 184353904 | 14700000 | 369000 |
| r4.64 | S100 | | T4 | 70053 | Placebo | Placebo | 2 | 149 | 62 | 28 | 2840 | 23.42 | 20.9 | 11.5 | 76.8 | 14700000 | 1.18E+09 | 36870781 | 14700000 | 369000 |
| r4.65 | S101 | | T1 | 53159 | Placebo | Placebo | 1 | 178 | 84 | 27 | 1990 | 12.79 | 11 | 25.1 | 47.9 | 14700000 | 884898738 | 110612342 | 14700000 | 369000 |
| r4.66 | S101 | | T2 | 60738 | Placebo | Placebo | 1 | 178 | 84 | 27 | 2760 | 28.81 | 15.6 | 19.6 | 51 | 14700000 | 1.814E+10 | 147483123 | 14700000 | 369000 |
| r4.67 | S101 | | T3 | 58774 | Placebo | Placebo | 1 | 178 | 84 | 27 | 3380 | 26.32 | 11.3 | 11.2 | 40.7 | 14700000 | 1.327E+09 | 36870781 | 14700000 | 369000 |
| r4.68 | S101 | | T4 | 59335 | Placebo | Placebo | 1 | 178 | 84 | 27 | 962 | 27.12 | 8.7 | 12 | 33.2 | 14700000 | 1.327E+09 | 147483123 | 14700000 | 369000 |
| r4.69 | S114 | | T1 | 55584 | Placebo | Placebo | 2 | 167 | 65 | 23 | 302 | 12.19 | 7.8 | 3.6 | 27.6 | 14700000 | 2.212E+09 | 700544835 | 29496625 | 369000 |
| r4.7 | S063 | | T3 | 61243 | Placebo | Placebo | 2 | 178 | 63 | 20 | 2330 | 23.44 | 12.6 | 10.8 | 43.5 | 443000000 | 8.11E+09 | 811000000 | 2.95E+08 | 369000 |
| r4.70 | S114 | | T2 | 73562 | Placebo | Placebo | 2 | 167 | 65 | 23 | 3410 | 31.16 | 15.2 | 19.2 | 55.6 | 58993249.2 | 3.245E+09 | 405578588 | 14700000 | 368708 |
| r4.71 | S114 | | T3 | 61304 | Placebo | Placebo | 2 | 167 | 65 | 23 | 2620 | 29.64 | 14.5 | 18.4 | 67.7 | 29496624.7 | 3.982E+09 | 110612343 | 1.77E+08 | 369000 |
| r4.72 | S114 | | T4 | 68248 | Placebo | Placebo | 2 | 167 | 65 | 23 | 2800 | 27.56 | 8.1 | 12.7 | 39.7 | 103238186 | 2.065E+09 | 36870781 | 1.62E+08 | 369000 |
| r4.8 | S063 | | T4 | 66656 | Placebo | Placebo | 2 | 178 | 63 | 20 | 1700 | 18.71 | 26.4 | 13.4 | 68.1 | 399000000 | 4.87E+09 | 1.11E+09 | 3.69E+08 | 737000 |
| r4.9 | S064 | | T1 | 60506 | Ribo50 | RiboCom | 1 | 170 | 70 | 24 | 3280 | 27.05 | 28.9 | 31.7 | 86.1 | 398000000 | 3.01E+10 | 2.65E+09 | 4.72E+08 | 369000 |
| r5.49 | S045 | | T1 | 170670 | Placebo | Placebo | 2 | 172 | 64 | 22 | 2490 | 21.87 | 14.3 | 12.8 | 50.8 | 1017633549 | 3.053E+10 | 4.683E+09 | 7.67E+08 | 3E+06 |
| r5.50 | S045 | | T2 | 258728 | Placebo | Placebo | 2 | 172 | 64 | 22 | 2040 | 28.67 | 8.1 | 8.2 | 32.5 | 14748312 | 1.077E+10 | 1.733E+09 | 58993249 | 368708 |
| r5.51 | S045 | | T3 | 139238 | Placebo | Placebo | 2 | 172 | 64 | 22 | 1670 | 29.87 | 9.6 | 9.4 | 31.1 | 1268354858 | 9.734E+09 | 2.249E+09 | 5.01E+08 | 368708 |
| r5.52 | S045 | | T4 | 62685 | Placebo | Placebo | 2 | 172 | 64 | 22 | 1700 | 21.51 | 16.7 | 16.9 | 61.6 | 3141390521 | 2.507E+10 | 2.065E+09 | 6.49E+08 | 2E+06 |
| r5.54 | S046 | | T2 | 140931 | Ribo100 | RiboCom | 2 | 167 | 56 | 20 | 1620 | 12.53 | 28.9 | 15.5 | 99 | 1150368360 | 3.407E+10 | 3.245E+09 | 8.85E+08 | 369000 |
| r5.55 | S046 | | T3 | 125717 | Ribo100 | RiboCom | 2 | 167 | 56 | 20 | 137000 | 19.55 | 15.1 | 20.6 | 53.9 | 604680804 | 6.946E+10 | 1.043E+10 | 2.93E+09 | 737416 |
| r5.56 | S046 | | T4 | 36394 | Ribo100 | RiboCom | 2 | 167 | 56 | 20 | 559 | 13.84 | 37.2 | 15.3 | 97.8 | 634177429 | 2.212E+10 | 1.412E+10 | 2.06E+08 | 368708 |
| r5.57 | S051 | | T1 | 178202 | Placebo | Placebo | 2 | 169 | 61 | 21 | 2350 | 27.68 | 18.7 | 22.9 | 67.4 | 265469621 | 1.74E+10 | 2.36E+09 | 9E+08 | 368708 |
| r5.58 | S051 | | T2 | 95410 | Placebo | Placebo | 2 | 169 | 61 | 21 | 2830 | 20.79 | 14.3 | 34.1 | 62.8 | 2831675963 | 2.153E+10 | 3.355E+09 | 9.14E+08 | 2E+06 |
| r5.59 | S051 | | T3 | 126625 | Placebo | Placebo | 2 | 169 | 61 | 21 | 1850 | 27.46 | 13.8 | 26.1 | 50.9 | 1740300852 | 1.431E+10 | 3.871E+09 | 1.81E+09 | 2E+06 |
| r5.60 | S051 | | T4 | 159843 | Placebo | Placebo | 2 | 169 | 61 | 21 | 3050 | 24.81 | 18.6 | 24.9 | 65.5 | 1519076167 | 1.354E+11 | 1.316E+10 | 9.14E+08 | 2E+06 |
| r5.61 | S074 | | T1 | 45970 | Ribo50 | RiboCom | 1 | 193 | 90 | 24 | NA | 27.08 | 8 | 8.7 | 25.8 | 2109008660 | 5.014E+09 | 1.475E+09 | 1.25E+09 | 1E+07 |
| r5.62 | S074 | | T2 | 137003 | Ribo50 | RiboCom | 1 | 193 | 90 | 24 | 1120 | 21.57 | 3.8 | 6.3 | 30.3 | 6415515853 | 2.655E+10 | 1.549E+09 | 5.97E+09 | 6E+06 |
| r5.64 | S074 | | T4 | 176169 | Ribo50 | RiboCom | 1 | 193 | 90 | 24 | 1400 | 23.32 | 7.7 | 5.1 | 50.6 | 1.1209E+10 | 3.333E+10 | 4.13E+09 | 6.92E+09 | 3E+06 |
| r5.65 | S075 | | T1 | 99627 | Placebo | Placebo | 2 | 176 | 64 | 21 | 2220 | 23.42 | 24.3 | 8.1 | 65.4 | 1489579543 | 1.77E+10 | 1.733E+09 | 2.17E+09 | 1E+06 |
| r5.66 | S075 | | T2 | 30206 | Placebo | Placebo | 2 | 176 | 64 | 21 | 2590 | 25.5 | 26.5 | 13.6 | 76.4 | 4999677871 | 3.628E+10 | 4.83E+09 | 4.84E+09 | 1E+06 |
| r5.67 | S075 | | T3 | 88395 | Placebo | Placebo | 2 | 176 | 64 | 21 | 2620 | 24.93 | 10.6 | 10.6 | 46.8 | 1.7993E+10 | 3.658E+10 | 3.318E+09 | 8.85E+09 | 2E+06 |
| r5.68 | S075 | | T4 | 71395 | Placebo | Placebo | 2 | 176 | 64 | 21 | 2780 | 24.98 | 13.2 | 9.8 | 57.2 | 2743186089 | 1.209E+10 | 2.655E+09 | 8.7E+08 | 3E+06 |
| r5.69 | S077 | | T1 | 181625 | Ribo100 | RiboCom | 2 | 171 | 59 | 20 | 3030 | 28.02 | 16 | 12 | 65.3 | 1504327855 | 9.173E+10 | 2.024E+10 | 9.04E+09 | 737416 |
| r5.70 | S077 | | T2 | 44426 | Ribo100 | RiboCom | 2 | 171 | 59 | 20 | 2770 | 30.46 | 13.4 | 12.8 | 58.3 | 4424493691 | 9.291E+10 | 1.102E+10 | 6.89E+09 | 4E+06 |
| r5.71 | S077 | | T3 | 95543 | Ribo100 | RiboCom | 2 | 171 | 59 | 20 | 285000 | 28.5 | 17.5 | 11 | 63.4 | 1.4203E+10 | 4.955E+10 | 7.116E+09 | 6.02E+09 | 1E+06 |
| r5.72 | S077 | | T4 | 126763 | Ribo100 | RiboCom | 2 | 171 | 59 | 20 | 2400 | 21.95 | 11.2 | 11.3 | 50.4 | 516190931 | 6.887E+10 | 1.121E+10 | 1.28E+09 | 1E+06 |
| r5.73 | S088 | | T1 | 139065 | Placebo | Placebo | 2 | 163 | 71 | 27 | 2020 | 32.92 | 21.1 | 14.9 | 44.2 | 545687555 | 3.097E+10 | 2.802E+09 | 7.52E+08 | 737416 |
| r5.74 | S088 | | T2 | 216174 | Placebo | Placebo | 2 | 163 | 71 | 27 | 2280 | 29.27 | 21.7 | 23.6 | 71.5 | 3731323013 | 6.43E+10 | 1.729E+10 | 5.34E+09 | 737416 |
| r5.75 | S088 | | T3 | 91784 | Placebo | Placebo | 2 | 163 | 71 | 27 | 2410 | 28.11 | 8.3 | 6.1 | 24.8 | 117986498 | 1.333E+11 | 1.707E+10 | 1.83E+09 | 369000 |
| r5.76 | S088 | | T4 | 61948 | Placebo | Placebo | 2 | 163 | 71 | 27 | 3320 | NA | NA | NA | NA | 2684192839 | 7.448E+10 | 1.6E+10 | 2.14E+09 | 369000 |
| r5.77 | S104 | | T1 | 83659 | Placebo | Placebo | 2 | 137 | 54 | 29 | 1930 | 19.78 | 15.3 | 10.5 | 56.4 | 4114779133 | 7.595E+10 | 4.093E+09 | 4.44E+09 | 737416 |
| r5.78 | S104 | | T2 | 54925 | Placebo | Placebo | 2 | 137 | 54 | 29 | 2420 | 39.04 | 21.7 | 17.6 | 70.2 | 4350752130 | 6.46E+10 | 2.47E+09 | 2.04E+09 | 1E+06 |
| r5.79 | S104 | | T3 | 459550 | Placebo | Placebo | 2 | 137 | 54 | 29 | 1540 | 24.76 | 9 | 9.3 | 55.6 | 398204432 | 6.401E+10 | 3.134E+09 | 2.21E+09 | 368708 |
| r5.80 | S104 | | T4 | 238767 | Placebo | Placebo | 2 | 137 | 54 | 29 | 2300 | 29.61 | 16.5 | 14.1 | 64.9 | 2418723218 | 1.024E+11 | 7.227E+09 | 5.28E+09 | 368708 |
| r5.81 | S105 | | T1 | 69583 | Placebo | Placebo | 1 | 184 | 73 | 22 | 2910 | 27.65 | 15.1 | 15.2 | 65.5 | 1563321104 | 1.67E+11 | 2.223E+10 | 8.11E+08 | 2E+06 |
| r5.82 | S105 | | T2 | 107866 | Placebo | Placebo | 1 | 184 | 73 | 22 | 2300 | 28.42 | 12.8 | 21.4 | 50.3 | 471945994 | 3.643E+10 | 4.572E+09 | 1.93E+09 | 1E+06 |
| r5.83 | S105 | | T3 | 100144 | Placebo | Placebo | 1 | 184 | 73 | 22 | 1940 | 19.58 | 17.7 | 25.1 | 58.5 | 280217934 | 7.227E+10 | 1.025E+10 | 1.59E+09 | 369000 |
| r5.84 | S105 | | T4 | 46681 | Placebo | Placebo | 1 | 184 | 73 | 22 | 1430 | 21.71 | 15.3 | 26.4 | 71.9 | 1430586294 | 1.667E+10 | 1.807E+09 | 1.36E+09 | 6E+06 |
| r5.85 | S108 | | T1 | 130668 | Placebo | Placebo | 1 | 182 | 71 | 21 | 2260 | 34.81 | 9.2 | 8.7 | 50 | 3067648959 | 1.518E+11 | 1.055E+10 | 1.35E+10 | 1E+06 |
| r5.86 | S108 | | T2 | 888965 | Placebo | Placebo | 1 | 182 | 71 | 21 | 3330 | 36.2 | 10.1 | 10 | 36.2 | 2168001909 | 8.112E+10 | 5.088E+09 | 2.63E+09 | 737416 |
| r5.87 | S108 | | T3 | 80229 | Placebo | Placebo | 1 | 182 | 71 | 21 | 2450 | 18.58 | 15.1 | 16.8 | 60.8 | 1283103171 | 6.062E+10 | 1.268E+10 | 2.68E+09 | 3E+06 |
| r5.88 | S108 | | T4 | 53332 | Placebo | Placebo | 1 | 182 | 71 | 21 | 3020 | 28.86 | 8.2 | 11.9 | 41.4 | 73741562 | 5.604E+10 | 1.132E+10 | 8.55E+08 | 1E+06 |
| r5.89 | S109 | | T1 | 67160 | Ribo100 | RiboCom | 1 | 173 | 66 | 22 | 4010 | 25.7 | 15.6 | 22.5 | 65 | 206476372 | 5.752E+10 | 6.231E+09 | 2.36E+08 | 737416 |
| r5.91 | S109 | | T3 | 264211 | Ribo100 | RiboCom | 1 | 173 | 66 | 22 | 90600 | 16.68 | 24.5 | 50.9 | 95 | 412952745 | 3.894E+10 | 2.839E+09 | 5.01E+08 | 737416 |
| r5.92 | S109 | | T4 | 74500 | Ribo100 | RiboCom | 1 | 173 | 66 | 22 | 2640 | 24.72 | 15.8 | 18 | 54.6 | 2.1149E+10 | 8.864E+10 | 6.969E+09 | 9.29E+08 | 737416 |
| r6.1 | S110 | | T1 | 59384 | Ribo100 | RiboCom | 1 | 187 | 76 | 22 | 2940 | 28.1 | 18 | 18.4 | 60.1 | 4277010568 | 1.582E+11 | 2.946E+10 | 5.35E+09 | 737416 |
| r6.10 | S113 | | T2 | 1859224 | Ribo50 | RiboCom | 2 | 176 | 64 | 21 | 1980 | 33.44 | 5.1 | 4 | 25 | 324462871 | 4.631E+10 | 7.19E+09 | 2.08E+09 | 737416 |
| r6.11 | S113 | | T3 | 70095 | Ribo50 | RiboCom | 2 | 176 | 64 | 21 | 4500 | 27.81 | 22.7 | 11 | 61.1 | 722667303 | 4.941E+10 | 3.392E+09 | 1.21E+09 | 737416 |
| r6.12 | S113 | | T4 | 105493 | Ribo50 | RiboCom | 2 | 176 | 64 | 21 | 1320 | 25.13 | 3.4 | 5.7 | 27.8 | 486694306 | 2.021E+10 | 1.696E+09 | 9.59E+08 | 2E+06 |
| r6.17 | S116 | | T1 | 226735 | Placebo | Placebo | 2 | 167 | 63 | 22 | 2260 | 21.6 | 5.6 | 9.2 | 33.2 | 2109008660 | 3.141E+10 | 2.102E+09 | 1.42E+09 | 1E+06 |
| r6.18 | S116 | | T2 | 89883 | Placebo | Placebo | 2 | 167 | 63 | 22 | 1790 | 18.36 | 23.1 | 21.4 | 83.4 | 324462871 | 3.687E+10 | 1.659E+09 | 1.45E+09 | 369000 |
| r6.19 | S116 | | T3 | 61557 | Placebo | Placebo | 2 | 167 | 63 | 22 | 1480 | 35.88 | 22 | 15.8 | 40.5 | 309714558 | 3.318E+10 | 1.29E+09 | 5.31E+08 | 3E+06 |
| r6.2 | S110 | | T2 | 64813 | Ribo100 | RiboCom | 1 | 187 | 76 | 22 | 2650 | 27.6 | 16.3 | 14 | 61.1 | 5.538E+10 | 2.515E+11 | 4.192E+10 | 4.22E+09 | 368708 |
| r6.20 | S116 | | T4 | 53017 | Placebo | Placebo | 2 | 167 | 63 | 22 | 1720 | 15.94 | 32.6 | 28.1 | 98.4 | 929143675 | 1.932E+10 | 1.807E+09 | 8.7E+08 | 1E+06 |
| r6.21 | S122 | | T1 | 95257 | Ribo50 | RiboCom | 2 | 167 | 55 | 20 | 2130 | 28.49 | 8.3 | 6.9 | 37 | 589932492 | 5.575E+10 | 6.084E+09 | 4.96E+09 | 3E+06 |
| r6.22 | S122 | | T2 | 54595 | Ribo50 | RiboCom | 2 | 167 | 55 | 20 | 2910 | 37.08 | 6.9 | 8 | 24.1 | 634177429 | 3.849E+10 | 3.576E+09 | 4.72E+08 | 3E+06 |
| r6.23 | S122 | | T3 | 53989 | Ribo50 | RiboCom | 2 | 167 | 55 | 20 | 3740 | 35.61 | 9.4 | 8.1 | 34.2 | 4719459938 | 8.76E+10 | 3.407E+10 | 3.23E+10 | 1E+06 |
| r6.24 | S122 | | T4 | 60360 | Ribo50 | RiboCom | 2 | 167 | 55 | 20 | 1750 | 37.01 | 13.8 | 13.9 | 39.5 | 235972997 | 6.725E+10 | 1.316E+10 | 2.4E+09 | 368708 |
| r6.25 | S124 | | T1 | 39749 | Ribo50 | RiboCom | 1 | 192 | 89 | 24 | 2760 | 25.5 | 32.9 | 39.1 | 88.6 | 206476372 | 2.787E+10 | 1.051E+10 | 2.4E+09 | 1E+06 |
| r6.26 | S124 | | T2 | 81123 | Ribo50 | RiboCom | 1 | 192 | 89 | 24 | 2500 | 32.82 | 15.8 | 21.1 | 48.3 | 294966246 | 3.923E+10 | 1.659E+09 | 7.23E+08 | 9E+06 |
| r6.27 | S124 | | T3 | 151671 | Ribo50 | RiboCom | 1 | 192 | 89 | 24 | 3140 | 25.52 | 17.3 | 27 | 62.6 | 324462871 | 3.112E+10 | 3.281E+09 | 5.9E+08 | 3E+06 |
| r6.28 | S124 | | T4 | 54792 | Ribo50 | RiboCom | 1 | 192 | 89 | 24 | 1350 | 16.19 | 13.4 | 28.4 | 79.9 | 309714558 | 2.094E+10 | 2.065E+09 | 9E+08 | 2E+06 |
| r6.29 | S126 | | T1 | 68594 | Ribo100 | RiboCom | 2 | 174 | 72 | 24 | 2310 | 24.96 | 4.5 | 9.3 | 44.9 | 1002885237 | 1.203E+11 | 7.448E+09 | 3.85E+09 | 1E+07 |
| r6.3 | S110 | | T3 | 77404 | Ribo100 | RiboCom | 1 | 187 | 76 | 22 | 1220 | 44.08 | 6.6 | 5.5 | 19.3 | 1194613297 | 5.737E+10 | 1.018E+10 | 1.18E+09 | 3E+06 |
| r6.30 | S126 | | T2 | 81417 | Ribo100 | RiboCom | 2 | 174 | 72 | 24 | 3620 | 27.73 | 7.6 | 12.2 | 57.2 | 530939243 | 1.015E+11 | 5.383E+09 | 1.71E+09 | 369000 |
| r6.31 | S126 | | T3 | 69554 | Ribo100 | RiboCom | 2 | 174 | 72 | 24 | 13200 | 23.4 | 11.1 | 7.9 | 46.6 | 324462871 | 1.029E+11 | 5.604E+09 | 1.3E+09 | 3E+06 |
| r6.32 | S126 | | T4 | 55440 | Ribo100 | RiboCom | 2 | 174 | 72 | 24 | 2370 | 28.5 | 3.1 | 5.4 | 25.9 | 1179864984 | 1.299E+11 | 1.807E+09 | 2.33E+09 | 1E+06 |
| r6.33 | S128 | | T1 | 111328 | Placebo | Placebo | 2 | 171 | 68 | 23 | 2010 | 17.72 | 12.4 | 34.3 | 64.2 | 44244937 | 2.551E+10 | 2.139E+09 | 4.72E+08 | 4E+06 |
| r6.34 | S128 | | T2 | 78665 | Placebo | Placebo | 2 | 171 | 68 | 23 | 2060 | 24.77 | 15.3 | 28.8 | 55 | 1932028912 | 3.687E+10 | 3.134E+09 | 1.86E+09 | 737416 |
| r6.35 | S128 | | T3 | 253186 | Placebo | Placebo | 2 | 171 | 68 | 23 | 2020 | 27.59 | 9.6 | 14.5 | 37.5 | 870150426 | 3.377E+10 | 3.355E+09 | 1.43E+09 | 2E+06 |
| r6.36 | S128 | | T4 | 68799 | Placebo | Placebo | 2 | 171 | 68 | 23 | 2220 | 25.92 | 17.6 | 24.2 | 50.1 | 383456120 | 7.684E+10 | 3.208E+09 | 2.18E+09 | 1E+06 |
| r6.37 | S130 | | T1 | 72695 | Ribo50 | RiboCom | 1 | 185 | 78 | 23 | 3090 | 26.32 | 10.3 | 10.6 | 32.8 | 44244937 | 7.876E+10 | 4.056E+09 | 1.92E+08 | 737416 |
| r6.38 | S130 | | T2 | 66800 | Ribo50 | RiboCom | 1 | 185 | 78 | 23 | 2540 | 23.91 | 26 | 23.8 | 61.6 | 2920165836 | 3.082E+10 | 4.978E+09 | 1.03E+09 | 2E+06 |
| r6.39 | S130 | | T3 | 117841 | Ribo50 | RiboCom | 1 | 185 | 78 | 23 | 75000 | 32.63 | 17.5 | 18.4 | 56.2 | 4203269007 | 2.758E+10 | 2.987E+09 | 2.3E+09 | 369000 |
| r6.4 | S110 | | T4 | 113815 | Ribo100 | RiboCom | 1 | 187 | 76 | 22 | 3080 | 32.07 | 19.1 | 20.3 | 68.6 | 5206154244 | 1.289E+11 | 1.342E+10 | 3.14E+09 | 737416 |
| r6.40 | S130 | | T4 | 68918 | Ribo50 | RiboCom | 1 | 185 | 78 | 23 | 1670 | 23.19 | 26 | 13.2 | 45.8 | 1755049164 | 3.097E+10 | 1.88E+09 | 1.8E+09 | 737416 |
| r6.41 | S139 | | T1 | 40192 | Ribo50 | RiboCom | 1 | 162 | 53 | 20 | 2980 | 23.72 | 12.5 | 15.4 | 36.4 | 1194613297 | 7.61E+10 | 1.165E+10 | 1.12E+10 | 3E+06 |
| r6.42 | S139 | | T2 | 1002179 | Ribo50 | RiboCom | 1 | 162 | 53 | 20 | 1630 | 28.59 | 18.6 | 26.2 | 59.3 | 324462871 | 1.726E+10 | 2.36E+09 | 2.09E+09 | 1E+06 |
| r6.43 | S139 | | T3 | 72643 | Ribo50 | RiboCom | 1 | 162 | 53 | 20 | 102000 | 21.37 | 7.6 | 19.7 | 43.8 | 427701057 | 8.702E+09 | 6.305E+09 | 29496625 | 6E+06 |
| r6.44 | S139 | | T4 | 86058 | Ribo50 | RiboCom | 1 | 162 | 53 | 20 | 3870 | 27.07 | 7.1 | 16.9 | 36.3 | 206476372 | 1.77E+10 | 4.351E+09 | 2.93E+09 | 4E+06 |
| r6.45 | S140 | | T1 | 108083 | Placebo | Placebo | 2 | 160 | 57 | 22 | 1980 | 21.56 | 12.9 | 12 | 62.3 | 176979748 | 3.422E+10 | 1.589E+10 | 2.34E+09 | 1E+06 |
| r6.46 | S140 | | T2 | 69368 | Placebo | Placebo | 2 | 160 | 57 | 22 | 2330 | 23.08 | 5.3 | 8 | 34.9 | 707918991 | 1.013E+11 | 5.973E+09 | 5.32E+09 | 737416 |
| r6.47 | S140 | | T3 | 39962 | Placebo | Placebo | 2 | 160 | 57 | 22 | 3110 | 26.25 | 6.2 | 7.4 | 29.4 | 2610451278 | 7.935E+10 | 6.858E+09 | 6.93E+08 | 368708 |
| r6.48 | S140 | | T4 | 67374 | Placebo | Placebo | 2 | 160 | 57 | 22 | 3690 | 30.2 | 13.7 | 9.7 | 41 | 117986498 | 6.357E+10 | 6.932E+09 | 1.42E+09 | 737416 |
| r6.49 | S141 | | T1 | 81050 | Ribo100 | RiboCom | 1 | 180 | 74 | 23 | 1320 | 34.06 | 8.8 | 11.1 | 30.8 | 2979159086 | 1.947E+10 | 2.397E+09 | 7.37E+08 | 1E+06 |
| r6.5 | S112 | | T1 | 131744 | Ribo50 | RiboCom | 2 | 170 | 54 | 19 | 2590 | 24.69 | 29.6 | 21.5 | 72.9 | 442449369 | 6.489E+10 | 6.489E+09 | 1.18E+09 | 737416 |
| r6.50 | S141 | | T2 | 118348 | Ribo100 | RiboCom | 1 | 180 | 74 | 23 | 1590 | 26.76 | 20.7 | 13.8 | 61.2 | 1755049164 | 4.292E+10 | 3.54E+09 | 8.11E+08 | 1E+06 |
| r6.51 | S141 | | T3 | 70889 | Ribo100 | RiboCom | 1 | 180 | 74 | 23 | 66900 | 20.24 | 24.9 | 13.6 | 73.2 | 73741562 | 4.955E+10 | 3.798E+09 | 3.83E+08 | 737416 |
| r6.52 | S141 | | T4 | 161259 | Ribo100 | RiboCom | 1 | 180 | 74 | 23 | 2900 | 28.56 | 33.3 | 25.5 | 68.4 | 1504327855 | 5.693E+10 | 1.217E+09 | 1.33E+08 | 1E+06 |
| r6.53 | S142 | | T1 | 39668 | Ribo100 | RiboCom | 2 | 174 | 64 | 21 | 2340 | 34.42 | 23.4 | 14.1 | 67.7 | 545687555 | 8.185E+10 | 1.497E+10 | 2.39E+09 | 1E+07 |
| r6.54 | S142 | | T2 | 76866 | Ribo100 | RiboCom | 2 | 174 | 64 | 21 | 2660 | 32.68 | 16 | 14 | 56.2 | 132734811 | 1.844E+11 | 6.858E+09 | 1.49E+09 | 6E+06 |
| r6.55 | S142 | | T3 | 74665 | Ribo100 | RiboCom | 2 | 174 | 64 | 21 | 4930 | 27.98 | 19.3 | 13 | 51 | 353959495 | 8.303E+10 | 1.394E+10 | 8.64E+09 | 737416 |
| r6.56 | S142 | | T4 | 167044 | Ribo100 | RiboCom | 2 | 174 | 64 | 21 | 3310 | 31.04 | 32.5 | 22.4 | 75.4 | 88489874 | 4.41E+10 | 6.674E+09 | 5.9E+09 | 369000 |
| r6.57 | S146 | | T1 | 71445 | Ribo50 | RiboCom | 2 | 172 | 62 | 21 | 3210 | 28.17 | 8 | 8.2 | 42.8 | 280217934 | 1.175E+11 | 3.868E+10 | 6.81E+09 | 737416 |
| r6.58 | S146 | | T2 | 82448 | Ribo50 | RiboCom | 2 | 172 | 62 | 21 | 3500 | 28.22 | 1.9 | 3.8 | 11.9 | 1076626798 | 1.313E+10 | 1.844E+09 | 6.64E+08 | 6E+06 |
| r6.59 | S146 | | T3 | 92480 | Ribo50 | RiboCom | 2 | 172 | 62 | 21 | 19100 | 27.39 | 17.1 | 12.6 | 51.5 | 678422366 | 2.227E+10 | 1.549E+09 | 4.87E+08 | 369000 |
| r6.6 | S112 | | T2 | 103106 | Ribo50 | RiboCom | 2 | 170 | 54 | 19 | 2980 | 21.93 | 41.2 | 22.9 | 79.6 | 2639947903 | 1.681E+11 | 9.881E+09 | 1.67E+09 | 1E+06 |
| r6.60 | S146 | | T4 | 1513352 | Ribo50 | RiboCom | 2 | 172 | 62 | 21 | 1910 | 29.52 | 14 | 10.6 | 57.4 | 280217934 | 2.714E+10 | 2.139E+09 | 3.24E+08 | 1E+06 |
| r6.61 | S147 | | T1 | 99000 | Ribo100 | RiboCom | 2 | 183 | 72 | 22 | 1790 | 26.47 | 13.2 | 10.7 | 27.8 | 1253606546 | 2.596E+10 | 2.47E+09 | 7.96E+08 | 2E+06 |
| r6.62 | S147 | | T2 | 95126 | Ribo100 | RiboCom | 2 | 183 | 72 | 22 | 3390 | 19.67 | 9.4 | 10.1 | 42.9 | 634177429 | 1.103E+11 | 4.498E+09 | 7.85E+09 | 3E+06 |
| r6.63 | S147 | | T3 | 109137 | Ribo100 | RiboCom | 2 | 183 | 72 | 22 | 5880 | 27.36 | 14.7 | 10.8 | 50.1 | 235972997 | 1.052E+11 | 1.18E+10 | 2.42E+09 | 1E+06 |
| r6.64 | S147 | | T4 | 70921 | Ribo100 | RiboCom | 2 | 183 | 72 | 22 | 4500 | 27.29 | 10.1 | 10.1 | 43.3 | 206476372 | 8.185E+10 | 9.734E+09 | 6.39E+09 | 737416 |
| r6.65 | S148 | | T1 | 101535 | Placebo | Placebo | 2 | 170 | 62 | 22 | 2750 | 22.62 | 18.4 | 11.9 | 59.7 | 1253606546 | 5.014E+10 | 4.461E+09 | 2.52E+09 | 737416 |
| r6.66 | S148 | | T2 | 114116 | Placebo | Placebo | 2 | 170 | 62 | 22 | 1810 | 16.94 | 27.7 | 18.2 | 71.2 | 1356844732 | 2.448E+10 | 1.844E+09 | 6.34E+08 | 3E+06 |
| r6.67 | S148 | | T3 | 122082 | Placebo | Placebo | 2 | 170 | 62 | 22 | 3050 | 24.86 | 16.3 | 14.7 | 45.5 | 589932492 | 5.767E+10 | 1.493E+10 | 2.39E+09 | 1E+06 |
| r6.68 | S148 | | T4 | 70322 | Placebo | Placebo | 2 | 170 | 62 | 22 | 3010 | 28.43 | 14.5 | 14.5 | 37.4 | 6872713534 | 6.666E+10 | 1.346E+10 | 3.05E+10 | 368708 |
| r6.69 | S149 | | T1 | 68373 | Placebo | Placebo | 2 | 163 | 66 | 25 | 3180 | 24.98 | 14.7 | 22.2 | 63.9 | 914395363 | 4.277E+10 | 9.107E+09 | 1.65E+09 | 369000 |
| r6.7 | S112 | | T3 | 82078 | Ribo50 | RiboCom | 2 | 170 | 54 | 19 | 5860 | 32.71 | 34.2 | 26.7 | 91 | 117986498 | 5.84E+10 | 1.479E+10 | 8.85E+08 | 737416 |
| r6.70 | S149 | | T2 | 50154 | Placebo | Placebo | 2 | 163 | 66 | 25 | 2280 | 24.02 | 10.6 | 17.2 | 50.8 | 2993907398 | 2.315E+10 | 3.392E+09 | 2.95E+09 | 369000 |
| r6.71 | S149 | | T3 | 96557 | Placebo | Placebo | 2 | 163 | 66 | 25 | 2950 | 26.67 | 14.5 | 16.7 | 52.1 | 176979748 | 2.861E+10 | 7.485E+09 | 1.78E+09 | 1E+06 |
| r6.72 | S149 | | T4 | 171925 | Placebo | Placebo | 2 | 163 | 66 | 25 | 3550 | 22.23 | 10.3 | 13.4 | 42.9 | 14748312 | 2.876E+10 | 5.604E+09 | 1.22E+09 | 737416 |
| r6.73 | S150 | | T1 | 1926084 | Ribo100 | RiboCom | 2 | 167 | 65 | 23 | 2280 | 28.68 | 2.1 | 4.9 | 17.6 | 501442618 | 3.289E+10 | 2.655E+09 | 3.83E+08 | 1E+06 |
| r6.74 | S150 | | T2 | 88559 | Ribo100 | RiboCom | 2 | 167 | 65 | 23 | 2510 | 32 | 2.6 | 5.3 | 24.3 | 412952745 | 3.643E+10 | 2.065E+09 | 3.54E+08 | 3E+06 |
| r6.75 | S150 | | T3 | 69190 | Ribo100 | RiboCom | 2 | 167 | 65 | 23 | 3360 | 29.09 | 24 | 20.6 | 68 | 796408864 | 7.227E+10 | 5.604E+09 | 3.2E+09 | 8E+06 |
| r6.76 | S150 | | T4 | 66481 | Ribo100 | RiboCom | 2 | 167 | 65 | 23 | 2270 | 27.1 | 6.2 | 8.8 | 29 | 353959495 | 4.218E+10 | 4.904E+09 | 2.68E+09 | 2E+07 |
| r6.77 | S152 | | T1 | 50827 | Ribo100 | RiboCom | 2 | 167 | 52 | 19 | 2250 | 24.87 | 5.9 | 8.3 | 21.9 | 4424493691 | 8.274E+10 | 7.079E+09 | 8.66E+09 | 8E+06 |
| r6.78 | S152 | | T2 | 91334 | Ribo100 | RiboCom | 2 | 167 | 52 | 19 | 3010 | 19.68 | 12.8 | 11 | 35.3 | 265469621 | 5.368E+10 | 9.992E+09 | 5.24E+09 | 7E+06 |
| r6.79 | S152 | | T3 | 62816 | Ribo100 | RiboCom | 2 | 167 | 52 | 19 | 4200 | 27.35 | 8.1 | 6.4 | 39.6 | 88489874 | 7.92E+10 | 4.166E+09 | 2.95E+08 | 5E+06 |
| r6.8 | S112 | | T4 | 44703 | Ribo50 | RiboCom | 2 | 170 | 54 | 19 | 2670 | 21.84 | 12.8 | 14 | 54.4 | 14748312 | 7.507E+10 | 3.355E+09 | 2.06E+08 | 4E+06 |
| r6.80 | S152 | | T4 | 46985 | Ribo100 | RiboCom | 2 | 167 | 52 | 19 | 1940 | 40.43 | 9.2 | 8.2 | 20.8 | 73741562 | 8.893E+10 | 1.18E+09 | 1.18E+08 | 4E+06 |
| r6.81 | S156 | | T1 | 65270 | Placebo | Placebo | 2 | 166 | 59 | 21 | 1520 | NA | 14.5 | 29.2 | 85.3 | 294966246 | 1.711E+10 | 1.807E+09 | 9.73E+08 | 1E+06 |
| r6.82 | S156 | | T2 | 91551 | Placebo | Placebo | 2 | 166 | 59 | 21 | 1920 | NA | 18.3 | 28.7 | 95.2 | 88489874 | 4.778E+10 | 2.507E+09 | 58993249 | 3E+06 |
| r6.83 | S156 | | T3 | 51519 | Placebo | Placebo | 2 | 166 | 59 | 21 | 1590 | NA | 9.4 | 44.4 | 51.9 | 339211183 | 6.032E+10 | 3.835E+09 | 5.31E+08 | 2E+06 |
| r6.84 | S156 | | T4 | 68130 | Placebo | Placebo | 2 | 166 | 59 | 21 | 1920 | 15.96 | 19.2 | 13.7 | 65.1 | 176979748 | 2.463E+10 | 2.102E+09 | 2.06E+08 | 3E+06 |
| r6.85 | S158 | | T1 | 76580 | Ribo100 | RiboCom | 2 | 169 | 55 | 19 | 3110 | 28.96 | 41 | 27.4 | 70.9 | 2418723218 | 1.031E+11 | 6.305E+09 | 1.17E+10 | 368708 |
| r6.86 | S158 | | T2 | 61307 | Ribo100 | RiboCom | 2 | 169 | 55 | 19 | 1810 | 28.28 | 40.7 | 31.4 | 77.3 | 221224685 | 7.802E+10 | 1.136E+10 | 58993249 | 2E+07 |
| r6.87 | S158 | | T3 | 93553 | Ribo100 | RiboCom | 2 | 169 | 55 | 19 | 4240 | 32.9 | 42.7 | 21.5 | 70.8 | 3156138833 | 4.13E+10 | 1.633E+10 | 7.09E+09 | 368708 |
| r6.88 | S158 | | T4 | 79781 | Ribo100 | RiboCom | 2 | 169 | 55 | 19 | 204000 | 30.65 | 27.9 | 25.5 | 45.8 | 811157177 | 4.351E+10 | 5.088E+09 | 5.44E+09 | 1E+06 |
| r6.89 | S159 | | T1 | 92563 | Placebo | Placebo | 2 | 163 | 61 | 23 | 3470 | 25.65 | 10.1 | 12.7 | 51.7 | 58993249 | 1.462E+11 | 4.015E+10 | 1.56E+09 | 1E+07 |
| r6.9 | S113 | | T1 | 114584 | Ribo50 | RiboCom | 2 | 176 | 64 | 21 | 1050 | 28.87 | 2.3 | 3.5 | 19.5 | 2109008660 | 1.032E+10 | 4.019E+09 | 2.23E+09 | 1E+06 |
| r6.90 | S159 | | T2 | 77992 | Placebo | Placebo | 2 | 163 | 61 | 23 | 2040 | 24.87 | 6.2 | 6.3 | 37.4 | 44244937 | 5.929E+10 | 1.412E+10 | 1.33E+08 | 737416 |
| r6.91 | S159 | | T3 | 132810 | Placebo | Placebo | 2 | 163 | 61 | 23 | 2760 | 26.11 | 37.7 | 22.1 | 70.2 | 2507213092 | 5.914E+10 | 8.554E+09 | 1.65E+09 | 737416 |
| r6.92 | S159 | | T4 | 106013 | Placebo | Placebo | 2 | 163 | 61 | 23 | 2630 | 27.15 | 13.5 | 10.3 | 50.9 | 678422366 | 1.68E+11 | 2.065E+09 | 8.11E+08 | 369000 |
| r6.93 | S160 | | T1 | 38471 | Placebo | Placebo | 1 | 172 | 64 | 22 | 2520 | 21.75 | 21.7 | 26.5 | 90 | 14748312 | 5.826E+10 | 1.549E+09 | 5.46E+08 | 369000 |
| r6.94 | S160 | | T2 | 82236 | Placebo | Placebo | 1 | 172 | 64 | 22 | 1890 | 28.84 | 17.4 | 15.8 | 92.1 | 44244937 | 3.377E+10 | 921769519 | 1.92E+08 | 368708 |
| r6.95 | S160 | | T3 | 94147 | Placebo | Placebo | 1 | 172 | 64 | 22 | 1560 | 18.06 | 21.7 | 36.1 | 96.7 | 162231435 | 1.681E+10 | 1.18E+09 | 2.51E+08 | 369000 |
| r6.96 | S160 | | T4 | 210548 | Placebo | Placebo | 1 | 172 | 64 | 22 | 2080 | 9.24 | 22.4 | 27.1 | 66 | 117986498 | 8.628E+10 | 921769519 | 1.18E+08 | 369000 |
| r6.97 | S161 | | T1 | 65552 | Ribo50 | RiboCom | 2 | 159 | 48 | 19 | 2380 | 21.78 | 21.1 | 25.4 | 83.8 | 294966246 | 7.168E+10 | 1.512E+09 | 2.27E+09 | 737000 |
| r7.1 | S161 | | T2 | 222726 | Ribo50 | RiboCom | 2 | 159 | 48 | 19 | 2510 | 20.8 | 18.4 | 19.9 | 73.7 | 1224109921 | 1.009E+11 | 6.969E+09 | 7.26E+09 | 369000 |
| r7.10 | S165 | | T3 | 126875 | Ribo50 | RiboCom | 2 | 174 | 68 | 23 | 2800 | 33.53 | 6.6 | 8.2 | 38.4 | 1297851483 | 4.012E+10 | 8.849E+09 | 1.09E+09 | 737416 |
| r7.11 | S165 | | T4 | 112528 | Ribo50 | RiboCom | 2 | 174 | 68 | 23 | 2620 | 27.71 | 12.1 | 10.2 | 47.4 | 280217934 | 9.203E+10 | 1.582E+10 | 3.67E+09 | 1E+06 |
| r7.12 | S166 | | T1 | 125620 | Ribo100 | RiboCom | 1 | 176 | 71 | 23 | 2690 | 24.03 | 7 | 8.9 | 27.8 | 176979748 | 2.448E+10 | 1.069E+09 | 1.47E+08 | 1E+06 |
| r7.13 | S166 | | T2 | 52951 | Ribo100 | RiboCom | 1 | 176 | 71 | 23 | 2230 | 21.41 | 16.2 | 24.4 | 52.9 | 88489874 | 3.466E+10 | 1.512E+09 | 1.53E+09 | 3E+06 |
| r7.14 | S166 | | T3 | 1748390 | Ribo100 | RiboCom | 1 | 176 | 71 | 23 | 6190 | 29.94 | 3.2 | 5.4 | 20.3 | 117986498 | 3.023E+10 | 774286396 | 5.6E+08 | 3E+06 |
| r7.15 | S166 | | T4 | 116913 | Ribo100 | RiboCom | 1 | 176 | 71 | 23 | 5600 | 64.81 | 8 | 9.6 | 30.1 | 44244937 | 3.982E+10 | 2.876E+09 | 1.92E+08 | 3E+06 |
| r7.16 | S167 | | T1 | 191698 | Ribo50 | RiboCom | 2 | 174 | 62 | 20 | 1550 | 22.26 | 8.9 | 10.3 | 31.8 | 988136924 | 7.787E+10 | 8.665E+09 | 1.78E+09 | 368708 |
| r7.17 | S167 | | T2 | 117044 | Ribo50 | RiboCom | 2 | 174 | 62 | 20 | 2640 | 29.07 | 11.4 | 12.7 | 36.6 | 2241743470 | 1.435E+11 | 1.453E+10 | 2.24E+09 | 2E+06 |
| r7.18 | S167 | | T3 | 144911 | Ribo50 | RiboCom | 2 | 174 | 62 | 20 | 49300 | 21.78 | 25.4 | 16 | 65.3 | 943891988 | 8.112E+10 | 7.632E+09 | 6.37E+09 | 5E+06 |
| r7.19 | S167 | | T4 | 64643 | Ribo50 | RiboCom | 2 | 174 | 62 | 20 | 4570 | 30.65 | 19.3 | 17.2 | 50.3 | 117986498 | 8.775E+10 | 2.286E+09 | 2.65E+08 | 737416 |
| r7.2 | S161 | | T3 | 446926 | Ribo50 | RiboCom | 2 | 159 | 48 | 19 | 5390 | 21.69 | 19.8 | 20.3 | 68.9 | 427701057 | 8.598E+10 | 2.249E+09 | 1.73E+09 | 368708 |
| r7.20 | S010 | | T1 | 281757 | Ribo100 | RiboCom | 2 | 169 | 63 | 22 | 1090 | 29.99 | 4.4 | 7.5 | 19.4 | NA | NA | NA | NA | NA |
| r7.21 | S010 | | T2 | 159844 | Ribo100 | RiboCom | 2 | 169 | 63 | 22 | 1510 | 36.9 | 4.6 | 7.7 | 23.1 | 103238186 | 2.197E+10 | 811157177 | 9.44E+08 | 1E+06 |
| r7.22 | S010 | | T3 | 165494 | Ribo100 | RiboCom | 2 | 169 | 63 | 22 | 2410 | 37.72 | 5 | 6.5 | 17.4 | 5899324922 | 3.023E+10 | 3.724E+09 | 6.27E+09 | 737416 |
| r7.23 | S010 | | T4 | 218792 | Ribo100 | RiboCom | 2 | 169 | 63 | 22 | 1780 | 38.93 | 8.2 | 12.3 | 32.3 | 929143675 | 5.708E+10 | 4.424E+09 | 3.73E+09 | 737416 |
| r7.3 | S161 | | T4 | 208178 | Ribo50 | RiboCom | 2 | 159 | 48 | 19 | 4040 | 18.5 | 27.6 | 24.5 | 96.8 | 929143675 | 3.318E+10 | 1.549E+09 | 7.08E+08 | 4E+06 |
| r7.4 | S164 | | T1 | 68276 | Ribo50 | RiboCom | 2 | 177 | 65 | 21 | 2240 | 23.28 | 9.2 | 8.4 | 49.7 | 545687555 | 7.492E+10 | 1.25E+10 | 5.46E+09 | 2E+06 |
| r7.5 | S164 | | T2 | 176349 | Ribo50 | RiboCom | 2 | 177 | 65 | 21 | 2140 | 33.02 | 9.1 | 10.9 | 40.2 | 722667303 | 7.448E+10 | 5.752E+09 | 3.82E+09 | 3E+06 |
| r7.6 | S164 | | T3 | 114840 | Ribo50 | RiboCom | 2 | 177 | 65 | 21 | 5890 | 33.46 | 22.9 | 12.9 | 47.3 | 1238858234 | 7.064E+10 | 8.96E+09 | 2.21E+09 | 2E+06 |
| r7.7 | S164 | | T4 | 47553 | Ribo50 | RiboCom | 2 | 177 | 65 | 21 | 3310 | 29.61 | 20 | 12.2 | 48.7 | 339211183 | 3.51E+10 | 4.351E+09 | 7.23E+08 | 5E+06 |
| r7.8 | S165 | | T1 | 160258 | Ribo50 | RiboCom | 2 | 174 | 68 | 23 | 1820 | 20.32 | 5.3 | 6.5 | 24.6 | 2005770473 | 5.368E+10 | 7.374E+09 | 1.09E+09 | 4E+06 |
| r7.9 | S165 | | T2 | 106186 | Ribo50 | RiboCom | 2 | 174 | 68 | 23 | 1600 | 16.39 | 1.6 | 1.9 | 8.5 | 1578069417 | 2.728E+10 | 2.212E+09 | 3.1E+08 | 5E+06 |
| r8.45 | S047 | | T1 | 56984 | Ribo50 | RiboCom | 2 | 165 | 61 | 22 | 3490 | NA | NA | NA | NA | 280217934 | 1.593E+10 | 1.733E+09 | 1.53E+09 | 3E+06 |
| r8.46 | S047 | | T2 | 145192 | Ribo50 | RiboCom | 2 | 165 | 61 | 22 | 3350 | NA | NA | NA | NA | 471945994 | 1.608E+10 | 737415615 | 2.06E+09 | 737416 |
| r8.47 | S047 | | T4 | 142537 | Ribo50 | RiboCom | 2 | 165 | 61 | 22 | 1990 | NA | NA | NA | NA | 368707807 | 1.401E+10 | 1.585E+09 | 2.06E+09 | 3E+06 |
| r8.48 | S048 | | T1 | 127338 | Ribo50 | RiboCom | 2 | 167 | 58 | 21 | 2130 | NA | 13 | 21.2 | 64.6 | 1755049164 | 1.681E+10 | 1.77E+09 | 3.79E+09 | 1E+06 |
| r8.49 | S048 | | T2 | 71300 | Ribo50 | RiboCom | 2 | 167 | 58 | 21 | 30300 | NA | 20.9 | 28.4 | 82.8 | 412952744 | 1.431E+10 | 2.065E+09 | 3.54E+08 | 6E+06 |
| r8.50 | S048 | | T4 | 145127 | Ribo50 | RiboCom | 2 | 167 | 58 | 21 | 2180 | NA | 11.4 | 13.6 | 62.8 | 1799294101 | 9.439E+09 | 1.327E+09 | 1.19E+09 | 368708 |
| r8.51 | S050 | | T1 | 184818 | Placebo | Placebo | 1 | 201 | 88 | 22 | 2330 | NA | 18.2 | 13.5 | 44.7 | 2639947903 | 3.702E+10 | 2.692E+09 | 3.78E+09 | 1E+06 |
| r8.52 | S050 | | T2 | 74136 | Placebo | Placebo | 1 | 201 | 88 | 22 | 2810 | NA | 19.8 | 11.2 | 31.9 | 2344981657 | 2.035E+10 | 4.904E+09 | 5.21E+09 | 368708 |
| r8.53 | S050 | | T3 | 138268 | Placebo | Placebo | 1 | 201 | 88 | 22 | 3210 | NA | 16.7 | 11.7 | 43.5 | 870150426 | 3.599E+10 | 5.346E+09 | 7.67E+08 | 368708 |
| r8.54 | S071 | | T1 | 69015 | Ribo50 | RiboCom | 2 | 157 | 49 | 20 | NA | NA | NA | NA | NA | NA | NA | NA | NA | NA |
| r8.55 | S076 | | T2 | 99282 | Ribo50 | RiboCom | 2 | 174 | 65 | 21 | 3260 | NA | 15.8 | 8.9 | 42.8 | 309714558 | 1.195E+10 | 1.254E+09 | 4.7E+09 | 3E+06 |
| r8.56 | S076 | | T4 | 183140 | Ribo50 | RiboCom | 2 | 174 | 65 | 21 | 2410 | NA | 21.4 | 19.3 | 63.9 | 221224685 | 5.486E+10 | 3.945E+09 | 1.27E+09 | 1E+06 |
| r8.57 | S080 | | T1 | 175359 | Ribo100 | RiboCom | 2 | 160 | 62 | 24 | 1900 | NA | 21.7 | 17.8 | 95.6 | 678422366 | 4.572E+09 | 1.18E+09 | 5.46E+08 | 2E+06 |
| r8.58 | S080 | | T2 | 158937 | Ribo100 | RiboCom | 2 | 160 | 62 | 24 | 2610 | NA | 15.6 | 15.1 | 75.4 | 6430264165 | 1.519E+10 | 1.807E+09 | 6.64E+08 | 2E+06 |
| r8.59 | S080 | | T4 | 77128 | Ribo100 | RiboCom | 2 | 160 | 62 | 24 | 3420 | NA | 19.5 | 20.1 | 65 | 1696055915 | 1.873E+10 | 1.327E+09 | 2.18E+09 | 2E+06 |
| r8.60 | S118 | | T1 | 74549 | Ribo100 | RiboCom | 2 | 159 | 60 | 24 | NA | NA | NA | NA | NA | 265469622 | 2.197E+10 | 2.397E+09 | 1.62E+08 | 1E+06 |
| r8.61 | S118 | | T3 | 184359 | Ribo100 | RiboCom | 2 | 159 | 60 | 24 | 4640 | NA | 11.4 | 8.2 | 42.8 | 1710804227 | 1.578E+10 | 1.733E+09 | 3.69E+08 | 2E+06 |
| r8.62 | S118 | | T4 | 96270 | Ribo100 | RiboCom | 2 | 159 | 60 | 24 | 3110 | NA | 16.8 | 14.1 | 52.8 | 412952744 | 2.463E+10 | 884898738 | 2.11E+09 | 3E+06 |
| r8.63 | S145 | | T1 | 209252 | Placebo | Placebo | 2 | 174 | 63 | 21 | 2550 | NA | 9.1 | 10.8 | 28.6 | 280217934 | 3.717E+10 | 2.249E+09 | 1.77E+08 | 2E+06 |
| r8.64 | S145 | | T2 | 46460 | Placebo | Placebo | 2 | 174 | 63 | 21 | 2490 | NA | 23.7 | 26.2 | 58.3 | 206476372 | 8.436E+10 | 2.323E+09 | 5.59E+09 | 3E+06 |
| r8.65 | S145 | | T4 | 143941 | Placebo | Placebo | 2 | 174 | 63 | 21 | 2450 | NA | 10.1 | 13 | 33.9 | 88489874 | 4.071E+10 | 3.134E+09 | 1.92E+08 | 9E+06 |
| r8.66 | S153 | | T2 | 140788 | Ribo50 | RiboCom | 2 | 161 | 62 | 24 | 2110 | NA | 12.8 | 15.5 | 59.4 | 73741561.5 | 6.386E+10 | 8.591E+09 | 4.72E+08 | 1E+06 |
| r8.67 | S153 | | T3 | 72749 | Ribo50 | RiboCom | 2 | 161 | 62 | 24 | 2720 | NA | 6 | 10.4 | 38.5 | 6105801294 | 4.572E+10 | 3.798E+09 | 7.33E+09 | 1E+06 |
| r8.68 | S153 | | T4 | 44737 | Ribo50 | RiboCom | 2 | 161 | 62 | 24 | 2120 | NA | 18 | 14.7 | 57.7 | 265469622 | 4.395E+10 | 3.281E+09 | 6.64E+08 | 1E+06 |
| r8.69 | S154 | | T1 | 100690 | Ribo100 | RiboCom | 2 | 171 | 60 | 20 | 2050 | NA | 41.2 | 14 | 90 | 1991022161 | 1.445E+10 | 1.733E+09 | 7.96E+08 | 368708 |
| r8.70 | S154 | | T3 | 88556 | Ribo100 | RiboCom | 2 | 171 | 60 | 20 | 7810 | NA | 39.8 | 22.1 | 87.3 | 294966246 | 4.174E+10 | 1.475E+09 | 29496625 | 4E+06 |
| r8.71 | S154 | | T4 | 100857 | Ribo100 | RiboCom | 2 | 171 | 60 | 20 | 2160 | NA | 16.4 | 9.2 | 39.8 | 4336003818 | 2.994E+10 | 2.802E+09 | 3.22E+09 | 2E+06 |
| r8.72 | S157 | | T2 | 95129 | Ribo50 | RiboCom | 2 | 173 | 57 | 19 | 1150 | NA | 14.5 | 29.2 | 85.3 | 516190931 | 5.752E+09 | 331837027 | 4.72E+08 | 1E+06 |
| r8.73 | S157 | | T3 | 141422 | Ribo50 | RiboCom | 2 | 173 | 57 | 19 | 2400 | NA | 18.3 | 28.7 | 95.2 | 501442619 | 1.652E+10 | 774286396 | 1.8E+09 | 2E+06 |
| r8.74 | S157 | | T4 | 99499 | Ribo50 | RiboCom | 2 | 173 | 57 | 19 | 1280 | NA | 9.4 | 44.4 | 51.9 | 6105801294 | 2.714E+10 | 1.217E+09 | 2.7E+09 | 737416 |
|  | | ***NA is missing data** | | | |  |  |  |  |  |  |  |  |  |  |  |  |  |  |  |
|  | | Gender (1 is Male, 2 is Female) | | | | |  |  |  |  |  |  |  |  |  |  |  |  |  |  |
