# Supplementary material for: Riboflavin Supplementation Promotes Butyrate Production in the Absence of Gross Compositional Changes in the Gut Microbiota
Source: Antioxid Redox Signal. 2023 Feb 14;38(4):282–97. doi: 10.1089/ars.2022.0033 (PMC9986023; doi:10.1089/ars.2022.0033)
Supplement: Supplemental data [file Suppl_TableS2.docx]

**Supplementary Table 2**. Shannon index and PD_whole_tree index of α diversity.

| **α diversity** | **Time** | **Placebo** | ***P-* value** | **Ribo50** | ***P-* value** | **Ribo100** | ***P-* value** |  |
| --- | --- | --- | --- | --- | --- | --- | --- | --- |
| **Shannon** | T1 | 6.34 ± 0.61 | *0.62* | 6.18 ± 0.59 | *0.96* | 6.36 ± 0.78 | *0.61* |  |
|  | T2 | 6.47 ± 0.41 |  | 6.25 ± 0.53 |  | 6.26 ± 0.61 |  |  |
|  | T3 | 6.29 ± 0.62 |  | 6.23 ± 0.58 |  | 6.15 ± 0.68 |  |  |
|  | T4 | 6.48 ± 0.46 |  | 6.19 ± 0.54 |  | 6.21 ± 0.72 |  |  |
| **PD_whole_tree** | T1 | 8.05 ± 1.04 | *0.67* | 7.74 ± 0.91 | *0.97* | 8.08 ± 1.25 | *0.84* |  |
|  | T2 | 8.26 ± 0.89 |  | 7.73 ± 1.00 |  | 7.92 ± 1.22 |  |  |
|  | T3 | 8.05 ± 1.16 |  | 7.59 ± 1.02 |  | 8.16 ± 1.45 |  |  |
|  | T4 | 8.27 ± 0.80 |  | 7.70 ± 1.10 |  | 7.93 ± 1.46 |  |  |
|  | | | | | | | | |

All indexed are presented as mean ± SD. **p*-values were calculated according to the Kruskal-Wallis Test within group.
