# Supplementary material for: Riboflavin Supplementation Promotes Butyrate Production in the Absence of Gross Compositional Changes in the Gut Microbiota
Source: Antioxid Redox Signal. 2023 Feb 14;38(4):282–97. doi: 10.1089/ars.2022.0033 (PMC9986023; doi:10.1089/ars.2022.0033)
Supplement: Supplemental data [file Suppl_FigS2.docx]

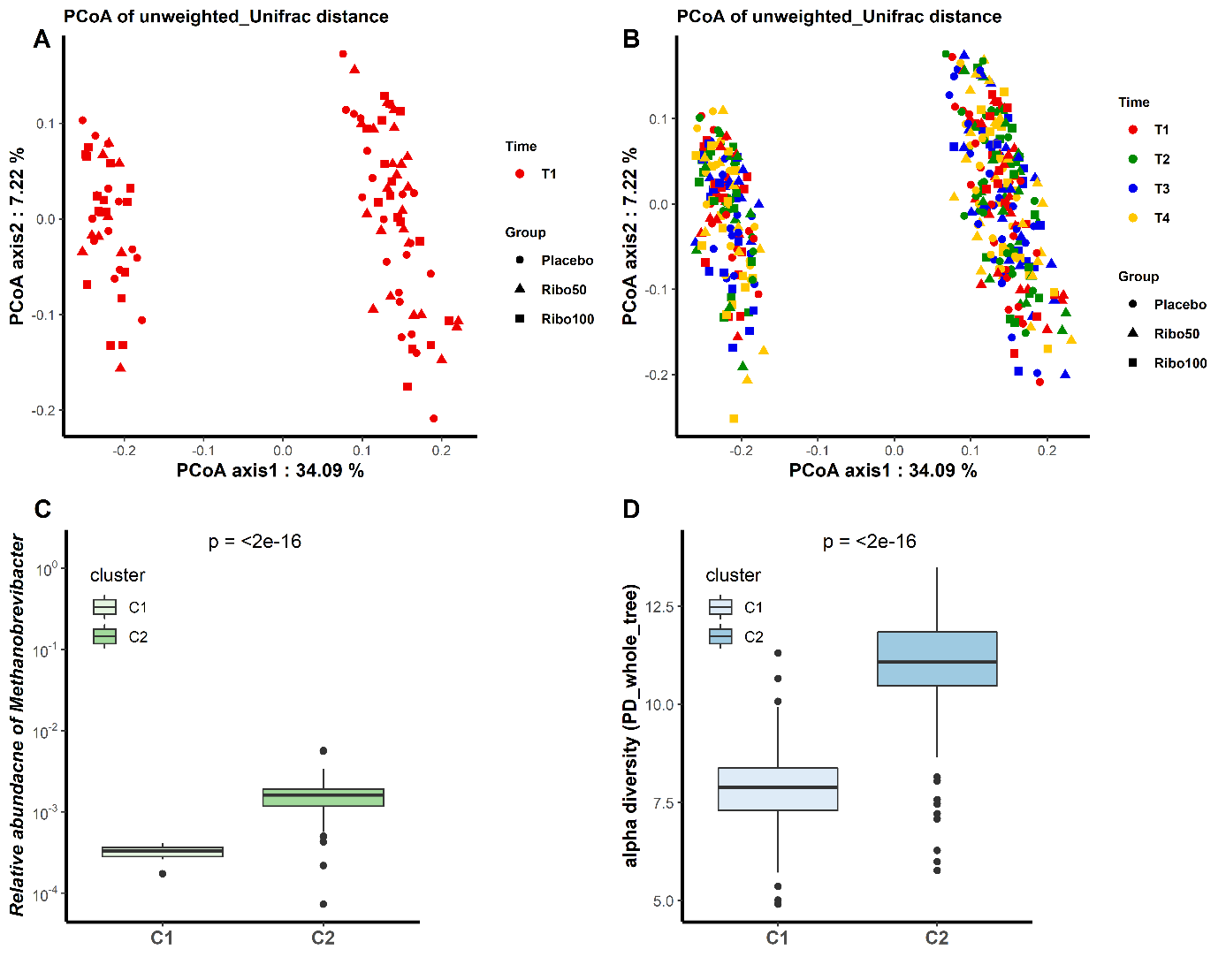
**Supplementary Figure 2.** PCoA separation of dataset based on unweighted_Unifrac distance. PCoA of unweighted_Unifrac distances on taxonomical composition at T1 (A) and all time points (B) for three groups. **C,** the relative abundance of *Methanobrevibacter* is significantly different between two clusters (*p* < 0.05, ANOVA test); **D**, PD_whole_tree index of α diversity is correlated to the separation and PD_whole_tree index of cluster 2 (C2) is significantly higher than that of cluster 1 (C1).
