# Supplementary figures and images for: Riboflavin Supplementation Promotes Butyrate Production in the Absence of Gross Compositional Changes in the Gut Microbiota
Source: Antioxid Redox Signal. 2023 Feb 14;38(4):282–97. doi: 10.1089/ars.2022.0033 (PMC9986023; doi:10.1089/ars.2022.0033)

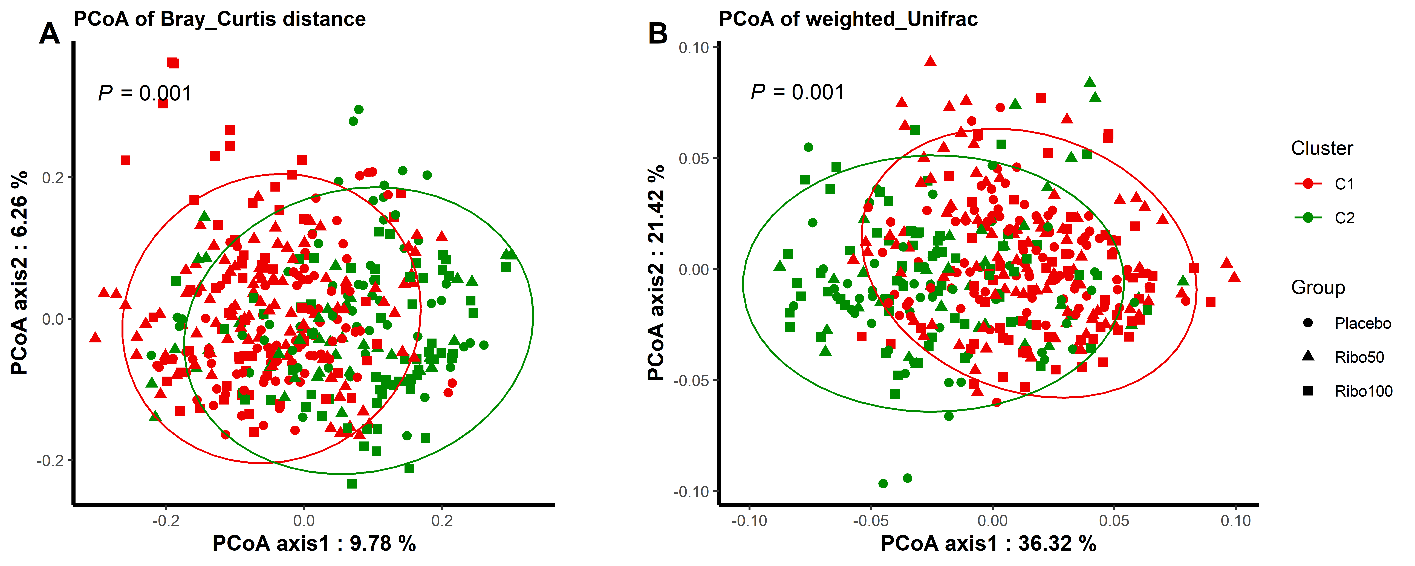
**Supplementary Figure 3.** PCoA of dataset based on Bray-Curtis (A) and weighted_Unifrac (B) distances.

Supplement: Supplemental data [file Suppl_FigS3.docx]
