# Supplementary material for: Riboflavin Supplementation Promotes Butyrate Production in the Absence of Gross Compositional Changes in the Gut Microbiota
Source: Antioxid Redox Signal. 2023 Feb 14;38(4):282–97. doi: 10.1089/ars.2022.0033 (PMC9986023; doi:10.1089/ars.2022.0033)

**Supplementary Figure 4.** The relation between butyrate (SCFAs) with top 30 most abundant bacterial genera. A, the correlation between butyrate concentration and the relative abundance of *Faecalibacterium* genus level (linear regression analysis). B, the Pearson correlation matrix between butyrate concentration and top 30 abundant bacterial genera (****p* < 0.001; ***p* < 0.01; **p*< 0.05), only genera with significant correlations are shown. C, The pairwise comparison of the relative abundances of *Faecalibacterium* between T2 and T3 among all groups (Wilcoxon signed-rank test). D, the pairwise comparison of the sum of relative abundances of genera (*Blautia*, *Roseburia*, *Lachnospiracea_incertae_sedis*, *Gemmiger*, *Faecalibacterium* and *Streptococcus*) which are most significantly (***) positively correlated with butyrate levels between T2 and T3 among all groups (Wilcoxon signed-rank test).


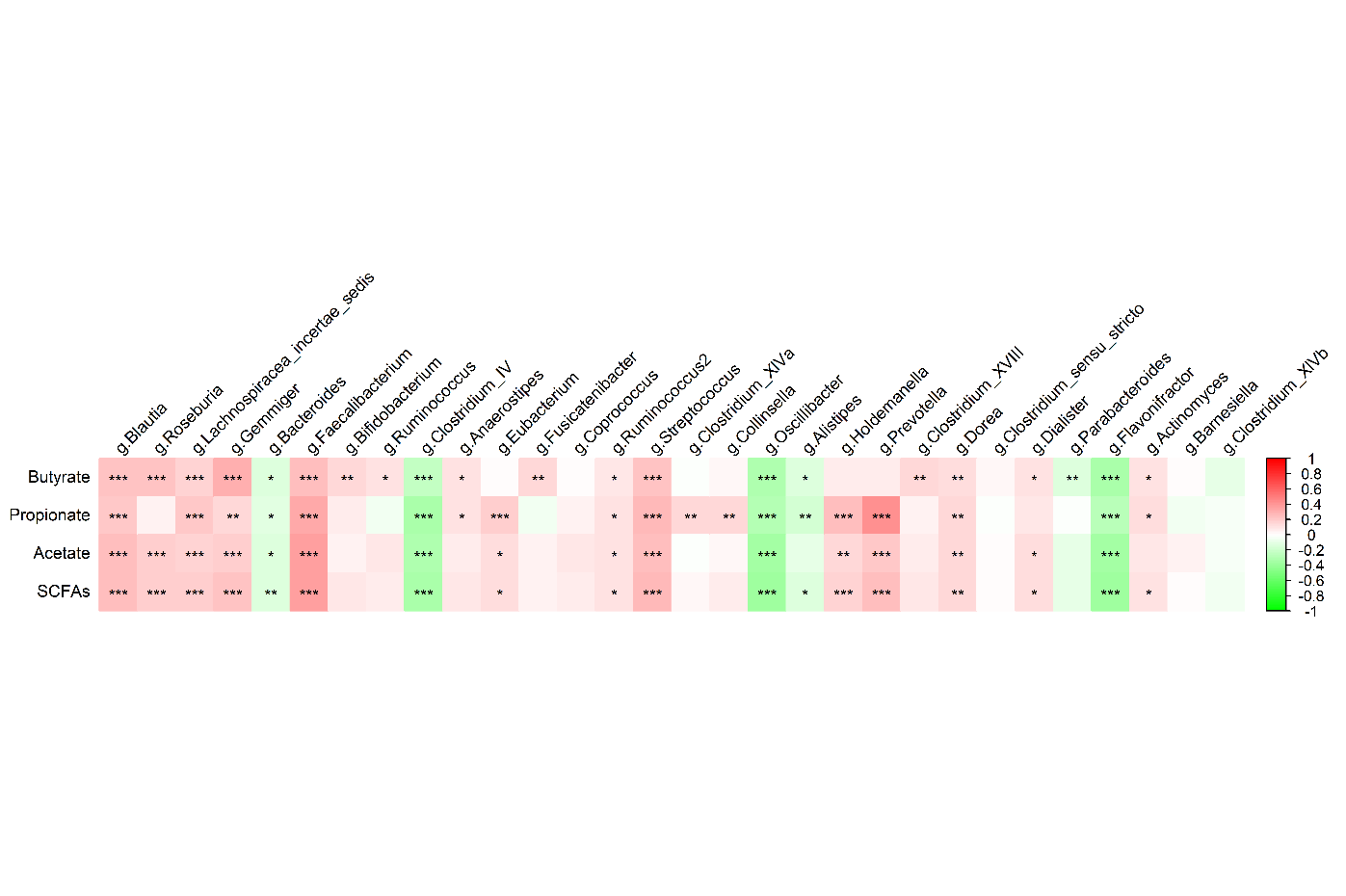

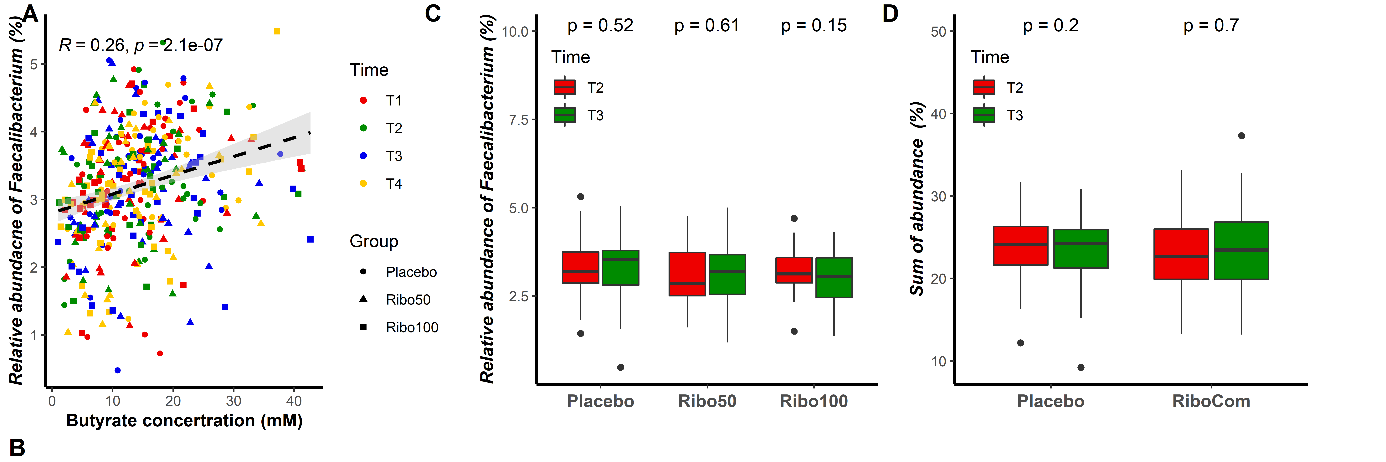

Supplement: Supplemental data [file Suppl_FigS4.docx]
