# Supplementary material for: Riboflavin Supplementation Promotes Butyrate Production in the Absence of Gross Compositional Changes in the Gut Microbiota
Source: Antioxid Redox Signal. 2023 Feb 14;38(4):282–97. doi: 10.1089/ars.2022.0033 (PMC9986023; doi:10.1089/ars.2022.0033)
Supplement: Supplemental data [file Suppl_FigS5.docx]

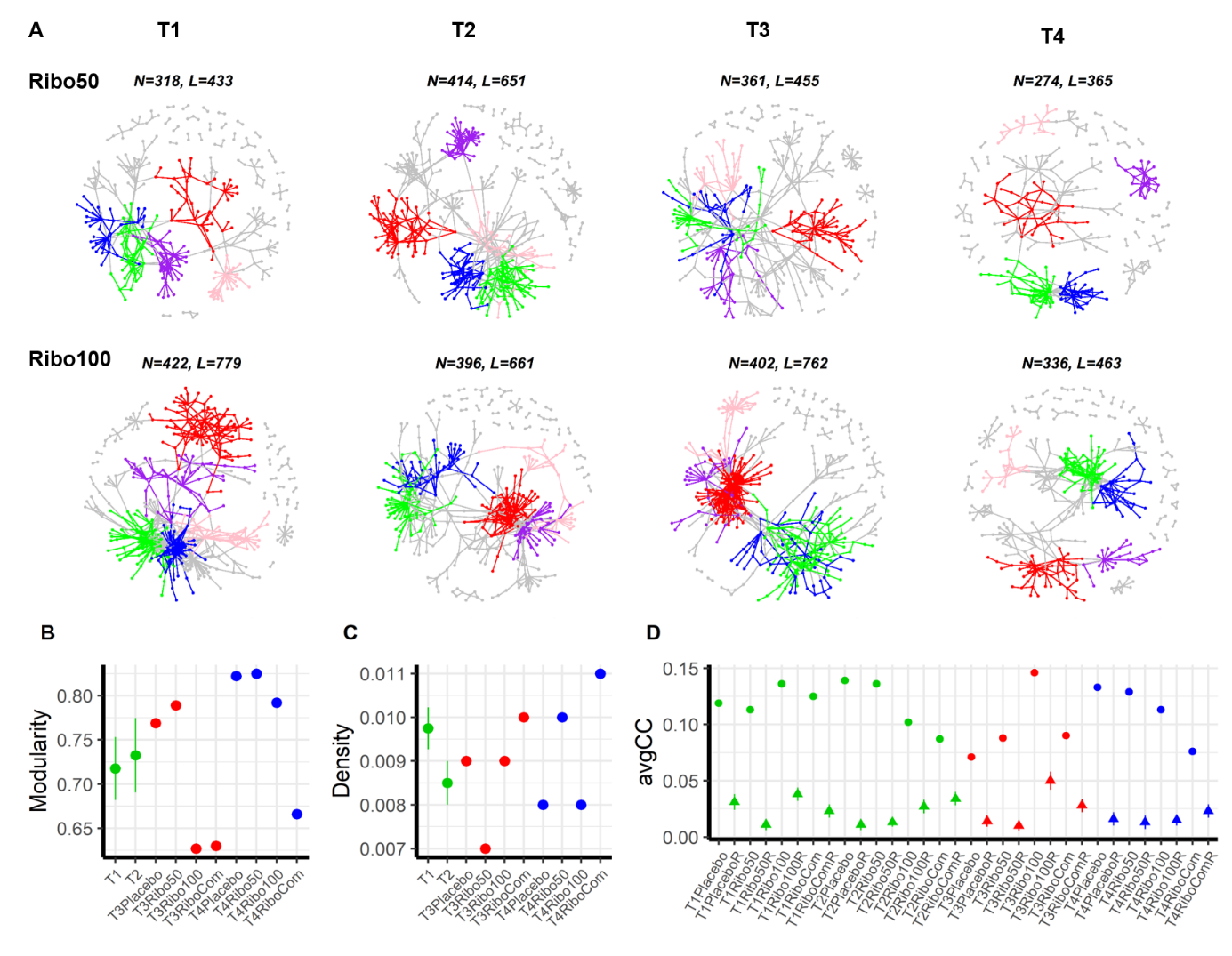
**Supplementary Figure 5.** Gut bacterial networks of Ribo50 and Ribo100 groups over all timepoints. A, visualization of MENs of Ribo50 (top) and Ribo100 groups (bottom) from T1 to T4. Top 5 large modules are shown in different colors, and smaller modules are shown in grey. Each network is shown based on Pearson correlations (RMT-threshold 0.67, FDR adjusted p < 0.05) between the abundances of bacterial ASVs of 36 Ribo50 samples and 33 Ribo100 samples. N, nodes; L, links. B-C, modularity (B) and density (C) are topological features of gut microbial networks. The baselines of T1 and T2 were calculated as means ± SE and the values of T3 and T4 networks shown as dots. D, average clustering coefficient (avgCC, each dot represents one network). The triangles represent random networks corresponding to each timepoint of each group. 100 random networks were generated by randomly rewiring all the links of a corresponding empirical network with the identical numbers of nodes and links. Values shown are the mean values and standard.
