# Supplementary material for: Riboflavin Supplementation Promotes Butyrate Production in the Absence of Gross Compositional Changes in the Gut Microbiota
Source: Antioxid Redox Signal. 2023 Feb 14;38(4):282–97. doi: 10.1089/ars.2022.0033 (PMC9986023; doi:10.1089/ars.2022.0033)
Supplement: Supplemental data [file Suppl_TableS3.docx]

**Supplementary Table 3**. Top 5 clusters of microbial networks.

| **Group** | **Time** | **Cluster** | **ASV_ID** | **ClosenessCentrality** | **ClusteringCoefficient** | **Degree** | **Eccentricity** | **NeighborhoodConnectivity** | **Node_Status** | **NumberOfUndirectedEdges** | **Radiality** | **Score** | **Stress** | **TopologicalCoefficient** | **Taxonomy** | | | | |
| --- | --- | --- | --- | --- | --- | --- | --- | --- | --- | --- | --- | --- | --- | --- | --- | --- | --- | --- | --- |
| Placebo | **T2** | Cluster 1 | ASV_3060 | 0.12 | 0.24 | 13 | 18 | 5.38 | Clustered | 13 | 0.61 | 4.00 | 82150 | 0.17 | *d:Bacteria;p:Firmicutes;c:Clostridia;o:Clostridiales;*  *f:Ruminococcaceae;g:Gemmiger;s:Gemmiger_formicilis* | | | | |
|  |  |  | ASV_3696 | 0.12 | 0.36 | 10 | 18 | 6.30 | Clustered | 10 | 0.61 | 4.00 | 37318 | 0.22 | *d:Bacteria;p:Firmicutes;c:Clostridia;o:Clostridiales;*  *f:Ruminococcaceae;g:Faecalibacterium;s:Faecalibacterium_prausnitzii* | | | | |
|  |  |  | ASV_5345 | 0.12 | 0.38 | 7 | 18 | 8.71 | Clustered | 7 | 0.61 | 4.00 | 215948 | 0.25 | *d:Bacteria;p:Firmicutes;c:Clostridia;o:Clostridiales;*  *f:Ruminococcaceae;g:Faecalibacterium;s:Faecalibacterium_prausnitzii* | | | | |
|  |  |  | ASV_1375 | 0.11 | 0.60 | 6 | 19 | 6.50 | Clustered | 6 | 0.56 | 4.00 | 120 | 0.43 | *d:Bacteria;p:Firmicutes;c:Clostridia;o:Clostridiales;*  *f:Ruminococcaceae;g:Faecalibacterium;s:Faecalibacterium_prausnitzii* | | | | |
|  |  |  | ASV_4268 | 0.11 | 1.00 | 4 | 19 | 9.00 | Seed | 4 | 0.56 | 4.00 | 0 | 0.60 | *d:Bacteria;p:Firmicutes;c:Clostridia;o:Clostridiales;*  *f:Ruminococcaceae;g:Faecalibacterium;s:Faecalibacterium_prausnitzii* | | | |  |
|  |  | Cluster 2 | ASV_29 | 0.09 | 1.00 | 2 | 21 | 4.50 | Clustered | 2 | 0.47 | 2.00 | 0 | 0.75 | *d:Bacteria;p:Firmicutes;c:Clostridia;o:Clostridiales;*  *f:Lachnospiraceae;g:Anaerostipes;s:Anaerostipes_hadrus* | | | |  |
|  |  |  | ASV_93 | 0.09 | 0.67 | 3 | 21 | 3.33 | Clustered | 3 | 0.47 | 1.67 | 2 | 0.56 | *d:Bacteria;p:Firmicutes;c:Clostridia;o:Clostridiales;*  *f:Lachnospiraceae;g:Anaerostipes;s:Anaerostipes_hadrus* | | | |  |
|  |  |  | ASV_3274 | 0.10 | 0.13 | 6 | 20 | 3.17 | Clustered | 6 | 0.52 | 1.67 | 98294 | 0.27 | *d:Bacteria;p:Firmicutes;c:Clostridia;o:Clostridiales;*  *f:Lachnospiraceae;g:Anaerostipes;* | | | |  |
|  |  |  | ASV_85 | 0.09 | 1.00 | 2 | 21 | 4.50 | Clustered | 2 | 0.47 | 2.00 | 0 | 0.75 | *d:Bacteria;p:Firmicutes;c:Clostridia;o:Clostridiales;*  *f:Lachnospiraceae;g:Anaerostipes;s:Anaerostipes_hadrus* | | | |  |
|  |  | Cluster 3 | ASV_4571 | 0.13 | 0.14 | 9 | 16 | 4.33 | Clustered | 9 | 0.65 | 2.70 | 9162 | 0.20 | *d:Bacteria;p:Firmicutes;c:Clostridia;o:Clostridiales;*  *f:Lachnospiraceae* | | | |  |
|  |  |  | ASV_1770 | 0.13 | 0.47 | 6 | 16 | 6.50 | Clustered | 6 | 0.64 | 2.40 | 4328 | 0.33 | *d:Bacteria;p:Firmicutes;c:Clostridia;o:Clostridiales;*  *f:Lachnospiraceae* | | | |  |
|  |  |  | ASV_5650 | 0.13 | 0.50 | 5 | 16 | 6.00 | Seed | 5 | 0.64 | 2.70 | 2842 | 0.36 | *d:Bacteria;p:Firmicutes;c:Clostridia;o:Clostridiales;*  *f:Lachnospiraceae;g:Roseburia* | | | |  |
|  |  | Cluster 4 | ASV_5216 | 0.15 | 0.19 | 16 | 15 | 6.06 | Clustered | 16 | 0.69 | 2.11 | 389966 | 0.16 | *d:Bacteria;p:Firmicutes;c:Clostridia;o:Clostridiales;*  *f:Lachnospiraceae* | | | |  |
|  |  |  | ASV_2476 | 0.13 | 0.48 | 7 | 16 | 7.57 | Clustered | 7 | 0.64 | 2.14 | 6714 | 0.30 | *d:Bacteria;p:Firmicutes;c:Clostridia;o:Clostridiales;*  *f:Lachnospiraceae;g:Roseburia* | | | |  |
|  |  |  | ASV_3840 | 0.14 | 0.12 | 15 | 16 | 6.20 | Clustered | 15 | 0.67 | 1.93 | 557280 | 0.13 | *d:Bacteria;p:Firmicutes;c:Clostridia;o:Clostridiales;*  *f:Lachnospiraceae;g:Blautia* | | | |  |
|  |  |  | ASV_3287 | 0.13 | 1.00 | 2 | 16 | 15.50 | Clustered | 2 | 0.65 | 2.00 | 0 | 0.65 | *d:Bacteria;p:Firmicutes;c:Clostridia;o:Clostridiales;*  *f:Lachnospiraceae* | | | |  |
|  |  |  | ASV_5074 | 0.13 | 1.00 | 2 | 16 | 11.50 | Clustered | 2 | 0.64 | 2.00 | 0 | 0.68 | *d:Bacteria;p:Firmicutes;c:Clostridia;o:Clostridiales;*  *f:Lachnospiraceae* | | | |  |
|  |  | Cluster 5 | ASV_28 | 1.00 | 0.07 | 6 | 1 | 1.33 | Clustered | 6 | 1.00 | 2.00 | 28 | 0.33 | *d:Bacteria;p:Firmicutes;c:Clostridia;o:Clostridiales;*  *f:Ruminococcaceae;g:Faecalibacterium;s:Faecalibacterium_prausnitzii* | | | |  |
|  |  |  | ASV_53 | 0.60 | 1.00 | 2 | 2 | 4.00 | Clustered | 2 | 0.89 | 2.00 | 0 | 0.67 | *d:Bacteria;p:Firmicutes;c:Clostridia;o:Clostridiales;*  *f:Eubacteriaceae;g:Eubacterium;s:Eubacterium_desmolans* | | | |  |
|  |  |  | ASV_5624 | 0.60 | 1.00 | 2 | 2 | 4.00 | Seed | 2 | 0.89 | 2.00 | 0 | 0.67 | *d:Bacteria;p:Firmicutes;c:Clostridia;o:Clostridiales;*  *f:Eubacteriaceae;g:Eubacterium;s:Eubacterium_desmolans* | | | |  |
|  | T3 | Cluster 1 | ASV_3840 | 0.25 | 0.25 | 8 | 10 | 9.50 | Clustered | 8 | 0.86 | 2.70 | 58534 | 0.19 | *d:Bacteria;p:Firmicutes;c:Clostridia;o:Clostridiales;*  *f:Lachnospiraceae;g:Blautia* | | | |  |
|  |  |  | ASV_2476 | 0.24 | 0.13 | 13 | 10 | 5.77 | Clustered | 13 | 0.85 | 2.40 | 64222 | 0.13 | *d:Bacteria;p:Firmicutes;c:Clostridia;o:Clostridiales;*  *f:Lachnospiraceae;g:Roseburia* | | | |  |
|  |  |  | ASV_2998 | 0.23 | 0.32 | 8 | 11 | 8.63 | Clustered | 8 | 0.84 | 2.40 | 6312 | 0.20 | *d:Bacteria;p:Firmicutes;c:Clostridia;o:Clostridiales;*  *f:Lachnospiraceae* | | | |  |
|  |  |  | ASV_4404 | 0.21 | 0.30 | 5 | 11 | 7.60 | Seed | 5 | 0.82 | 3.00 | 5574 | 0.33 | *d:Bacteria;p:Firmicutes;c:Clostridia;o:Clostridiales;*  *f:Lachnospiraceae* | | | |  |
|  |  | Cluster 2 | ASV_2 | 0.21 | 0.20 | 6 | 12 | 13.50 | Clustered | 6 | 0.82 | 1.40 | 18780 | 0.39 | *d:Bacteria;p:Firmicutes;c:Clostridia;o:Clostridiales;*  *f:Ruminococcaceae;g:Gemmiger;s:Gemmiger_formicilis* | | | |  |
|  |  |  | ASV_7 | 0.21 | 0.20 | 6 | 12 | 13.67 | Clustered | 6 | 0.82 | 1.40 | 26710 | 0.38 | *d:Bacteria;p:Firmicutes;c:Clostridia;o:Clostridiales;*  *f:Lachnospiraceae;g:Fusicatenibacter;s:Fusicatenibacter_saccharivorans* | | | |  |
|  |  |  | ASV_11 | 0.21 | 0.30 | 5 | 12 | 15.80 | Clustered | 5 | 0.82 | 1.40 | 8708 | 0.47 | *d:Bacteria;p:Firmicutes;c:Clostridia;o:Clostridiales;*  *f:Ruminococcaceae;g:Faecalibacterium;s:Faecalibacterium_prausnitzii* | | | |  |
|  |  |  | ASV_13 | 0.22 | 0.32 | 16 | 12 | 9.63 | Seed | 16 | 0.83 | 1.70 | 16796 | 0.16 | *d:Bacteria;p:Firmicutes;c:Clostridia;o:Clostridiales;*  *f:Ruminococcaceae;g:Faecalibacterium;s:Faecalibacterium_prausnitzii* | | | |  |
|  |  |  | ASV_53 | 0.21 | 0.30 | 5 | 12 | 15.80 | Clustered | 5 | 0.82 | 1.40 | 8708 | 0.47 | *d:Bacteria;p:Firmicutes;c:Clostridia;o:Clostridiales;*  *f:Eubacteriaceae;g:Eubacterium;s:Eubacterium_desmolans* | | |  |  |
|  |  |  | ASV_3854 | 0.21 | 0.30 | 5 | 12 | 14.20 | Clustered | 5 | 0.82 | 1.40 | 10218 | 0.42 | *d:Bacteria;p:Firmicutes;c:Clostridia;o:Clostridiales;*  *f:Ruminococcaceae;g:Faecalibacterium;s:Faecalibacterium_prausnitzii* | | |  |  |
|  |  |  | ASV_25 | 0.21 | 0.14 | 17 | 13 | 7.65 | Clustered | 17 | 0.82 | 1.67 | 31752 | 0.16 | *d:Bacteria;p:Firmicutes;c:Clostridia;o:Clostridiales;*  *f:Ruminococcaceae* | | |  |  |
|  |  |  | ASV_89 | 0.19 | 0.67 | 3 | 13 | 16.33 | Clustered | 3 | 0.79 | 1.67 | 416 | 0.71 | *d:Bacteria;p:Firmicutes;c:Clostridia;o:Clostridiales;*  *f:Ruminococcaceae;g:Faecalibacterium;s:Faecalibacterium_prausnitzii* | | |  |  |
|  |  | Cluster 3 | ASV_4781 | 0.67 | 0.33 | 3 | 2 | 3.00 | Clustered | 3 | 0.88 | 2.00 | 8 | 0.53 | *d:Bacteria;p:Actinobacteria;c:Actinobacteria;o:Coriobacteriales;*  *f:Coriobacteriaceae;g:Collinsella;s:Collinsella_aerofaciens* | | |  |  |
|  |  |  | ASV_4665 | 0.75 | 0.17 | 4 | 2 | 2.25 | Clustered | 4 | 0.92 | 2.00 | 18 | 0.44 | *d:Bacteria;p:Actinobacteria;c:Actinobacteria;o:Coriobacteriales;*  *f:Coriobacteriaceae;g:Collinsella;s:Collinsella_aerofaciens* | | |  |  |
|  |  |  | ASV_5206 | 0.67 | 0.33 | 3 | 2 | 2.67 | Seed | 3 | 0.88 | 2.00 | 10 | 0.47 | *d:Bacteria;p:Actinobacteria;c:Actinobacteria;o:Coriobacteriales;*  *f:Coriobacteriaceae;g:Collinsella;s:Collinsella_aerofaciens* | | |  |  |
|  |  | Cluster 4 | ASV_29 | 0.10 | 0.17 | 4 | 14 | 2.25 | Clustered | 4 | 0.59 | 2.00 | 13256 | 0.35 | *d:Bacteria;p:Firmicutes;c:Clostridia;o:Clostridiales;*  *f:Lachnospiraceae;g:Anaerostipes;s:Anaerostipes_hadrus* | | |  |  |
|  |  |  | ASV_78 | 0.09 | 1.00 | 2 | 15 | 3.00 | Clustered | 2 | 0.54 | 2.00 | 0 | 0.75 | *d:Bacteria;p:Firmicutes;c:Clostridia;o:Clostridiales;*  *f:Lachnospiraceae;g:Anaerostipes;s:Anaerostipes_hadrus* | | |  |  |
|  |  |  | ASV_4978 | 0.09 | 1.00 | 2 | 15 | 3.00 | Seed | 2 | 0.54 | 2.00 | 0 | 0.75 | *d:Bacteria;p:Firmicutes;c:Clostridia;o:Clostridiales;*  *f:Lachnospiraceae;g:Anaerostipes;s:Anaerostipes_hadrus* | | |  |  |
|  |  | Cluster 5 | ASV_6 | 0.67 | 0.33 | 3 | 2 | 2.67 | Clustered | 3 | 0.88 | 2.00 | 10 | 0.47 | *d:Bacteria;p:Firmicutes;c:Clostridia;o:Clostridiales;*  *f:Ruminococcaceae;g:Gemmiger;s:Gemmiger_formicilis* | | |  |  |
|  |  |  | ASV_2442 | 0.75 | 0.17 | 4 | 2 | 2.00 | Clustered | 4 | 0.92 | 2.00 | 18 | 0.38 | *d:Bacteria;p:Firmicutes;c:Clostridia;o:Clostridiales;*  *f:Ruminococcaceae;g:Gemmiger;s:Gemmiger_formicilis* | | |  |  |
|  |  |  | ASV_5666 | 0.67 | 0.33 | 3 | 2 | 2.67 | Seed | 3 | 0.88 | 2.00 | 10 | 0.47 | *d:Bacteria;p:Firmicutes;c:Clostridia;o:Clostridiales;*  *f:Ruminococcaceae;g:Gemmiger;s:Gemmiger_formicilis* | | |  |  |
| RiboCom | T2 | Cluster 1 | ASV_21 | 0.19 | 0.16 | 11 | 9 | 4.36 | Clustered | 11 | 0.91 | 2.70 | 50720 | 0.23 | *d:Bacteria;p:Firmicutes;c:Clostridia;o:Clostridiales;*  *f:Lachnospiraceae;g:Lachnospiracea_incertae_sedis;s:Eubacterium_hallii* | | |  |  |
|  |  |  | ASV_29 | 0.19 | 0.29 | 8 | 9 | 5.38 | Clustered | 8 | 0.91 | 2.70 | 25484 | 0.26 | *d:Bacteria;p:Firmicutes;c:Clostridia;o:Clostridiales;*  *f:Lachnospiraceae;g:Anaerostipes;s:Anaerostipes_hadrus* | | |  |  |
|  |  |  | ASV_93 | 0.18 | 0.40 | 5 | 9 | 6.20 | Seed | 5 | 0.90 | 3.00 | 3118 | 0.40 | *d:Bacteria;p:Firmicutes;c:Clostridia;o:Clostridiales;*  *f:Lachnospiraceae;g:Anaerostipes;s:Anaerostipes_hadrus* | | |  |  |
|  |  |  | ASV_3274 | 0.22 | 0.43 | 7 | 8 | 7.14 | Clustered | 7 | 0.92 | 2.40 | 34566 | 0.29 | *d:Bacteria;p:Firmicutes;c:Clostridia;o:Clostridiales;*  *f:Lachnospiraceae;g:Anaerostipes* | | |  |  |
|  |  | Cluster 2 | ASV_5 | 0.13 | 1.00 | 3 | 12 | 4.00 | Clustered | 3 | 0.86 | 3.00 | 0 | 0.67 | *d:Bacteria;p:Actinobacteria;c:Actinobacteria;o:Coriobacteriales;*  *f:Coriobacteriaceae;g:Collinsella;s:Collinsella_aerofaciens* | | |  |  |
|  |  |  | ASV_4781 | 0.15 | 0.33 | 6 | 11 | 2.83 | Clustered | 6 | 0.88 | 3.00 | 18652 | 0.40 | *d:Bacteria;p:Actinobacteria;c:Actinobacteria;o:Coriobacteriales;*  *f:Coriobacteriaceae;g:Collinsella;s:Collinsella_aerofaciens* | | |  |  |
|  |  |  | ASV_4609 | 0.13 | 1.00 | 3 | 12 | 4.00 | Clustered | 3 | 0.86 | 3.00 | 0 | 0.67 | *d:Bacteria;p:Actinobacteria;c:Actinobacteria;o:Coriobacteriales;*  *f:Coriobacteriaceae;g:Collinsella;s:Collinsella_aerofaciens* | | |  |  |
|  |  |  | ASV_4377 | 0.13 | 1.00 | 3 | 12 | 4.00 | Seed | 3 | 0.86 | 3.00 | 0 | 0.67 | *d:Bacteria;p:Firmicutes;c:Clostridia;o:Clostridiales;*  *f:Lachnospiraceae;g:Blautia;s:Blautia_wexlerae* | | |  |  |
|  |  | Cluster 3 | ASV_1 | 0.23 | 0.12 | 16 | 9 | 4.63 | Clustered | 16 | 0.93 | 3.00 | 80902 | 0.12 | *d:Bacteria;p:Firmicutes;c:Clostridia;o:Clostridiales;*  *f:Lachnospiraceae* | | |  |  |
|  |  |  | ASV_2476 | 0.21 | 0.12 | 14 | 9 | 3.93 | Clustered | 14 | 0.92 | 3.00 | 23040 | 0.14 | *d:Bacteria;p:Firmicutes;c:Clostridia;o:Clostridiales;*  *f:Lachnospiraceae;g:Roseburia* | | |  |  |
|  |  |  | ASV_1829 | 0.19 | 0.50 | 5 | 10 | 8.20 | Clustered | 5 | 0.91 | 3.00 | 6564 | 0.37 | *d:Bacteria;p:Firmicutes;c:Clostridia;o:Clostridiales;*  *f:Lachnospiraceae;g:Roseburia* | | |  |  |
|  |  |  | ASV_4571 | 0.19 | 0.50 | 5 | 10 | 8.20 | Seed | 5 | 0.91 | 3.00 | 6564 | 0.37 | *d:Bacteria;p:Firmicutes;c:Clostridia;o:Clostridiales;*  *f:Lachnospiraceae* | | |  |  |
|  |  | Cluster 4 | ASV_3553 | 0.21 | 0.33 | 3 | 9 | 7.33 | Clustered | 3 | 0.92 | 2.00 | 3792 | 0.39 | *d:Bacteria;p:Firmicutes;c:Clostridia;o:Clostridiales;*  *f:Lachnospiraceae;g:Blautia;s:Blautia_faecis* | | |  |  |
|  |  |  | ASV_1927 | 0.20 | 0.67 | 3 | 9 | 3.67 | Clustered | 3 | 0.91 | 1.67 | 366 | 0.52 | *d:Bacteria;p:Firmicutes;c:Clostridia;o:Clostridiales;*  *f:Lachnospiraceae;g:Blautia;s:Blautia_faecis* | | |  |  |
|  |  |  | ASV_4432 | 0.24 | 0.20 | 5 | 8 | 3.40 | Clustered | 5 | 0.93 | 1.67 | 9170 | 0.33 | *d:Bacteria;p:Firmicutes;c:Clostridia;o:Clostridiales;*  *f:Lachnospiraceae;g:Blautia;s:Blautia_faecis* | |  |  |  |
|  |  |  | ASV_3394 | 0.19 | 0.33 | 3 | 9 | 4.00 | Seed | 3 | 0.91 | 2.00 | 432 | 0.61 | *d:Bacteria;p:Firmicutes;c:Clostridia;o:Clostridiales;*  *f:Lachnospiraceae* | |  |  |  |
|  |  | Cluster 5 | ASV_4 | 0.18 | 1.00 | 2 | 9 | 6.00 | Clustered | 2 | 0.90 | 2.00 | 0 | 0.67 | *d:Bacteria;p:Firmicutes;c:Clostridia;o:Clostridiales;*  *f:Lachnospiraceae;g:Blautia;s:Blautia_wexlerae* | |  |  |  |
|  |  |  | ASV_4147 | 0.23 | 0.07 | 8 | 8 | 2.50 | Clustered | 8 | 0.93 | 1.67 | 37630 | 0.19 | *d:Bacteria;p:Firmicutes;c:Clostridia;o:Clostridiales;*  *f:Lachnospiraceae* | |  |  |  |
|  |  |  | ASV_5334 | 0.19 | 0.33 | 4 | 9 | 3.25 | Clustered | 4 | 0.90 | 1.67 | 3152 | 0.38 | *d:Bacteria;p:Firmicutes;c:Clostridia;o:Clostridiales;*  *f:Lachnospiraceae;g:Blautia;s:Blautia_wexlerae* | |  |  |  |
|  |  |  | ASV_4719 | 0.18 | 1.00 | 2 | 9 | 6.00 | Seed | 2 | 0.90 | 2.00 | 0 | 0.67 | *d:Bacteria;p:Firmicutes;c:Clostridia;o:Clostridiales;*  *f:Lachnospiraceae;g:Blautia;s:Blautia_wexlerae* | |  |  |  |
|  | T3 | Cluster 1 | ASV_1 | 0.23 | 0.14 | 24 | 9 | 7.29 | Clustered | 24 | 0.90 | 5.79 | 169528 | 0.12 | *d:Bacteria;p:Firmicutes;c:Clostridia;o:Clostridiales;*  *f:Lachnospiraceae* | |  |  |  |
|  |  |  | ASV_7 | 0.22 | 0.20 | 19 | 9 | 7.68 | Clustered | 19 | 0.90 | 5.79 | 61182 | 0.15 | *d:Bacteria;p:Firmicutes;c:Clostridia;o:Clostridiales;*  *f:Lachnospiraceae;g:Fusicatenibacter;s:Fusicatenibacter_saccharivorans* | |  |  |  |
|  |  |  | ASV_14 | 0.22 | 0.33 | 13 | 9 | 10.08 | Clustered | 13 | 0.90 | 6.00 | 39372 | 0.19 | *d:Bacteria;p:Firmicutes;c:Clostridia;o:Clostridiales;*  *f:Lachnospiraceae;g:Blautia;s:Blautia_luti* | |  |  |  |
|  |  |  | ASV_3840 | 0.27 | 0.20 | 18 | 8 | 8.56 | Clustered | 18 | 0.92 | 5.79 | 272622 | 0.14 | *d:Bacteria;p:Firmicutes;c:Clostridia;o:Clostridiales;*  *f:Lachnospiraceae;g:Blautia* | |  |  |  |
|  |  |  | ASV_5216 | 0.24 | 0.21 | 18 | 8 | 8.50 | Clustered | 18 | 0.91 | 5.79 | 71466 | 0.15 | *d:Bacteria;p:Firmicutes;c:Clostridia;o:Clostridiales;*  *f:Lachnospiraceae* | |  |  |  |
|  |  |  | ASV_2476 | 0.22 | 0.28 | 16 | 9 | 9.00 | Clustered | 16 | 0.90 | 5.79 | 8756 | 0.17 | *d:Bacteria;p:Firmicutes;c:Clostridia;o:Clostridiales;*  *f:Lachnospiraceae;g:Roseburia* | |  |  |  |
|  |  |  | ASV_5650 | 0.22 | 0.67 | 9 | 9 | 13.56 | Clustered | 9 | 0.90 | 5.79 | 4616 | 0.28 | *d:Bacteria;p:Firmicutes;c:Clostridia;o:Clostridiales;*  *f:Lachnospiraceae;g:Roseburia* | |  |  |  |
|  |  |  | ASV_4770 | 0.22 | 0.61 | 8 | 9 | 13.88 | Seed | 8 | 0.90 | 6.00 | 5896 | 0.29 | *d:Bacteria;p:Firmicutes;c:Clostridia;o:Clostridiales;*  *f:Lachnospiraceae;g:Roseburia* | |  |  |  |
|  |  | Cluster 2 | ASV_21 | 0.17 | 0.67 | 4 | 10 | 4.75 | Clustered | 4 | 0.86 | 3.00 | 8 | 0.48 | *d:Bacteria;p:Firmicutes;c:Clostridia;o:Clostridiales;*  *f:Lachnospiraceae;g:Lachnospiracea_incertae_sedis;s:Eubacterium_hallii* | |  |  |  |
|  |  |  | ASV_1674 | 0.20 | 0.19 | 9 | 9 | 3.11 | Clustered | 9 | 0.88 | 3.00 | 26640 | 0.25 | *d:Bacteria;p:Firmicutes;c:Clostridia;o:Clostridiales;*  *f:Lachnospiraceae;g:Lachnospiracea_incertae_sedis;s:Eubacterium_hallii* |  |  |  |  |
|  |  |  | ASV_5557 | 0.17 | 1.00 | 3 | 10 | 5.33 | Clustered | 3 | 0.86 | 3.00 | 0 | 0.59 | *d:Bacteria;p:Firmicutes;c:Clostridia;o:Clostridiales;*  *f:Lachnospiraceae;g:Lachnospiracea_incertae_sedis;s:Eubacterium_hallii* |  |  |  |  |
|  |  |  | ASV_3041 | 0.17 | 1.00 | 3 | 10 | 5.33 | Seed | 3 | 0.86 | 3.00 | 0 | 0.59 | *d:Bacteria;p:Firmicutes;c:Clostridia;o:Clostridiales;*  *f:Lachnospiraceae;g:Lachnospiracea_incertae_sedis;s:Eubacterium_hallii* |  |  |  |  |
|  |  | Cluster 3 | ASV_5 | 0.16 | 0.33 | 3 | 11 | 4.33 | Clustered | 3 | 0.85 | 2.00 | 11908 | 0.44 | *d:Bacteria;p:Actinobacteria;c:Actinobacteria;o:Coriobacteriales;*  *f:Coriobacteriaceae;g:Collinsella;s:Collinsella_aerofaciens* |  |  |  |  |
|  |  |  | ASV_4665 | 0.19 | 0.10 | 7 | 10 | 2.14 | Clustered | 7 | 0.88 | 1.67 | 107056 | 0.22 | *d:Bacteria;p:Actinobacteria;c:Actinobacteria;o:Coriobacteriales;*  *f:Coriobacteriaceae;g:Collinsella;s:Collinsella_aerofaciens* |  |  |  |  |
|  |  |  | ASV_4781 | 0.16 | 0.20 | 5 | 11 | 2.80 | Clustered | 5 | 0.85 | 1.67 | 23814 | 0.30 | *d:Bacteria;p:Actinobacteria;c:Actinobacteria;o:Coriobacteriales;*  *f:Coriobacteriaceae;g:Collinsella;s:Collinsella_aerofaciens* |  |  |  |  |
|  |  |  | ASV_4375 | 0.16 | 1.00 | 2 | 11 | 6.00 | Seed | 2 | 0.85 | 2.00 | 0 | 0.67 | *d:Bacteria;p:Actinobacteria;c:Actinobacteria;o:Coriobacteriales;*  *f:Coriobacteriaceae;g:Collinsella;s:Collinsella_aerofaciens* |  |  |  |  |
|  |  | Cluster 4 | ASV_5570 | 0.23 | 0.07 | 6 | 8 | 10.50 | Clustered | 6 | 0.90 | 2.00 | 45548 | 0.23 | *d:Bacteria;p:Firmicutes;c:Clostridia;o:Clostridiales;*  *f:Lachnospiraceae;g:Roseburia* |  |  |  |  |
|  |  |  | ASV_11 | 0.19 | 0.20 | 5 | 9 | 4.60 | Clustered | 5 | 0.88 | 1.67 | 10646 | 0.25 | *d:Bacteria;p:Firmicutes;c:Clostridia;o:Clostridiales;*  *f:Ruminococcaceae;g:Faecalibacterium;s:Faecalibacterium_prausnitzii* |  |  |  |  |
|  |  |  | ASV_2772 | 0.20 | 0.04 | 11 | 9 | 2.45 | Clustered | 11 | 0.88 | 1.67 | 19774 | 0.13 | *d:Bacteria;p:Firmicutes;c:Clostridia;o:Clostridiales;*  *f:Ruminococcaceae;g:Faecalibacterium;s:Faecalibacterium_prausnitzii* |  |  |  |  |
|  |  |  | ASV_4245 | 0.17 | 1.00 | 2 | 10 | 8.00 | Clustered | 2 | 0.86 | 2.00 | 0 | 0.62 | *d:Bacteria;p:Firmicutes;c:Clostridia;o:Clostridiales;*  *f:Ruminococcaceae;g:Faecalibacterium;s:Faecalibacterium_prausnitzii* |  |  |  |  |
|  |  | Cluster 5 | ASV_5254 | 0.28 | 0.02 | 10 | 7 | 12.10 | Clustered | 10 | 0.93 | 2.00 | 61074 | 0.18 | *d:Bacteria;p:Firmicutes;c:Clostridia;o:Clostridiales;*  *f:Eubacteriaceae;g:Eubacterium;s:Eubacterium_desmolans* |  |  |  |  |
|  |  |  | ASV_174 | 0.26 | 0.33 | 3 | 8 | 19.00 | Clustered | 3 | 0.92 | 2.00 | 708 | 0.36 | *d:Bacteria;p:Bacteroidetes;c:Bacteroidia;o:Bacteroidales;*  *f:Bacteroidaceae;g:Bacteroides;s:Bacteroides_caccae* |  |  |  |  |
|  |  |  | ASV_852 | 0.29 | 0.00 | 35 | 8 | 6.51 | Seed | 35 | 0.93 | 2.00 | 348098 | 0.066 | *d:Bacteria;p:Firmicutes;c:Clostridia;o:Clostridiales;*  *f:Ruminococcaceae;g:Intestinimonas;s:Intestinimonas_butyriciproducens* |  |  |  |  |
