# Supplementary material for: Riboflavin Supplementation Promotes Butyrate Production in the Absence of Gross Compositional Changes in the Gut Microbiota
Source: Antioxid Redox Signal. 2023 Feb 14;38(4):282–97. doi: 10.1089/ars.2022.0033 (PMC9986023; doi:10.1089/ars.2022.0033)
Supplement: Supplemental data [file Suppl_FigS6.docx]

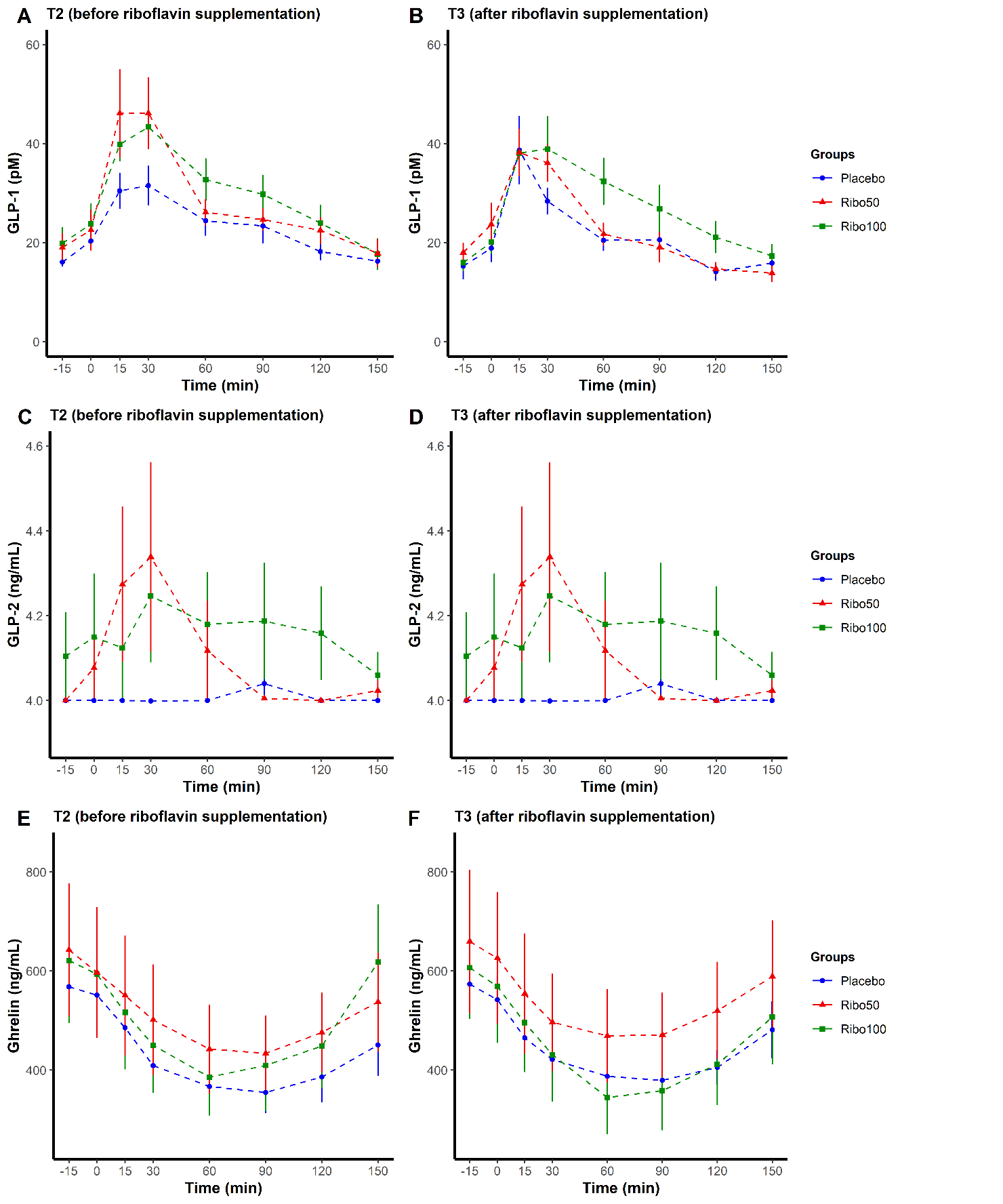
 **Supplementary Figure 6.** Oral glucose tolerance test results of GLP-1,GLP2 and ghrelin concentration at T2 before (A, C, E ) and T3 after (B, D, F ) riboflavin supplementation.
