# Supplementary material for: Riboflavin Supplementation Promotes Butyrate Production in the Absence of Gross Compositional Changes in the Gut Microbiota
Source: Antioxid Redox Signal. 2023 Feb 14;38(4):282–97. doi: 10.1089/ars.2022.0033 (PMC9986023; doi:10.1089/ars.2022.0033)
Supplement: Supplemental data [file Suppl_TableS4.docx]

**Supplementary Table 4**. Clinical characteristics of the study population.

| **Clinical characteristics** | **Placebo** | | **Ribo50** | | **Ribo100** | |
| --- | --- | --- | --- | --- | --- | --- |
|  | **T0** | **T3** | **T0** | **T3** | **T0** | **T3** |
| **ALAT (U/L)** | 17 (22 - 13) | 17 (21 - 13) | 18 (23 - 14) | 17 (21 - 14) | 16 (23.25 - 13) | 16.5 (19 - 13) |
| **ASAT (U/L)** | 22 (26 - 19) | 19 (22.5 - 19) | 22 (27 - 19) | 22 (26 - 19) | 20 (25.25 - 18) | 20.5 (24 - 18) |
| **Albumin (g/L)** | 47.4 (49.45 - 44.8) | 46 (47.7 - 43.25) | 46.9 (49 - 43.4) | 44.8 (47.3 - 42.8) | 46.05 (46.95 - 44.08) | 45 (46.4 - 44.1) |
| **Alkaline phosphatase (U/L)** | 57 (64.5 - 48) | 56 (69 - 44.5) | 68 (77 - 50) | 63 (78 - 53) | 63 (69.5 - 52.75) | 59.5 (65.25 - 50) |
| **Amylase (U/L)** | 66 (79.5 - 57) | 63 (73.5 - 51.5) | 71 (84 - 49) | 71 (88 - 50) | 57.5 (80 - 45.75) | 55 (75 - 42.5) |
| **Basophiles (%)** | 0.7 (1 - 0.5) | 0.7 (0.75 - 0.55) | 0.7 (0.9 - 0.5) | 0.8 (1.1 - 0.5) | 0.7 (0.925 - 0.5) | 0.7 (1 - 0.6) |
| **Basophiles (10^9/L)** | 0.05 (0.06 - 0.03) | 0.04 (0.05 - 0.03) | 0.04 (0.05 - 0.03) | 0.04 (0.05 - 0.03) | 0.05 (0.0525 - 0.03) | 0.04 (0.06 - 0.03) |
| **Sedimentation rate (mm/uur)** | 5 (13.5 - 2.5) | 5 (10 - 2) | 5 (11 - 3) | 6 (11 - 3) | 3 (7 - 2) | 4 (6 - 2) |
| **Bilirubin total (umol/L)** | 8 (12.05 - 5.45) | 7.9 (12.75 - 6.4) | 8.8 (11.7 - 7) | 8.9 (10.6 - 7.5) | 9.35 (10.73 - 6.5) | 9.35 (13.73 - 5.85) |
| **Chloride (mmol/L)** | 102.6 (103.75 - 101.4) | 103.1 (103.7 - 101.9) | 102.4 (103.8 - 101.1) | 102.9 (104 - 101.5) | 102.4 (103.9 - 101.3) | 103.3 (104.43 - 101.9) |
| **CRP (mg/L)** | 0.6 (1.05 - 0.3) | 0.3 (1.75 - 0.3) | 0.4 (1 - 0.3) | 0.4 (0.8 - 0.3) | 0.65 (1.2 - 0.3) | 0.35 (0.725 - 0.3) |
| **EGFR (ml/min*1.73m^2^)** | 113.6 (115.65 - 109.25) | 111.5 (114.70 - 102.55) | 111.1 (117.1 - 107.3) | 112.2 (117.4 - 106.1) | 113.4 (118.93 - 107.53) | 113.4 (116.03 - 109.3) |
| **Eosinophils (%)** | 2.2 (3.55 - 1.5) | 2.4 (3.75 - 1.9) | 2.2 (3.6 - 1.4) | 2.2 (3.8 - 1.6) | 2.3 (3.63 - 1.525) | 2.5 (3.8 - 1.58) |
| **Eosinophils (10^9/L)** | 0.15 (0.23 - 0.10) | 0.14 (0.21 - 0.11) | 0.14 (0.2 - 0.07) | 0.13 (0.18 - 0.09) | 0.135 (0.21 - 0.09) | 0.135 (0.21 - 0.09) |
| **Erythrocytes (10^12/L)** | 4.67 (4.98 - 4.28) | 4.42 (4.70 - 4.14) | 4.6 (4.97 - 4.28) | 4.22 (4.69 - 3.95) | 4.695 (4.90 - 4.515) | 4.48 (4.71 - 4.27) |
| **Gamma-GT (U/L)** | 14 (17 - 11) | 12 (15 - 10) | 14 (23 - 11) | 14 (20 - 11) | 15 (17 - 12) | 14 (18 - 11.75) |
| **Glucose (mmol/L)** | 4.73 (5.22 - 4.10) | 4.68 (4.98 - 4.37) | 4.68 (5.14 - 4.28) | 4.74 (5.07 - 4.49) | 4.74 (5.285 - 4.25) | 4.76 (4.92 - 4.42) |
| **Hb (mmol/L)** | 8.5 (8.95 -8.05) | 8 (8.4 - 7.8) | 8.2 (8.9 - 7.9) | 7.8 (8.5 - 7.4) | 8.65 (8.9 - 8.3) | 8.25 (8.7 - 8.05) |
| **Hemolytic index Li-Heparin** | 3 (4 - 1.5) | 2 (3 - 1) | 3 (4 - 1) | 2 (3 - 1) | 2 (4.25 - 1) | 2.5 (4.25 - 1) |
| **Ht (L/L)** | 0.42 (0.43 - 0.39) | 0.39 (0.41 - 0.37) | 0.4 (0.43 - 0.38) | 0.37 (0.42 - 0.36) | 0.41 (0.43 - 0.4) | 0.4 (0.41 - 0.38) |
| **Icteric index Li-heparin** | 16 (20.5 - 14) | 15 (23 - 14) | 16 (20 - 14) | 17 (20 - 15) | 16.5 (19.25 - 13) | 17 (22.5 - 13.75) |
| **Iron (umol/L)** | 17.57 (21.74 - 13.69) | 17.35 (25.27 - 12.105) | 17.82 (21.21 - 11.52) | 17.11 (24.93 - 14.79) | 15.95 (21 - 11.55) | 18.135 (20.2 - 14.87) |
| **Iron binding capacity (umol/L)** | 66 (73.5-61.5) | 66 (72 - 58.5) | 71 (78 - 62) | 70 (77 - 61) | 65 (72.25 - 61) | 65 (69.5 - 62.75) |
| **Potassium (mmol/L)** | 4.02 (4.23 - 3.73) | 3.82 (3.98 - 3.72) | 4 (4.19 - 3.75) | 3.88 (4.05 - 3.77) | 3.95 (4.16 - 3.82) | 3.81 (3.98 - 3.70) |
| **Creatinine (umol/L)** | 74.1 (78.4 - 67.95) | 74.4 (81.2 - 68.45) | 74.2 (81.9 - 64.5) | 72.8 (80.4 - 65.8) | 71.6 (79.9 - 63.55) | 71.5 (78.9 - 67.7) |
| **Leukocytes (10^9/L)** | 6.52 (7.22 - 5.52) | 6.22 (6.97 - 5.14) | 5.54 (6.73 - 4.82) | 5.12 (5.84 - 4.55) | 6.595 (7.77 - 5.18) | 5.905 (7.4 - 4.80) |
| **Lipemic index Li-heparin** | 19 (26.5 - 15) | 14 (23 - 11.5) | 18 (26 - 12) | 18 (26 - 12) | 13 (18.25 - 11) | 12 (16.25 - 9.75) |
| **Lymphocytes (%)** | 33.5 (36.9 - 29.35) | 33.1 (36.6 - 26.1) | 32.9 (37.8 - 31.5) | 36.7 (40.5 - 31.6) | 31.85 (38.68 - 26.85) | 35.3 (38.73 - 29.83) |
| **Lymphocytes (10^9/L)** | 2.08 (2.48 - 1.835) | 1.92 (2.15 - 1.715) | 1.92 (2.23 - 1.58) | 1.82 (2.13 - 1.47) | 1.965 (2.35 - 1.65) | 2 (2.62 - 1.62) |
| **MCV (fL)** | 88.2 (90.5 - 86.85) | 87.9 (90.25 - 86.55) | 87.7 (90 - 86.4) | 87.8 (90.8 - 85.7) | 88.05 (90.3 - 85.85) | 87.35 (90.1 - 85.45) |
| **Monocytes (%)** | 8.2 (9.1 - 6.55) | 7.5 (9.1 - 6.6) | 7.7 (8.7 - 6.5) | 7.9 (9.8 - 6.3) | 7.7 (9.73 - 6.4) | 7.75 (9.23 - 6.4) |
| **Monocytes (10^9/L)** | 0.51 (0.57 - 0.41) | 0.45 (0.53 - 0.39) | 0.41 (0.54 - 0.37) | 0.38 (0.48 - 0.32) | 0.5 (0.65 - 0.36) | 0.43 (0.59 - 0.32) |
| **Sodium (mmol/L)** | 140.1 (140.95 - 139.5) | 140.5 (141.7 - 139.45) | 140.4 (141.6 - 139.5) | 139.8 (141.3 - 139.1) | 141.15 (141.5 - 139.73) | 139.85 (140.8 - 138.95) |
| **Neutrophils (%)** | 55.4 (59.85 - 51.05) | 54.4 (60.3 - 47.6) | 54.3 (58.1 - 50) | 51.2 (56.4 - 48.1) | 55.7 (62.225 - 44.525) | 53.3 (58.83 - 48.03) |
| **Neutrophils (10^9/L)** | 3.51 (3.97 - 3.13) | 3.4 (4.03 - 2.45) | 2.89 (3.55 - 2.43) | 2.64 (3.13 - 2.23) | 3.77 (4.56 - 2.47) | 3.01 (4.09 - 2.47) |
| **Immature granulocytes (%)** | 0.3 (0.4 - 0.2) | 0.3 (0.4 - 0.2) | 0.2 (0.4 - 0.2) | 0.3 (0.4 - 0.2) | 0.3 (0.4 - 0.2) | 0.3 (0.4 - 0.2) |
| **Immature granulocytes (10^9/L)** | 0.02 (0.02 - 0.01) | 0.02 (0.03 - 0.01) | 0.01 (0.02 - 0.01) | 0.01 (0.02 - 0.01) | 0.02 (0.03 - 0.01) | 0.02 (0.02 - 0.01) |
| **Transferrin (g/L)** | 2.61 (2.93 - 2.45) | 2.61 (2.87-2.32) | 2.81 (3.08-2.46) | 2.78 (3.05-2.43) | 2.575 (2.87-2.44) | 2.575 (2.76-2.49) |
| **Thrombocytes (10^9/L)** | 254 (287.5 - 237.5) | 268 (285-241) | 249 (262-221) | 240 (272-222) | 263 (295.25-201) | 249 (289.25-201) |
| **Urea (mmol/L)** | 4.54 (5.09 - 4.13) | 4.16 (4.79-3.74) | 4.24 (4.94-3.63) | 4.46 (5.12-3.87) | 4.275 (4.57-3.52) | 4.115 (4.93-3.65) |

All biomarkers are presented as median (interquartile range). ALAT, alanine aminotransferase; ASAT, aspartate aminotransferase; CRP, C-reactive protein; EGFR, estimated glomerular filtration rate; Gamma-GT, Gamma-glutamyl Transferase; Hb, Hemoglobin; Ht, Hematocrit; MCV, Mean corpuscular volume.
