# Supplementary material for: Riboflavin Supplementation Promotes Butyrate Production in the Absence of Gross Compositional Changes in the Gut Microbiota
Source: Antioxid Redox Signal. 2023 Feb 14;38(4):282–97. doi: 10.1089/ars.2022.0033 (PMC9986023; doi:10.1089/ars.2022.0033)
Supplement: Supplemental data [file Suppl_Data.docx]

**Supplementary Methods**

***Riboflavin capsules and placebo***

The investigational product is a nutritional supplement named “Riboflavin”. Riboflavin (E 101, vitamin B2) is authorized as a food additive in the European Union (EU) in accordance with Annex II to Regulation (EC) No 1333/2008 and 1129/2011. Riboflavin Universal is a yellow to orange-yellow free-flowing powder, practically odorless, with a bitter taste. It melts at about 280°C with decomposition. It is produced by fermentation using a genetically modified microorganism. Riboflavin Universal is purified and free of detectable DNA. The full chemical name is 7,8-dimethyl-10-(1-D-ribityl)-isoalloxazine; 7,8-dimethyl-10-(D-ribo-2,3,4,5-tetrahydroxy-pentyl)isoalloxazine. All riboflavin supplements (hard gel capsules) will be consumed orally. The daily dose will be ingested by taking one capsule per day at breakfast.

The placebo formulation is 250 mg pregelatinized starch plus 0,5% silica. Silica is a common additive in the production of foods, where it is used primarily as a flow agent in powdered foods, or to adsorb water in hygroscopic applications. It contains no other active ingredients, but is matched with riboflavin in terms of appearance (volume, color, and consistency), solubility, smell, and taste, using the hard gelatin capsules of the color chocolate brown.

***Bacterial probes used in the fluorescent in-situ hybridization (FISH)***

| Target | **Probe** | **Sequence 3’-5’** |
| --- | --- | --- |
| Total bacteria | EUB338 | *TGAGGATGCCCTCCGTCG* |
| *Faecalibacterium prausnitzii* | Fprau645 | *CAAAAAGAACTCATCACGTCTCC* |
| *Enterobacteriacea* | Ec1531 | *ACTACTGCTCCGTGATGCCAC* |
| *Clostridium* group XIVa | Erec482 | *GCCATGRACTGATTCTTCG* |
| *Roseburia* | Rint623 | *GGGCCATGACGTAACCTT* |

***Blood sample collection/preparation***

Blood was collected by qualified research assistants using a standardized protocol. Blood was drawn from the antecubital vein in the arm. During screening (T0) and T3 three tubes of 6 ml, 4 ml and 2 ml were collected for chemistry and hematology measurements. During T2 and T3 (oGTT test days), blood samples were collected into ice chilled EDTA tubes at regular time intervals as depicted in Figure 3 (-15, 0, 15, 30, 60, 90, 120, 150).

A portable cooled centrifuge is available to use at the study locations. Serum containing tubes were stored in the dark for 20 minutes at room temperature and centrifuged thereafter. Ice chilled plasma tubes only containing EDTA (during oGTT test days) were stored on ice immediately after blood drawing and centrifuged promptly after blood drawing at 3200 rpm for 15 min at 4°C to prevent temperature and time-dependent influences. All samples were immediately stored on dry ice after processing and were stored at -80ºC at the UMC Groningen until analysis.

***Primers and barcodes used in this study***

Comment on table with the primers and barcodes used in this study, partly cited from Bartram et al.; Generation of multi-million 16S rRNA gene libraries from complex microbial communities by assembling paired-end Illumina reads. Lowercase letters denote adapter sequences necessary for binding to the flow cell, underlined lowercase are binding sites for the Illumina sequencing primers, bold uppercase highlight the index sequences (all the indexes were obtained from Illumina) and regular uppercase are the V3 region forward primer 341F and the V4 region reverse primers 806R. The inclusion of four maximally degenerated bases (“NNNN”) maximizes diversity during the first four bases of the run. Diversity is important for identifying unique clusters and base-calling accuracy.

| V3F_modified |  | aatgatacggcgaccaccgagatct |  | acactctttccctacacgacgctcttccgatct | NNNNCCTACGGGAGGCAGCAG |
| --- | --- | --- | --- | --- | --- |
| V4R | 1 | caagcagaagacggcatacgagat | **ATCACG** | gtgactggagttcagacgtgtgctcttccgatct | GGACTACHVGGGTWTCTAAT |
| V4R | 2 | caagcagaagacggcatacgagat | **CGATGT** | gtgactggagttcagacgtgtgctcttccgatct | GGACTACHVGGGTWTCTAAT |
| V4R | 3 | caagcagaagacggcatacgagat | **TTAGGC** | gtgactggagttcagacgtgtgctcttccgatct | GGACTACHVGGGTWTCTAAT |
| V4R | 4 | caagcagaagacggcatacgagat | **TGACCA** | gtgactggagttcagacgtgtgctcttccgatct | GGACTACHVGGGTWTCTAAT |
| V4R | 5 | caagcagaagacggcatacgagat | **ACAGTG** | gtgactggagttcagacgtgtgctcttccgatct | GGACTACHVGGGTWTCTAAT |
| V4R | 6 | caagcagaagacggcatacgagat | **GCCAAT** | gtgactggagttcagacgtgtgctcttccgatct | GGACTACHVGGGTWTCTAAT |
| V4R | 7 | caagcagaagacggcatacgagat | **CAGATC** | gtgactggagttcagacgtgtgctcttccgatct | GGACTACHVGGGTWTCTAAT |
| V4R | 8 | caagcagaagacggcatacgagat | **ACTTGA** | gtgactggagttcagacgtgtgctcttccgatct | GGACTACHVGGGTWTCTAAT |
| V4R | 9 | caagcagaagacggcatacgagat | **GATCAG** | gtgactggagttcagacgtgtgctcttccgatct | GGACTACHVGGGTWTCTAAT |
| V4R | 10 | caagcagaagacggcatacgagat | **TAGCTT** | gtgactggagttcagacgtgtgctcttccgatct | GGACTACHVGGGTWTCTAAT |
| V4R | 11 | caagcagaagacggcatacgagat | **GGCTAC** | gtgactggagttcagacgtgtgctcttccgatct | GGACTACHVGGGTWTCTAAT |
| V4R | 12 | caagcagaagacggcatacgagat | **CTTGTA** | gtgactggagttcagacgtgtgctcttccgatct | GGACTACHVGGGTWTCTAAT |
| V4R | 13 | caagcagaagacggcatacgagat | **AGTACG** | gtgactggagttcagacgtgtgctcttccgatct | GGACTACHVGGGTWTCTAAT |
| V4R | 14 | caagcagaagacggcatacgagat | **TCAGTC** | gtgactggagttcagacgtgtgctcttccgatct | GGACTACHVGGGTWTCTAAT |
| V4R | 15 | caagcagaagacggcatacgagat | **TTGAGC** | gtgactggagttcagacgtgtgctcttccgatct | GGACTACHVGGGTWTCTAAT |
| V4R | 16 | caagcagaagacggcatacgagat | **AAGCGA** | gtgactggagttcagacgtgtgctcttccgatct | GGACTACHVGGGTWTCTAAT |
| V4R | 18 | caagcagaagacggcatacgagat | **GGTTGT** | gtgactggagttcagacgtgtgctcttccgatct | GGACTACHVGGGTWTCTAAT |
| V4R | 19 | caagcagaagacggcatacgagat | **TGAGGT** | gtgactggagttcagacgtgtgctcttccgatct | GGACTACHVGGGTWTCTAAT |
| V4R | 20 | caagcagaagacggcatacgagat | **TACCGT** | gtgactggagttcagacgtgtgctcttccgatct | GGACTACHVGGGTWTCTAAT |
| V4R | 21 | caagcagaagacggcatacgagat | **CCAACT** | gtgactggagttcagacgtgtgctcttccgatct | GGACTACHVGGGTWTCTAAT |
| V4R | 22 | caagcagaagacggcatacgagat | **AGAGAG** | gtgactggagttcagacgtgtgctcttccgatct | GGACTACHVGGGTWTCTAAT |
| V4R | 23 | caagcagaagacggcatacgagat | **CACTTG** | gtgactggagttcagacgtgtgctcttccgatct | GGACTACHVGGGTWTCTAAT |
| V4R | 24 | caagcagaagacggcatacgagat | **TCAAGG** | gtgactggagttcagacgtgtgctcttccgatct | GGACTACHVGGGTWTCTAAT |
| V4R | 25 | caagcagaagacggcatacgagat | **AGTGGT** | gtgactggagttcagacgtgtgctcttccgatct | GGACTACHVGGGTWTCTAAT |
| V4R | 26 | caagcagaagacggcatacgagat | **GACACT** | gtgactggagttcagacgtgtgctcttccgatct | GGACTACHVGGGTWTCTAAT |
| V4R | 27 | caagcagaagacggcatacgagat | **CCTTCT** | gtgactggagttcagacgtgtgctcttccgatct | GGACTACHVGGGTWTCTAAT |
| V4R | 28 | caagcagaagacggcatacgagat | **GGATAA** | gtgactggagttcagacgtgtgctcttccgatct | GGACTACHVGGGTWTCTAAT |
| V4R | 29 | caagcagaagacggcatacgagat | **CCTTAA** | gtgactggagttcagacgtgtgctcttccgatct | GGACTACHVGGGTWTCTAAT |
| V4R | 30 | caagcagaagacggcatacgagat | **CAAGAA** | gtgactggagttcagacgtgtgctcttccgatct | GGACTACHVGGGTWTCTAAT |
| V4R | 31 | caagcagaagacggcatacgagat | **GTTGAA** | gtgactggagttcagacgtgtgctcttccgatct | GGACTACHVGGGTWTCTAAT |
| V4R | 32 | caagcagaagacggcatacgagat | **TCACAA** | gtgactggagttcagacgtgtgctcttccgatct | GGACTACHVGGGTWTCTAAT |
| V4R | 33 | caagcagaagacggcatacgagat | **AGTCAA** | gtgactggagttcagacgtgtgctcttccgatct | GGACTACHVGGGTWTCTAAT |
| V4R | 34 | caagcagaagacggcatacgagat | **CGAATA** | gtgactggagttcagacgtgtgctcttccgatct | GGACTACHVGGGTWTCTAAT |
| V4R | 35 | caagcagaagacggcatacgagat | **GCTATA** | gtgactggagttcagacgtgtgctcttccgatct | GGACTACHVGGGTWTCTAAT |
| V4R | 36 | caagcagaagacggcatacgagat | **GAGTTA** | gtgactggagttcagacgtgtgctcttccgatct | GGACTACHVGGGTWTCTAAT |
| V4R | 37 | caagcagaagacggcatacgagat | **TTGGTA** | gtgactggagttcagacgtgtgctcttccgatct | GGACTACHVGGGTWTCTAAT |
| V4R | 38 | caagcagaagacggcatacgagat | **AACGTA** | gtgactggagttcagacgtgtgctcttccgatct | GGACTACHVGGGTWTCTAAT |
| V4R | 39 | caagcagaagacggcatacgagat | **GTACTA** | gtgactggagttcagacgtgtgctcttccgatct | GGACTACHVGGGTWTCTAAT |
| V4R | 40 | caagcagaagacggcatacgagat | **CATCTA** | gtgactggagttcagacgtgtgctcttccgatct | GGACTACHVGGGTWTCTAAT |
| V4R | 41 | caagcagaagacggcatacgagat | **TGTAGA** | gtgactggagttcagacgtgtgctcttccgatct | GGACTACHVGGGTWTCTAAT |
| V4R | 42 | caagcagaagacggcatacgagat | **ATCAGA** | gtgactggagttcagacgtgtgctcttccgatct | GGACTACHVGGGTWTCTAAT |
| V4R | 43 | caagcagaagacggcatacgagat | **ACATGA** | gtgactggagttcagacgtgtgctcttccgatct | GGACTACHVGGGTWTCTAAT |
| V4R | 44 | caagcagaagacggcatacgagat | **TAGACA** | gtgactggagttcagacgtgtgctcttccgatct | GGACTACHVGGGTWTCTAAT |
| V4R | 45 | caagcagaagacggcatacgagat | **GAGAAT** | gtgactggagttcagacgtgtgctcttccgatct | GGACTACHVGGGTWTCTAAT |
| V4R | 47 | caagcagaagacggcatacgagat | **AGGTAT** | gtgactggagttcagacgtgtgctcttccgatct | GGACTACHVGGGTWTCTAAT |
| V4R | 48 | caagcagaagacggcatacgagat | **TTGCAT** | gtgactggagttcagacgtgtgctcttccgatct | GGACTACHVGGGTWTCTAAT |
| V4R | 49 | caagcagaagacggcatacgagat | **TGGATT** | gtgactggagttcagacgtgtgctcttccgatct | GGACTACHVGGGTWTCTAAT |
| V4R | 50 | caagcagaagacggcatacgagat | **ACCATT** | gtgactggagttcagacgtgtgctcttccgatct | GGACTACHVGGGTWTCTAAT |
| V4R | 51 | caagcagaagacggcatacgagat | **CTAGTT** | gtgactggagttcagacgtgtgctcttccgatct | GGACTACHVGGGTWTCTAAT |
| V4R | 53 | caagcagaagacggcatacgagat | **TCTCTT** | gtgactggagttcagacgtgtgctcttccgatct | GGACTACHVGGGTWTCTAAT |
| V4R | 54 | caagcagaagacggcatacgagat | **GTAAGT** | gtgactggagttcagacgtgtgctcttccgatct | GGACTACHVGGGTWTCTAAT |
| V4R | 55 | caagcagaagacggcatacgagat | **CAATGT** | gtgactggagttcagacgtgtgctcttccgatct | GGACTACHVGGGTWTCTAAT |
| V4R | 57 | caagcagaagacggcatacgagat | **ATGACT** | gtgactggagttcagacgtgtgctcttccgatct | GGACTACHVGGGTWTCTAAT |
| V4R | 58 | caagcagaagacggcatacgagat | **ACTTCT** | gtgactggagttcagacgtgtgctcttccgatct | GGACTACHVGGGTWTCTAAT |
| V4R | 59 | caagcagaagacggcatacgagat | **CATAAG** | gtgactggagttcagacgtgtgctcttccgatct | GGACTACHVGGGTWTCTAAT |
| V4R | 60 | caagcagaagacggcatacgagat | **TTCTAG** | gtgactggagttcagacgtgtgctcttccgatct | GGACTACHVGGGTWTCTAAT |
| V4R | 61 | caagcagaagacggcatacgagat | **AAGATG** | gtgactggagttcagacgtgtgctcttccgatct | GGACTACHVGGGTWTCTAAT |
| V4R | 62 | caagcagaagacggcatacgagat | **TATGTG** | gtgactggagttcagacgtgtgctcttccgatct | GGACTACHVGGGTWTCTAAT |
| V4R | 63 | caagcagaagacggcatacgagat | **AATTGG** | gtgactggagttcagacgtgtgctcttccgatct | GGACTACHVGGGTWTCTAAT |
| V4R | 64 | caagcagaagacggcatacgagat | **TAATCG** | gtgactggagttcagacgtgtgctcttccgatct | GGACTACHVGGGTWTCTAAT |
| V4R | 65 | caagcagaagacggcatacgagat | **ACTAAC** | gtgactggagttcagacgtgtgctcttccgatct | GGACTACHVGGGTWTCTAAT |
| V4R | 66 | caagcagaagacggcatacgagat | **TGTTAC** | gtgactggagttcagacgtgtgctcttccgatct | GGACTACHVGGGTWTCTAAT |
| V4R | 67 | caagcagaagacggcatacgagat | **ATACAC** | gtgactggagttcagacgtgtgctcttccgatct | GGACTACHVGGGTWTCTAAT |
| V4R | 68 | caagcagaagacggcatacgagat | **CTTATC** | gtgactggagttcagacgtgtgctcttccgatct | GGACTACHVGGGTWTCTAAT |
| V4R | 69 | caagcagaagacggcatacgagat | **AGATTC** | gtgactggagttcagacgtgtgctcttccgatct | GGACTACHVGGGTWTCTAAT |
| V4R | 71 | caagcagaagacggcatacgagat | **TGCGAA** | gtgactggagttcagacgtgtgctcttccgatct | GGACTACHVGGGTWTCTAAT |
| V4R | 73 | caagcagaagacggcatacgagat | **CTGTCA** | gtgactggagttcagacgtgtgctcttccgatct | GGACTACHVGGGTWTCTAAT |
| V4R | 74 | caagcagaagacggcatacgagat | **GCAGAT** | gtgactggagttcagacgtgtgctcttccgatct | GGACTACHVGGGTWTCTAAT |
| V4R | 75 | caagcagaagacggcatacgagat | **TCGTGT** | gtgactggagttcagacgtgtgctcttccgatct | GGACTACHVGGGTWTCTAAT |
| V4R | 76 | caagcagaagacggcatacgagat | **GAACCT** | gtgactggagttcagacgtgtgctcttccgatct | GGACTACHVGGGTWTCTAAT |
| V4R | 77 | caagcagaagacggcatacgagat | **GTCATG** | gtgactggagttcagacgtgtgctcttccgatct | GGACTACHVGGGTWTCTAAT |
| V4R | 78 | caagcagaagacggcatacgagat | **GATAGC** | gtgactggagttcagacgtgtgctcttccgatct | GGACTACHVGGGTWTCTAAT |
| V4R | 79 | caagcagaagacggcatacgagat | **AAGTCC** | gtgactggagttcagacgtgtgctcttccgatct | GGACTACHVGGGTWTCTAAT |
| V4R | 80 | caagcagaagacggcatacgagat | **ATTGCC** | gtgactggagttcagacgtgtgctcttccgatct | GGACTACHVGGGTWTCTAAT |
| V4R | 81 | caagcagaagacggcatacgagat | **CCGAGA** | gtgactggagttcagacgtgtgctcttccgatct | GGACTACHVGGGTWTCTAAT |
| V4R | 82 | caagcagaagacggcatacgagat | **CGCTGA** | gtgactggagttcagacgtgtgctcttccgatct | GGACTACHVGGGTWTCTAAT |
| V4R | 83 | caagcagaagacggcatacgagat | **GGCACA** | gtgactggagttcagacgtgtgctcttccgatct | GGACTACHVGGGTWTCTAAT |
| V4R | 84 | caagcagaagacggcatacgagat | **CGTGCA** | gtgactggagttcagacgtgtgctcttccgatct | GGACTACHVGGGTWTCTAAT |
| V4R | 85 | caagcagaagacggcatacgagat | **GGCCTT** | gtgactggagttcagacgtgtgctcttccgatct | GGACTACHVGGGTWTCTAAT |
| V4R | 86 | caagcagaagacggcatacgagat | **CCTGGT** | gtgactggagttcagacgtgtgctcttccgatct | GGACTACHVGGGTWTCTAAT |
| V4R | 87 | caagcagaagacggcatacgagat | **CAGGCT** | gtgactggagttcagacgtgtgctcttccgatct | GGACTACHVGGGTWTCTAAT |
| V4R | 88 | caagcagaagacggcatacgagat | **GTCGCT** | gtgactggagttcagacgtgtgctcttccgatct | GGACTACHVGGGTWTCTAAT |
| V4R | 89 | caagcagaagacggcatacgagat | **GCGTAG** | gtgactggagttcagacgtgtgctcttccgatct | GGACTACHVGGGTWTCTAAT |
| V4R | 90 | caagcagaagacggcatacgagat | **CTGGAG** | gtgactggagttcagacgtgtgctcttccgatct | GGACTACHVGGGTWTCTAAT |
| V4R | 91 | caagcagaagacggcatacgagat | **CTACGG** | gtgactggagttcagacgtgtgctcttccgatct | GGACTACHVGGGTWTCTAAT |
| V4R | 92 | caagcagaagacggcatacgagat | **ACACCG** | gtgactggagttcagacgtgtgctcttccgatct | GGACTACHVGGGTWTCTAAT |
| V4R | 93 | caagcagaagacggcatacgagat | **GTTCCG** | gtgactggagttcagacgtgtgctcttccgatct | GGACTACHVGGGTWTCTAAT |
| V4R | 94 | caagcagaagacggcatacgagat | **CAGCAC** | gtgactggagttcagacgtgtgctcttccgatct | GGACTACHVGGGTWTCTAAT |
| V4R | 95 | caagcagaagacggcatacgagat | **CCGTTC** | gtgactggagttcagacgtgtgctcttccgatct | GGACTACHVGGGTWTCTAAT |
| V4R | 96 | caagcagaagacggcatacgagat | **GCATCC** | gtgactggagttcagacgtgtgctcttccgatct | GGACTACHVGGGTWTCTAAT |
| V4R | 97 | caagcagaagacggcatacgagat | **TACGCC** | gtgactggagttcagacgtgtgctcttccgatct | GGACTACHVGGGTWTCTAAT |
